# Establishing an untargeted lipidomics workflow for cellular analysis: insights into endothelial cell function in anaphylaxis

**DOI:** 10.3389/fimmu.2026.1711640

**Published:** 2026-03-04

**Authors:** María Isabel Delgado Dolset, Andrea Escolar-Peña, Sergio Fernández-Bravo, Antonio J. García-Cívico, Lucía Pajares, René Neuhaus, Rosario González-Mendiola, Coral Barbas, Domingo Barber, José Julio Laguna, María M. Escribese, Vanesa Esteban, Alma Villaseñor

**Affiliations:** 1 Centro de Metabolómica y Bioanálisis (CEMBIO), Facultad de Farmacia, Universidad San Pablo-CEU, CEU Universities, Boadilla del Monte, Spain; 2 Departamento de Ciencias Médicas Básicas, Facultad de Medicina, Instituto de Medicina Molecular Aplicada – Nemesio Díez (IMMA-ND), Universidad San Pablo-CEU, CEU Universities, Madrid, Spain; 3 Department of Allergy and Immunology, IIS-Fundación Jiménez Díaz, Universidad Autónoma de Madrid (UAM), Madrid, Spain; 4 Allergy Unit, Allergo-Anaesthesia Unit, Hospital Central de la Cruz Roja, Madrid, Spain; 5 Faculty of Biomedical and Health Sciences, Alfonso X El Sabio University, Madrid, Spain

**Keywords:** cell count interval, correlations, immunometabolism, LC-MS, lipidomics, metabolites

## Abstract

**Background:**

Cell metabolomics, including lipidomics, presents several challenges regarding analyzing limited cell populations and distinguishing cellular metabolites from background signals originated from a stimuli or after a treatment. To address this, we have developed a novel workflow for untargeted cell lipidomics analysis.

**Methods:**

To study the impact of varying input cell numbers on the outcomes of untargeted cell lipidomics analysis, CD3^+^ cells isolated from a healthy donor at 6 different cell counts (50k, 100k, 250k, 500k, 750k, and 1M) were analyzed by liquid chromatography coupled with quadrupole-time-of-flight mass spectrometry (LC-QTOF-MS) in positive and negative electrospray ionization (ESI+ and ESI-, respectively) modes. After data quality assurance (QA), Spearman correlation analyses were carried out to select chemical signals derived from cells (*ρ* ≥ 0.7, *p*-value < 0.05). Then, this methodology was applied to human microvascular dermal endothelial cells (HMVEC-d), where a cell number calibration curve including 4 cell counts (25k, 50k, 75k, and 100k) was incorporated alongside the experimental samples to enable the analysis of cell-derived chemical signals. Here, the lipid response of HMVEC-d after contact with sera from patients at baseline and during the acute stage of anaphylaxis triggered by three different mechanisms was explored.

**Results:**

For the CD3^+^ model, we found that although 1087 chemical signals (*k*) passed the QA, samples did not cluster according to their cell count when taking all signals into account. After correlation analyses, the widest cell count interval considered for correlation analyses (50k-to-1M; *k* = 70) showed clear clustering by cell number. The principal component analysis (PCA) models for ESI+ showed that for this cell count interval, the first component explained over 90% of the variance among samples. After applying the same methodology to HMVEC-d, we found *k* = 157 and *k* = 278 correlated chemical signals for ESI+ and ESI- in the cell curve (25k-100k). Statistical analysis identified 193 chemical signals that significantly (*p*-value < 0.05 and *p*-adjusted value < 0.2) differed between the acute and baseline stages of anaphylaxis. Without this correlation approach, 67 additional chemical signals would have been selected as significant. From the 193 chemical signals, 75 unique lipids were annotated, mainly including fatty acids, acyl carnitines, glycerophospholipids, and sphingolipids, all increased in the acute phase. These changes were associated with sphingolipid and glycosphingolipid metabolism, and ceramide and phospholipid signaling pathways.

**Conclusions:**

This workflow for cell lipidomics analysis allows the selection of lipids derived from the intracellular content regardless external sources, supporting specific intracellular metabolism profiling.

## Introduction

Immune cell function is tightly regulated by their metabolic state, with distinct metabolic pathways supporting activation, differentiation, and effector responses (1, 2). These metabolic programs not only supply energy but actively shape immune cell fate and function, making the metabolism a key player in immune regulation (3).

From the existing technologies, metabolomics is key for understanding the metabolic programs at the cellular level. Specifically, the use of mass spectrometry (MS)-based analytical techniques allows the detection of metabolites, including lipids, especially those in low concentrations, and untargeted analysis approaches allow the discovery of new molecular mechanisms. However, current limitations of cell lipidomics studies include the challenge of obtaining a sufficient number of cells for a comprehensive analysis of the cellular lipidome (4–6). Existing methodologies in literature usually describe the use of millions of cells (5, 7), which might be impossible when working with scarce populations, such as specific populations of immune cells from a blood sample (e.g., innate lymphoid cells). This is important because the analysis of low cell numbers can result in diminished MS sensitivity, limiting the detection and proper lipid annotation.

A recent study has pointed out the impact of using different numbers of cells in metabolomics, showing how much biological information is lost as the number of cells decreases (8). The authors also highlighted that specific characteristics of the cells (e.g., metabolic state or size) can significantly impact the outcome of the metabolomic analysis, aspects that are not usually considered when performing cell lipidomics.

Additionally, another important limitation is the distinction between lipids originated from cells and those from the extracellular compartment, including the culture medium or stimuli applied (e.g., stimulation of cells with serum from allergic patients). This aspect has not been systematically evaluated in previously published studies. Thus, given the importance of lipids in cell-cell communication and in the immune response (9), being able to distinguish the source of a specific lipid would be extremely helpful to determine whether it reflects intrinsic cellular processes or is merely an environmental artifact, potentially leading to misinterpretation of the lipidomic data.

**Figure 1:**
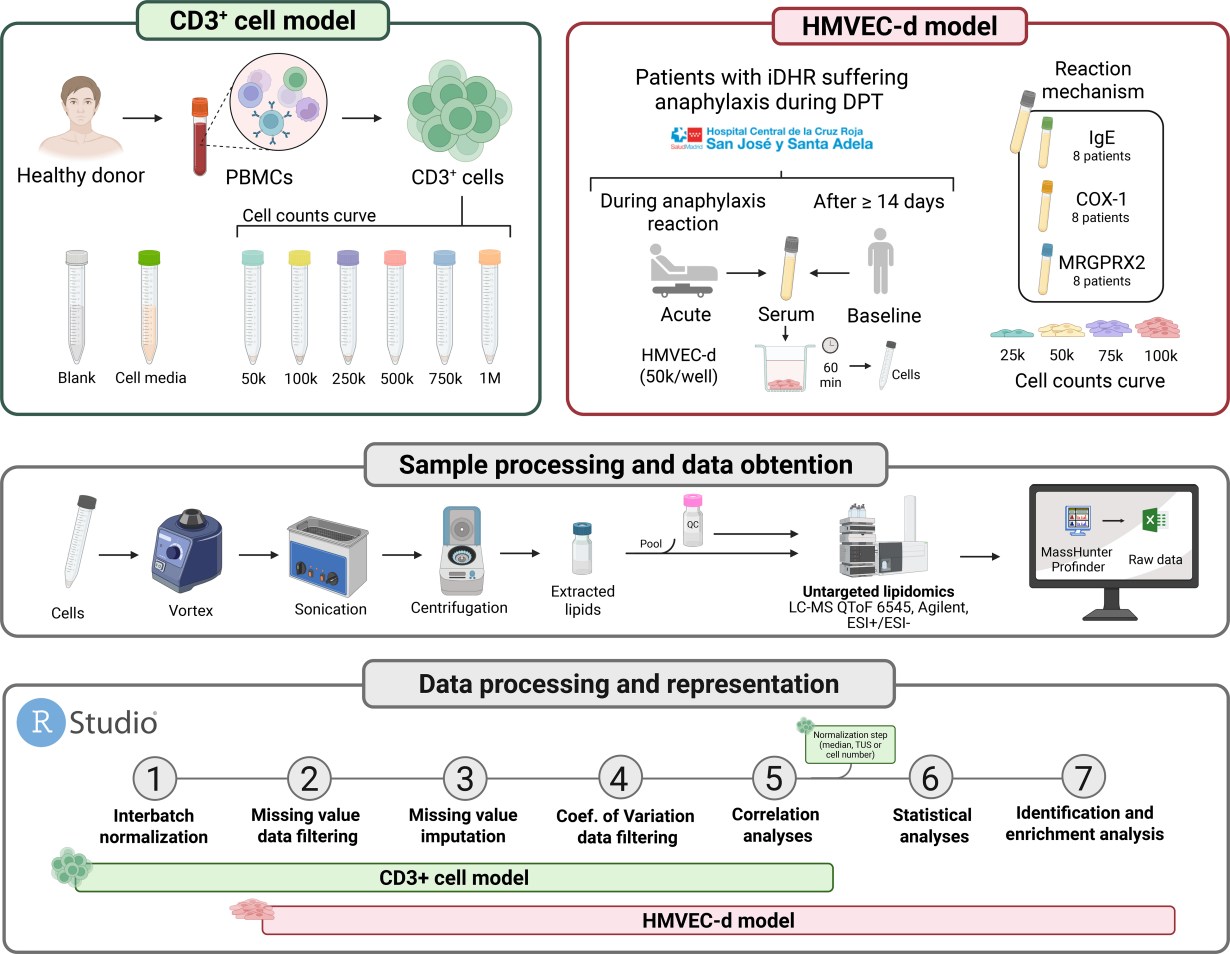
Experimental design. Workflow showing the sample collection (left: CD3^+^ cells isolated from whole blood; right: HMVEC-d treated with serum from anaphylactic patients), lipid extraction, and data collection and processing for the lipidomic analysis. DPT, drug provocation test; ESI, Electrospray Ionization; HMVEC-d, Human microvascular dermal endothelial cells; iDHR, immediate drug hypersensitivity reactions; LC-MS, Liquid Chromatography–Mass Spectrometry; MeOH, Methanol; PBMC, Peripheral Blood Mononuclear Cell; QC, quality control; TUS, total useful signal.

All in all, given the fact that the analysis of cellular lipids has not yet been addressed, here we propose a novel approach to identify lipids uniquely derived from cells and the biological pathways in which they are involved. The underlying principle of this approach is that those lipids that derive from cells must increase in abundance when analyzing samples with an increasing number of cells, while lipids derived from other origin should not follow this pattern. So, using a cell curve constructed with several cell counts as a base for subsequent correlation analyses to identify the mentioned pattern, we were able to identify signals whose abundance increased proportionally with the number of cells, relating them to intrinsic cellular processes. This proof of concept was carried out using T cells (CD3^+^) isolated from a human donor. T cells are relatively small, but present a highly active metabolism (10). The approach was then applied to study the response of human microvascular dermal endothelial cells (HMVEC-d) stimulated with serum from patients with anaphylaxis both at the acute stage of the reaction and after 14 days, at baseline (**Figure 1**). The application of this correlation-based approach allowed us to identify cellular lipids, such as sphingomyelins and phospholipids, as the main lipid classes that change in HMVEC-d upon contact with serum from anaphylactic reactions. Overall, this novel methodology enabled the selective identification of cell-derived chemical signals and their biological interpretation across different cellular models in untargeted lipidomics studies.

## Materials and methods

### Sample collection

#### CD3^+^ cell isolation and cell count curve construction

To explore the relationship between the number of cells and lipid abundances measured by untargeted analyses, CD3^+^ cells were employed. Briefly, peripheral blood mononuclear cells (PBMCs) of a single donor were isolated from a leukocyte reduction system chamber (LRSC), a discard product from plateletpheresis procedures containing blood enriched in PBMCs (11), using a Ficoll-Paque PLUS (Cytiva; catalog number: 11768538; Cytiva, Uppsala, Sweden) gradient. PBMCs were preserved in liquid nitrogen until use.

The day of the experiment, PBMCs were thawed and incubated with 30 mL of Gibco™ media RPMI 1640 GlutaMAX™ supplement (Thermo Fisher Scientific; catalog number: 11569726; Waltham, MA, USA) containing 10% of heat inactivated Gibco™ FBS certified (Thermo Fisher Scientific; catalog number: 15808947; Waltham, MA, USA) at 37°C for 2h. Then, PBMCs were centrifuged at 1200 x *g*, and CD3^+^ cells obtained using a MACS cell separation kit with magnetic Microbeads (Miltenyi Biotec; catalog number: 130-050-101; Bergisch Gladbach, Germany) following the manufacturer’s instructions. After isolation, cells were counted using an automatic cell counter Mythic 18 (Orphée SA; PlanlesOuates, Geneva, Switzerland), and CD3^+^ cells were divided into 6 tubes with increasing cell counts: 250,000 (250k) cells, 500k cells, 1,250,000 (1.25M) cells, 2.5M cells, 3.75M cells, and 5M cells. Then, each tube was centrifuged at 1200 x *g* for 10 min and supernatants were discarded. Cell pellets were resuspended in 500 µL of cold, LC-MS grade methanol (MeOH) (Fisher Chemical, Thermo Fisher Scientific; catalog number: A456212; Waltham, MA, USA) to quench cell metabolism and distributed in 5 biological replicates, each containing 100 µL of the cell suspension, thus defining each experimental cell count (total cells per cell count: 50k, 100k, 250k, 500k, 750k, and 1M). Cells in MeOH were kept at -80°C until further analysis.

#### Serum-treated HMVEC-d endothelial system

##### Recruitment of patients with anaphylaxis

A total of 24 patients with immediate drug hypersensitivity reactions (iDHR) suffering anaphylaxis under drug provocation test (DPT) were recruited at Hospital Central de la Cruz Roja San José y Santa Adela. Allergist confirmed the anaphylaxis diagnosis using the criteria from the National Institute of Allergy and Infectious Diseases and the Food Allergy and Anaphylaxis Network (12). For each patient, data on gender, age, reaction trigger, treatment timing, symptoms, and reaction severity (based on Brown’s criteria) (13) were recorded. Briefly, grade 1 reactions were those with mild symptoms including skin and subcutaneous tissues only; grade 2 reactions were characterized by moderate features suggesting respiratory, cardiovascular, or gastrointestinal involvement; and grade 3 reactions were those presenting hypoxia, hypotension, or neurologic compromise.

Patients were classified into three groups according to the mechanism triggering the anaphylaxis, based on clinical presentation, eliciting drug and results of allergological evaluation following clinical practice guidelines: **1)** IgE-mediated iDHR (IgE group), including those with immediate reactions to β-lactams or proton pump inhibitors (positive DPT) and evidence of an IgE mechanism confirmed by positive results for skin test (ST) and/or sIgE and/or basophil activation test (BAT) (*n* = 8) (14, 15); **2)** COX-1 inhibitors-mediated iDHR (COX group), including those with previous iDHR to different nonsteroidal anti-inflammatory drugs (NSAIDs) and positive DPT to acetylsalicylic acid, confirming COX-1 intolerance (*n* = 8) (16); and **3)** MRGPRX2 receptor-mediated iDHR (MRGPRX2 group), including those exhibiting immediate reactions to fluoroquinolones or glycopeptides (positive DPT) and excluding IgE-mediated mechanism through allergological investigation, including negative ST results (*n* = 8) (17).

##### Anaphylaxis patient serum collection

Fresh blood samples from patients were collected at acute stage (immediately after clinical appearance of symptoms) and after recovery, having passed at least 14 days (baseline stage), during a scheduled follow-up visit at the allergological department. All patients recovered and did not suffer other anaphylactic reactions between the acute and baseline stages. Sera from acute and baseline stages were processed using BD Vacutainer tubes containing a separator gel. Peripheral blood samples were centrifuged at 1,200 x *g* for 10 minutes at 4°C. The resulting serum was aliquoted into 0.5 mL portions and stored at −80°C until experimental use, and serum tryptase was measured in each condition. This study adhered to the principles of the Declaration of Helsinki and received approval from the Ethics Committee of the hospital (HULP, PI-2712). All participants provided written informed consent before inclusion.

##### Endothelial cell system and cell count curve preparation

HMVEC-d were acquired from Lonza (catalog number: CC-2543) and maintained in EGM-2MV BulletKit medium. 50k cells/well were plated and incubated with sera samples the following day (18). This sera/endothelial *in vitro* system was kept for 1 hour with 1 mL of a 1:1 mixture of the collected serum: EGM-2MV media, after which supernatant was removed, and cells were washed with PBS and resuspended in 100 µL of MeOH to quench their metabolism.

Additionally, a cellular curve was prepared following the same strategy as for CD3^+^ cells. HMVEC-d were plated at 25k, 50k, 75k, and 100k cells/well. Next day, cells were washed with PBS and resuspended in 100 µL MeOH MS grade for the lipidomics analysis. Samples were stored at -80°C until further analysis.

### Lipidomics analysis

#### Reagents for lipidomics

Reverse‐osmosed ultrapure water, used to prepare all the aqueous solutions, was obtained from a Milli‐Qplus185 system (Millipore, Billerica, MA, USA). LC-MS grade MeOH (catalog number: A456212), acetonitrile (ACN, catalog number: A955212), and isopropanol (IPA, catalog number: A461-212) were obtained from Fisher Chemical (Thermofisher Scientific, Waltham, MA, USA). HPLC-grade methyl *tert−*butyl ether (MTBE, catalog number: 34875-1L), and ammonium fluoride (NH_4_F) (ACS reagent, ≥ 98%; catalog number: 216011-100G) were purchased from Sigma‐Aldrich (Merck KGaA, Steinheim, Germany). Analytical grade ammonia solution (28%, GPR RECTAPUR^®^, catalog number: 21182.294), formic acid (HiPerSolv CHROMANORM^®^, catalog number: 84865.290) and acetic acid glacial (AnalaR^®^ NORMAPUR^®^, catalog number: 20099.290) were obtained from VWR Chemicals (VWR International, Radnor, PA, USA). LightSPLASH LIPIDOMIX^®^ (SPLASH^®^ LIPIDOMIX^®^ Mass Spec Standard, catalog number: A83707, Avanti Polar Lipids, Inc., AL, USA) was used as the internal standard (ISTD).

#### Lipid extraction

##### CD3^+^ cells

For lipid extraction, the 100 µL cell suspension aliquots were mixed with 25 µL of MTBE at room temperature to reach a proportion of 4:1 MeOH: MTBE. Then, samples were thoroughly vortex-mixed for 10 seconds, sonicated 3 times for 5 min, and centrifuged at 15°C, 16,000 x *g* for 20 min. Supernatants were then transferred to a LC vial. Furthermore, both an instrumental blank (composed of MeOH) and a cell culture media sample (consisting of RPMI containing 10% FBS, both from the same batch used for cell incubation) were included. A quality control (QC) sample was prepared by pooling equal volumes of all experimental samples after lipid extraction.

##### HMVEC-d

For each cell suspension in MeOH, 20 µL of light SPLASH Lipidomics ISTD were added. Then, two consecutive extractions were performed. In the first extraction, samples were thoroughly vortex-mixed for 1 minute, then ultrasonicated in a bath sonicator for 10 min and centrifuged at 16,000 x *g* for 20 min at 4°C. Then, supernatants were transferred to an LC vial. For the second extraction, 50 µL of cold MeOH were added to the pellet, and the steps of vortex, ultrasonication and centrifugation were repeated. The resultant supernatant was mixed with the previous one for each sample. For QC sample, 10 µL and 5 µL from the 1^st^ and 2^nd^ extraction, respectively, from each sample were mixed into a LC vial.

For both cellular models, a QC sample was measured repeatedly throughout the analysis to ensure system stability and reproducibility.

#### Instrumental analysis

##### CD3^+^ cells

Lipid extracts were measured by untargeted lipidomic analysis in an Agilent 1290 Infinity II ultra-high performance liquid chromatography (HPLC) system coupled to an Agilent 6545 quadrupole-time-of-flight mass spectrometer (QToF-MS) (Agilent Technologies; Santa Clara, CA, USA) in both positive and negative electrospray ionization (ESI+ and ESI-, respectively) modes, as previously described (19, 20).

Chromatographic analysis was carried out using an InfinityLab Poroshell 120 Å EC-C8 column (2.1 × 150 mm, 2.7 μm) (Agilent Technologies; catalog number: 693775-906; Agilent Technologies, Santa Clara, CA, USA) maintained at 60°C. Flow rate was set at 0.5 mL/min, and the mobile phases consisted of (A) H_2_O and (B) MeOH + 15% isopropyl alcohol (IPA), both containing 10 mM ammonium formate (NH_4_HCO_2_) as a modifier. For the elution gradient, the initial conditions were set at 75% B, which increased to reach 96% B at 23 min and then was maintained until 31 min. Then, gradient rapidly increased to reach 100% B at 31.5 min and was maintained until 32.5 min before returning to the initial conditions at 33 min. The conditions were maintained to re-equilibrate the column until 40 min. Injection volume was set at 2 µL for both ESI+ and ESI-.

The MS conditions were as follows: capillary voltage was 3,500 V, drying gas flow rate was 11 L/min for ESI+ and 13 L/min for ESI- at 290°C, and gas nebulizer energy was 40 psi. Sheath gas temperature was 370°C at a flow rate of 11 mL/min. Fragmentor voltage was 175 V, skimmer and octopole radio frequency voltage (OCT RF Vpp) were set at 65 V and 750 V, respectively. Data were collected in centroid mode at a scan rate of 1.0 Hz. The MS detection window was performed in a full scan from *m/z* 50 to 1,700 for both ESI modes. During the analyses, two reference masses were used: *m*/*z* 121.0509 [purine, (C_5_H_4_N_4_+H)^+^] and *m*/*z* 922.0098 [HP-0921, (C_18_H_18_O_6_N_3_P_3_F_24_+H)^+^] in ESI+ mode and *m*/*z* 112.9855 [TFANH_4_, (C_2_H_4_O_2_NF_3_-NH_4_)^−^] and *m*/*z* 966.0007 [HP-0921, (C_18_H_18_O_6_N_3_P_3_F_24_+FA-H)^−^] in ESI- mode. The reference masses were continuously infused at 1.0 mL/min into the system, enabling constant mass correction. Cell counts suspension aliquots were measured in increasing cell count order (50k, 100k, 250k, 500k, 750k, and 1M) analyzing each biological replicate (*n* = 5 per cell count) in a random order.

##### HMVEC-d

For sample analysis, the same instrument was used as for the CD3^+^ analysis using a different method. For the chromatographic analysis, an InfinityLab Poroshell 120 Å EC-C18 column (3.0 × 150 mm, 2.7 μm) maintained at 50°C was used. Flow rate was set at 0.6 mL/min, and the mobile phases consisted of (A) a 9:1 H_2_O:MeOH mixture, and (B) ACN: MeOH: IPA in a 2:3:5 proportion, both containing 10 mM ammonium acetate (NH_4_CH_3_CO_2_) and 0.2 mM NH_4_F as modifiers. For the elution gradient, the initial conditions were set at 70% B, which increased to reach 96% B from 1 min to 3.5 min. This was maintained until 10 min; then, rapidly increased to 100% B at 11 min and maintained until 17 min before rapidly returning to the initial conditions at 17.1 min. Initial conditions were maintained to re-equilibrate the column until 19 min. In this case, cell suspension aliquots of increasing cell counts were analyzed by triplicate at the beginning, middle and end of the worklist, generating three technical replicates. In addition, the QC sample was analyzed at the beginning and end of the worklist and at consecutive intervals, every 5–6 samples. The experimental samples were analyzed randomly across the worklist.

Data was acquired in ESI+ and ESI- modes. The injection volume was set at 2 and 4 µL for ESI+ and ESI-, respectively. Capillary voltage was set at 3,500 V and 3,000 V for ESI+ and ESI-, respectively. The drying gas flow rate was 10 L/min at 200°C, and the gas nebulizer energy was 40 psi. Sheath gas temperature was 300°C at a flow rate of 12 mL/min. Fragmentor voltage was 175 V, skimmer and octopole radio frequency voltage (OCT RF Vpp) were set at 65 V and 750 V, respectively. Data were collected in centroid mode at a scan rate of 3.0 Hz. The MS detection was performed in a full scan from 50 to 1,800 *m/z* for both ESI modes. The reference masses were the same as previous analysis.

For identification purposes, ten iterative-MS/MS runs were performed for both ion modes at the end of the analytical run. They were operated with an MS and MS/MS scan rates of 3 spectra/s, 40–1,800 *m/z* mass window, a narrow (∼ 1.3 amu) MS/MS isolation width, 3 precursors per cycle, 5,000 counts, and 0.001% of MS/MS threshold. Five iterative-MS/MS runs were set with a collision energy of 20 eV, and the subsequent five runs were performed at 40 eV. Reference masses and contaminants detected in blank samples were excluded from the analysis to avoid inclusion in the iterative-MS/MS.

#### Quality assessment

Acquired raw data obtained by the HPLC-QTOF/MS were processed to provide structured raw data in an appropriate format for analysis by using Batch Recursive Feature Extraction (BRFE) tool included in MassHunter Profinder software (B.10.00; SP3, Agilent Technologies^®^). The BRFE algorithm creates a list of neutral masses and retention times, associated with the abundance of the possible components representing the full TOF masses from the spectral data. Each component is the sum of co-eluting ions that are related by charge-state envelope, isotopic distribution, and/or the presence of different adducts and dimers. In order to find co-eluting adducts of the same feature, the following adducts were selected: for the CD3^+^ cell model, +H, +Na, +K, and +NH_4_ for ESI+ mode and -H, +HCOO^-^, +Cl^-^ for ESI- mode; while for HMVEC-d, we selected the adducts +H^+^, +Na^+^, +K^+^, and +NH_4_^+^, and +C_2_H_7_N_2_^+^ for ESI+ mode and -H^+^, +CH_3_COO^-^, and +Cl^-^ for ESI- mode. In both, neutral loss of water was also considered. Data is available at the Metabolomics Workbench database (21).

Obtained data were then processed in R software v.4.4.0, mainly withdplyr R package (22). First, when analyzing CD3^+^ samples, as the worklist in ESI+ mode stopped during analysis, data were normalized using inter-batch correction from QC medians (23). To ensure the reliability of further analyses, a quality assurance (QA) stage was included in the workflow. This served to filter out low-abundance signals and potential background noise and was applied in both ESI modes and for both cellular models. Thus, only chemical signals that met QA criteria, namely, those where **1)** the average abundance of the biological replicates in each point of the cell curve (and also in each experimental condition for the HMVEC-d model) was greater than the average abundance from the blanks; and **2)** detection was possible in at least 75% of QC samples, were kept for further analyses. Chemical signals that did not meet both criteria were excluded. Then, missing values were imputed using the *k*-nearest neighbors (k-NN) algorithm (VIM and laeken R packages) (24, 25), an optimal approach for missing values imputation in metabolomics (26, 27). After missing value imputation, chemical signals with a coefficient of variation (CV) higher than 30% were discarded. Data that passed QA were tested using principal component analysis (PCA) and hierarchical clustering (HC) based on Euclidean distance and complete linkage method [stats R package (28)]. Abundances were log_10_-transformed and range-scaled before analysis and graphic representation. After PCA, only ESI– data of CD3^+^ cells was normalized using QC-SVRC algorithm (29, 30) to correct for analytical drift.

#### Cell curve correlations

To ensure that only chemical signals derived from cells were included in the statistical analyses, we propose the use of a cell curve alongside experimental samples of a traditional cell lipidomics workflow to enable distinguishing cellular lipids from chemical signals of other sources. Thus, Spearman correlation tests were performed between the number of cells and chemical signals’ abundance [stats R package (28)] in the different existing cell count intervals (e.g., 50k-1M cells for CD3^+^ cells and 25k-100k for HMVEC-d). Only significant (*p*-value < 0.05) and positively correlated (*ρ* ≥ 0.7, associated to strong correlations (31)) chemical signals were selected. This way, we could ensure that only signals showing a clear and biologically meaningful increase with cell number are retained, reducing the likelihood of false positives while maintaining adequate sensitivity. Correlation *p*-values were not adjusted for multiple testing, and robustness to alternative correlation thresholds was not formally assessed, as the analysis was intended as a conservative filtering step rather than inferential testing.

#### CD3^+^ cells normalization approaches

Three normalization strategies were applied to CD3^+^ cell–associated chemical signals using R (primarily the *dplyr* and *tibble* packages), and results were compared between the standard and the correlation-based approaches: **1)** chemical signal abundances were normalized by dividing each signal by the corresponding CD3^+^ cell count, **2)** abundances were normalized by dividing each chemical signal by the total useful signal (TUS), and **3)** abundances were normalized by dividing the abundance of each chemical signal by the median abundance of that same chemical signal across all samples.

### Statistical analysis

To test the strength of this proposed methodology, statistical analysis for the HMVEC-d model was carried out using all chemical signals after QA or keeping only the ones that positively correlated with the increasing cell number. Statistical analysis was performed on experimental HMVEC-d samples to obtain differences after treatment with serum from different stages (acute vs. baseline), different groups (IgE, COX, and MRGPRX2) or the interactions between these two factors. Distribution normality and homocedasticity were tested with Shapiro-Wilk Normality Test and Levene Test, respectively, for each chemical signal using *rstatix* R package (32), leading to the use of either two-way mixed ANOVA or Aligned-Rank Transform (ART) ANOVA (*emmeans*, *ARTool*, and *afex* R packages (33–37)). These two statistical tests treated time (acute vs. baseline) as a within-subjects repeated-measures factor, with patient identity accounting for the paired nature of the data, and anaphylaxis mechanism–based groups (IgE, COX, and MRGPRX2) as a between-subjects factor. Two-way mixed ANOVA was used for chemical signals that met parametric assumptions (normality of residuals and homogeneity of variance). Those chemical signals that did not meet these assumptions were analyzed using ART ANOVA, a non-parametric alternative that allows factorial designs while retaining the ability to test interactions between factors (34). Only significant (*p*-value < 0.05 and *p*-adjusted value < 0.2, a threshold used for exploratory studies was calculated using the Benjamini-Hochberg correction method) chemical signals were selected for further analyses (38, 39).

### Lipid annotation

For the annotation of the chemical signals for the CD3^+^ model, representative lipids were identified based on the *m/z* accuracy, RT, and an in-house library (19). In the case of HMVEC-d model, raw LC-MS/MS data were first imported into the Lipid Annotator software (Agilent) to create a fragmentation-based (MS/MS) library containing the *m/z* and matching retention time (RT) of all precursors recognized as lipids. All ion species were selected for ESI+ ([M+H]^+^, [M+Na]^+^, and [M+NH4]^+^) and for ESI– ([M-H]^-^, [M+HCOO]^-^, and [M+CH3COO]^-^). The Q-score threshold was set at ≥ 30, all lipid classes were selected, mass deviation threshold was established as ≤ 20 ppm, fragment score threshold was fixed as ≥ 30, and total score was set at ≥ 60.

Second, raw LC-MS/MS data were imported into the open-source MS-DIAL software (40) in order to achieve additional lipid annotations and confirm previous ones. The same ion species used in Lipid Annotator were used for MS-DIAL and, additionally, the neutral loss of water was considered for both ion modes.

Finally, a manual MS/MS spectral inspection was carried out using the Agilent MassHunter Qualitative software (version 10.0) to confirm the previously obtained annotations. The fragmentation patterns at 20 and 40 eV were compared to online available MS/MS data bases from MassBank (https://massbank.eu/MassBank/), PubChem (https://pubchem.ncbi.nlm.nih.gov/), LIPID MAPS (https://www.lipidmaps.org/) and the Human Metabolome Database (https://hmdb.ca/) (41–44). To ensure a correct annotation, co-elution of possible adducts for the specific lipid subclass was also analyzed (19). For further analysis, only lipids that were manually annotated and/or assigned to the same annotation by both software programs (Lipid Annotator and MS-DIAL) were considered.

### Pathway enrichment analysis

Lipid HMDB IDs were obtained with MetaboAnalyst 6.0 (45). IMPaLA (46) pathway over representation analysis was performed with those identified lipids with an available HMDB ID (n = 44). A *p*-adjusted value threshold of 0.05, calculated using the Benjamini-Hochberg (BH) correction method, was applied to identify significantly enriched pathways.

### Data visualization

Data visualization was performed using the following R packages: *colorRamp2*, *GGally*, *ggpattern*, *ggpbur*, *ggplot2* and *network* (47–53). Additionally, lipid trajectories were plotted using GraphPad Prism v 10.0 (GraphPad Software, Boston, MA, USA).

The lipid metabolic network was generated using LINEX² (54) (native network option), where nodes represent lipid species (including both sum and molecular species) and edges represent biochemical reaction types inferred from known enzymatic transformations (based on Reactome and Rhea) (55, 56). Additionally, node sizes correspond to the fold-change in abundance (Acute/Basal). It is important to note that sphingosine, fatty acids (FA), and acyl carnitines (CAR) were not included in the network, as the LINEX² framework does not account for these lipid classes.

## Results

### Study of different cell counts lead to a new strategy to ensure the selection of cell derived signals

The analysis of increasing cell counts of CD3^+^ cells by untargeted lipidomics was performed following the described analytical pipeline (**Figure 1**). This unique model served as a controlled proof of concept to study the response of the lipid signals along with the increasing cell number, as it contains 6 cell counts with 5 biological replicates in each, as well as the cell media. Compared to traditional lipidomics workflow, the analytical pipeline proposed includes the application of correlation analysis after QA. Interestingly, during the QA procedure, we observed that from the 1,262 and 342 chemical signals obtained for ESI+ and ESI-, respectively, after data filtering most of them did not present many missing values (97% and 73%, respectively, **Supplementary Figures 1A, B**). When focusing on cell counts, the highest percentage of missing values did not exceed 0.5% in ESI+ and 15% in ESI- (**Supplementary Figures 1C, D**). After finalizing QA procedure, we obtained a total of 1087 and 249 chemical signals (*k*) for ESI+ and ESI-, respectively. Data quality was assessed by clustering of QC samples in PCA models in both ionization modes (**Supplementary Figure 2**). In addition, both PCA models showed a slight pattern associated with the increasing counts of CD3^+^ cells: bottom-to-top, for ESI+ mode, and backwards in the case of ESI- mode. Interestingly, these PCA plots for CD3^+^ cells also showed that cell media injections were extremely different compared to the different cell counts, suggesting that this cannot be used to compare with the lipidic profile of the cells. Chemical signals coming from the cell media could mask the lipid signals coming from the cells.

**Figure 2:**
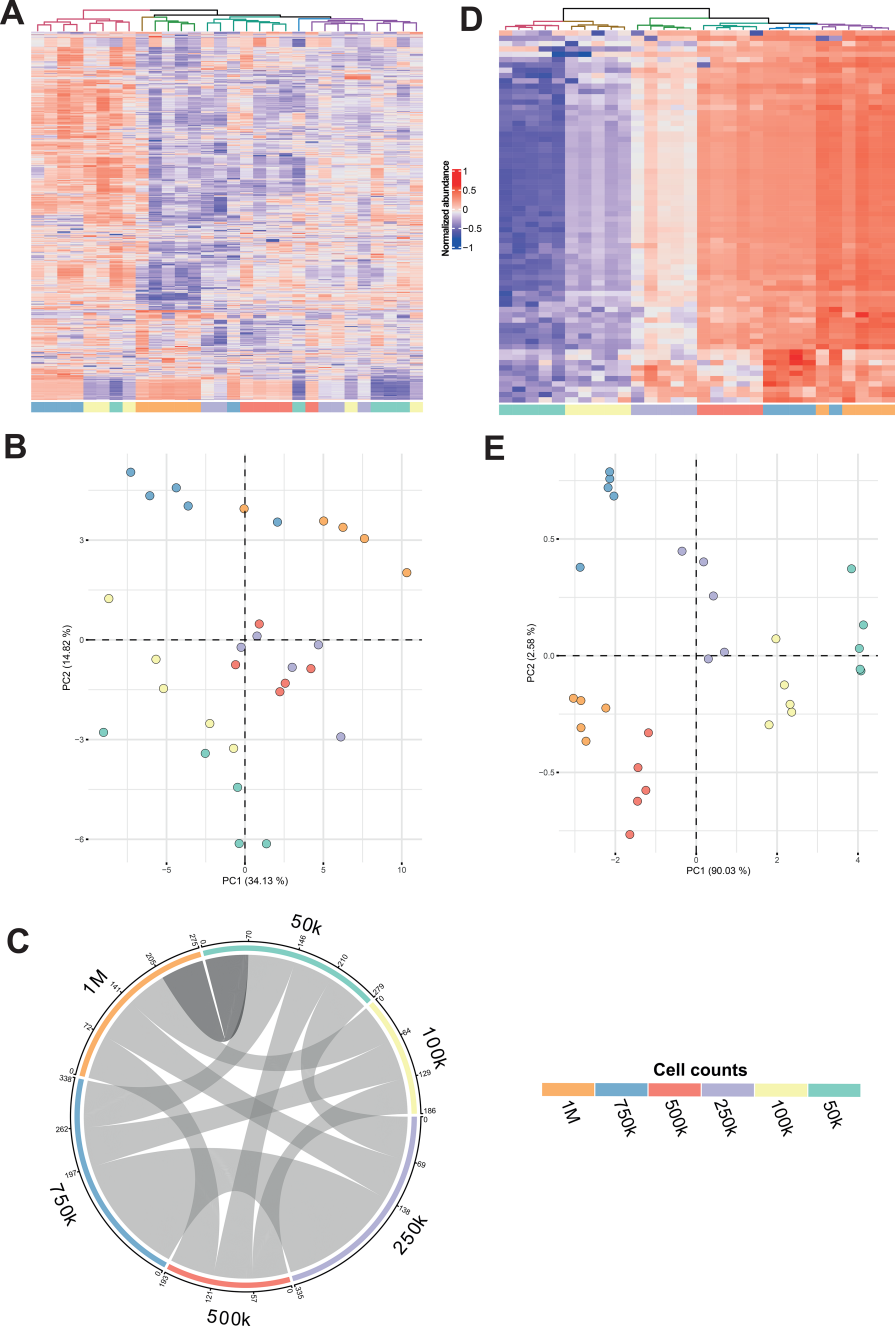
Correlated chemical signals allow an accurate grouping of samples by cell count in CD3^+^ cells model for ESI+ mode. **(A)** Heatmap with hierarchical clustering (HC) of all detected chemical signals (*k* = 1087). **(B)** Principal Component Analysis (PCA) model generated using all detected chemical signals (*k* = 1087). **(C)** Chord diagram showing the number of significant (*p* < 0.05) and positive (*ρ* > 0.7) correlations between chemical signal abundances and cell counts across defined cell count intervals. The connecting link thickness represents the number of correlated chemical signals (the greater the number, the thicker the line) in the interval defined by the 2 connected cell counts. Each cell count considered for the correlation analyses and with at least 1 significant correlation is represented. Darkest gray link highlights the widest cell count interval (50k-1M), which is selected for further analyses. **(D)** Heatmap with HC of chemical signals that were significantly and positively correlated with the cell count using data from the 50k-to-1M interval (*k* = 70). **(E)** PCA model of chemical signals that were significantly positively correlated with the cell count using data from the 50k-to-1M interval (*k* = 70). For the CD3^+^ cohort, 5 biological replicates at 6 increasing cell counts were analyzed individually.

In addition, to explore in more detail the lipidomic profiles of the CD3^+^ cells, the abundances of the individual chemical signals were visualized using heatmaps with HC and PCA plots for ESI+ and ESI- (**Figures 2A, B**, **Supplementary Figures 3A, B**, respectively). In the case of ESI+ mode, the heatmap and the PCA plot showed specific clusters, where samples were mainly grouped by similar cell count (i.e., 750k with 1M, 250k with 500k, and 50k with 100k). Moreover, the explained variance in this PCA was under 50%, what points to the presence of signals unrelated to cells, highlighting the importance of selecting only cell-derived chemical signals.

To select those chemical signals whose abundance increases with the CD3^+^ cell counts (i.e., cell-derived chemical signals), Spearman correlation analyses between cell counts and abundances of chemical signals were performed. For this, and considering that the smallest cell count we had was 20 times below the highest cell count [which contained the recommended amount of cells from literature for cell lipidomics (4–6)], we performed correlation analyses using all possible cell count intervals that contained at least three cell counts (e.g., 50k-to-1M or 250k-to-750k) to ensure meaningful statistical interpretation. Thus, intervals with only two cells counts (e.g., 50k-to-100k) were not considered. Results were represented in a chord diagram (**Figure 2C**, **Supplementary Table 1**). We observed that the highest number of correlated chemical signals was found for the 250k-to-750k CD3^+^ cell count interval for both polarities (*k* = 197 for ESI+ and *k* = 14 for ESI-, **Supplementary Figure 3C**, **Supplementary Table 2**). Nonetheless, we decided to continue our analysis with the widest cell count interval (i.e., with 50k-to-1M), and only considering those chemical signals that significantly and positively correlated in this interval (*k* = 70 for ESI+ and *k* = 11 for ESI-). Again, heatmaps with HC and PCA models were carried out for these selected chemical signals. For ESI+, the heatmap with HC revealed an accurate grouping according to cell count (**Figure 2D**), with all samples clustering accordingly except for one 750k replica. In addition, we observed that the color gradient of the abundances varied consistently with cell count. In the PCA plot (**Figure 2E**), there was a clear distribution of samples according to their cell count, with the first component explaining more than 90% of variance among samples, and samples being clustered from 50k to 1M cells, arranged right-to-left. Although similar results were observed for ESI- (**Supplementary Figures 3D, E**), the low number of correlated chemical signals made the separation and grouping less accurate. Nonetheless, samples with lower cell count (50k and 100k) mostly clustered on the right, samples with the highest cell counts (750k and 1M) mostly clustered to the left, and intermediate samples (250k and 500k) were scattered among the middle part of the PCA plot (**Supplementary Figure 3E**). To demonstrate the increase of the chemical signals along the cell counts, representative lipids were identified from those which positively correlated (*ρ* ≥ 0.7 and *p*-value < 0.05). These included mainly glycerophospholipids including glycerophosphocholines (PC), sphingomyelins (SM) and plasmalogens (PC(O-/P-)), **Supplementary Figure 4**. As it is observed, a clear linear tendency was followed along the cell curve showing the power of this methodology to select only those lipids coming from the cells.

In addition, the controlled experiment allowed the study of the normalization strategies (57) most used in cell lipidomics (and metabolomics). These include normalizing each chemical signal dividing by the median, dividing by the cell counts, and dividing by the TUS. The analysis of data acquired in ESI+ and ESI- after normalization using PCA models after the correlation-based analysis are found in **Supplementary Figures 5**, **6**, respectively. These PCA models showed similar results for all normalization strategies after the correlation-based analysis, obtaining the clustering of the cell counts. However, small differences were noted, e.g., TUS normalization consistently provided the best performance, as evidenced by tighter co-clustering of samples. Median normalization ranked second, showing intermediate clustering tightness, whereas cell count normalization resulted in higher variability. Importantly, all normalization strategies benefited from prior filtering of non-correlated signals, which led to a substantial reduction in noise and improved global sample clustering (**Supplementary Figures 5**, **6**). Furthermore, we also analysed the trajectories of the representative lipids to study the efect of these normalization strategies (**Supplementary Figure 7**). As observed, similar findings were obtained between normalization procedures, showing that most of the trajectories followed a flat line among the cell counts, indicating a good performance of the normalization procedures. Altogether, here we show that HC and PCA models conveyed an improved sample grouping according to cell counts after selection of correlated chemical signals (i.e., those derived from cell metabolism), pointing toward the relevance of this methodological approach to filter out confounding signals. Moreover, when comparing the three normalization strategies, all normalizations provide good performance only after correlation-based filtering. These observations underscore the importance of applying the normalization method only in combination with correlation-based signal filtering to obtain reliable and interpretable results.

### The cell curve and the correlation strategy are applicable for other cell types in an experimental setting

**Figure 3:**
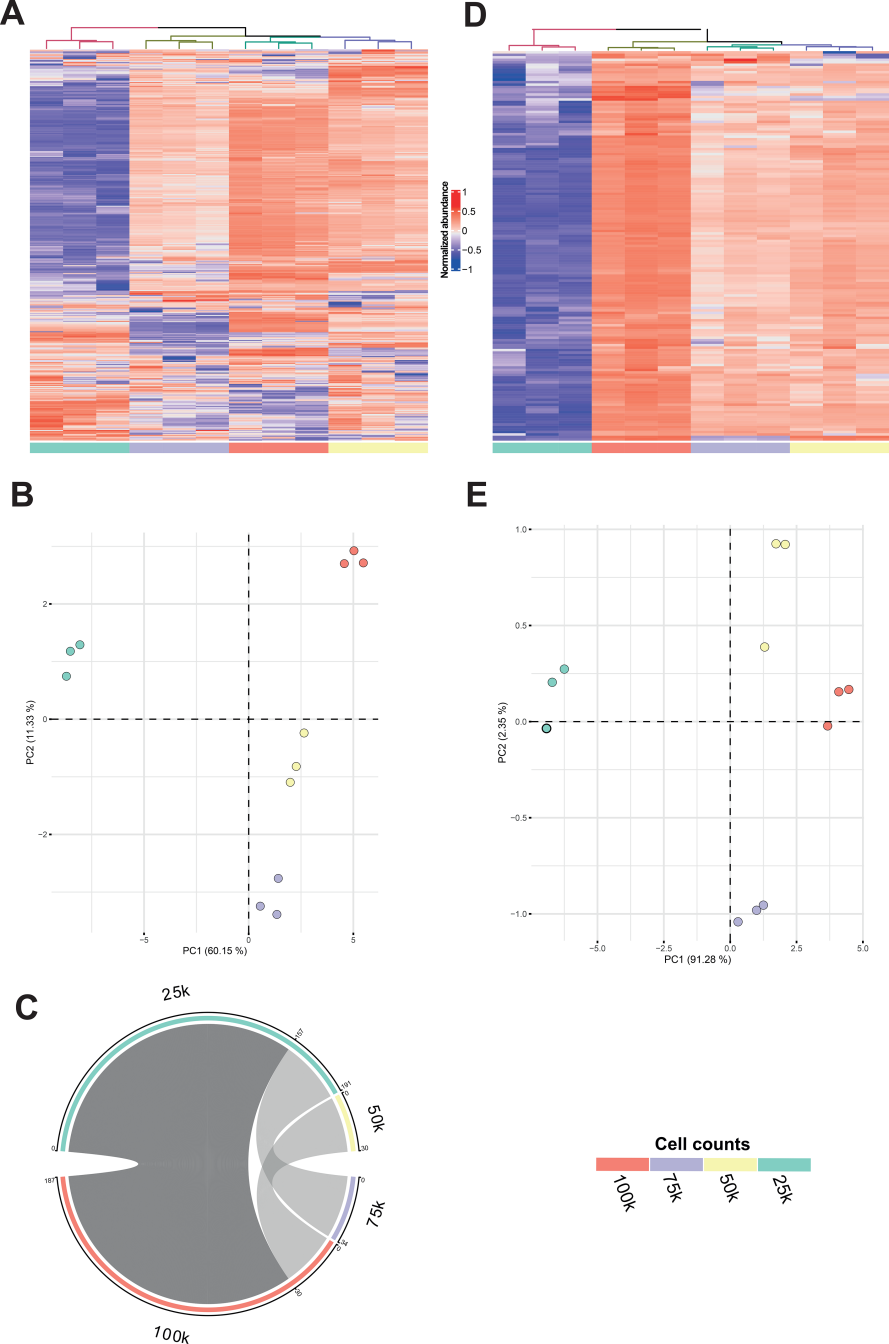
Correlated chemical signals allow an accurate grouping of samples by cell count in HMVEC-d model for ESI+ mode. **(A)** Heatmap with hierarchical clustering (HC) of all detected chemical signals (*k* = 408). **(B)** Principal Component Analysis (PCA) model generated using all detected chemical signals (*k* = 408). **(C)** Chord diagram showing the number of significant (*p* < 0.05) and positive (*ρ* > 0.7) correlations between chemical signal abundances and cell counts across defined cell count intervals. The connecting link thickness represents the number of correlated chemical signals (the greater the number, the thicker the line) in the interval defined by the 2 connected cell counts. Each cell count considered for the correlation analyses and with at least 1 significant correlation is represented. Darkest gray link highlights the widest cell count interval (25k-100k), which is selected for further analyses. **(D)** Heatmap with HCA of chemical signals that were significantly and positively correlated with the cell count using data from the 25k-to-100k interval (*k* = 157). **(E)** PCA model of chemical signals that were significantly positively correlated with the cell count using data from the 25k-to-100k interval (*k* = 157). For the HMVEC-d cohort, the cell curve consisted of 4 increasing cell counts injected by triplicate.

Once the methodological approach was established and the proof-of-concept was explored, we applied it to HMVEC-d model. In this case, cell curve was characterized by 4 cell counts made up of 3 technical replicates. The cell curve was analyzed together with experimental samples in order to determine the lipid changes induced by the sera of anaphylactic patients in the HMVEC-d. In this way, we followed the traditional cell lipidomics approach (58) adding the novelty of the cell curve which would allow us to select lipids from the cells by applying correlation analysis. Following the same pipeline of analysis as for CD3^+^ cells (**Figure 1**), we observed that during the QA procedure, from the 478 and 690 chemical signals obtained for ESI+ and ESI-, respectively, after data filtering, most of them did not present many missing values (84% and 67%, respectively, **Supplementary Figures 8A, B**). When focusing on experimental cell samples, a maximum of approximately 6% and 14% missing values were found in ESI+ and ESI-, respectively (**Supplementary Figures 8C, D**). After applying the QA procedure, we obtained a total of 408 and 580 chemical signals in ESI+ and ESI-, respectively. Quality of the data was assessed by QC clustering on the PCA models for both polarities (**Supplementary Figure 9**). We noted a pattern of cell counts clustering ordered by cell number in both HC and PCA plots (**Figures 3A, B**, **Supplementary Figures 10A, B**). Despite this pattern, the variance explained by PCA models was only around 60% for both polarities. Again, correlation analyses were carried out for each cell count interval and represented using a chord diagram (**Figure 3C**, **Supplementary Figure 10C**, **Supplementary Tables 3**, **4**). We observed that the highest number of positive correlations was found in the interval that included all cell counts (i.e., 25k-to-100k), obtaining 157 and 276 chemical signals for ESI+ and ESI-, respectively. After selecting only positively correlated chemical signals, both the HC and PCA models improved the clustering of the cell counts (**Figures 3D, E**, **Supplementary Figures 10D, E**). In the PCA models, the first component explained around 90% of the variance across samples in both polarities (**Figure 3E**, **Supplementary Figure 10E**). Complementarily, for the analysis of the HMVEC-d cohort, LightSPLASH LIPIDOMIX^®^ was used to additionally explore the performance of the analysis. From this mix of 13 standards, we considered only the lipids with non-endogenous fatty acyl chains, including PC (15:0_18:1), glycerophosphoethanolamine (PE) (15:0_18:1), glycerophosphoinositol (PI) (15:0_18:1), diacylglycerol (DG) (15:0_18:1), and ceramide (Cer) (d18:1_15:0) (**Supplementary Figure 11**). The %CV of these ISTD in QC injections ranged from 1.7% to 7.3%. In addition, the cell counts showed a %CV < 30%, indicating that ISTD did not seem to be affected by differences in increasing cell numbers. These findings confirmed the quality of the analysis and suggested that those ISTD could be used for monitoring the analytical response or been used in instrumental normalization procedures (**Supplementary Table 5**).

Overall, these results demonstrate that this new strategy for untargeted cell lipidomics analysis is also applicable to other cell types beyond immune cells (including using different cell numbers and sizes), and that the increasing number of cell count do not seem to affect the ISTD response.

### HMVEC-d show an altered lipidome profile in response to anaphylaxis

As samples from the HMVEC-d cohort were analyzed including a cell curve, we were able to apply the statistical analysis to the experimental samples from two data sets: 1) all data that passed the QA procedure (*k* = 408 and 580 for ESI+ and ESI-, respectively) as it would be in a lipidomics analysis (standard approach), and 2) chemical signals that correlated positively with the increasing cell counts (*k* = 157 and 276 for ESI+ and ESI-, respectively). The latter is the proposed novel methodology.

In terms of the clinical and demographic characteristics of the patients, 66% of the subjects were female, with an average age of 42. The majority (84%) presented with an anaphylactic reaction of grade 2 following Brown's classification (13) (**Table 1**). There were 8 patients (33%) for each iDHR mechanism, classified as IgE, COX, and MRGPRX2. The majority of the patients (96%) presented cutaneous manifestations, followed by respiratory complications (66%) and mucosal affections (54%). Regarding their treatment, all patients received at least H1 receptor antagonists and corticosteroids. Characteristics of the patients classified by the severity of the anaphylaxis reaction is summarized in **Table 1**. Additionally, the clinical description of each individual patient is presented in **Supplementary Table 6**.

**Table 1:** Clinical characteristics of anaphylactic patients.

Characteristics	Patients			
	Total	Grade 1	Grade 2	Grade 3
N° of subjects	24	4	19	1
Sex (F)	66%	50%	84%	100%
Age (years) mean ± SEM	42.1 ± 2.98	53.8 ± 10.9	39.4 ± 2.85	47 ± 0
Mechanism				
*IgE*	33%	0%	88%	100%
*COX*	33%	0%	100%	0%
*MRGPRX2*	33%	100%	50%	0%
Symptoms				
*Cutaneous*	96%	100%	95%	100%
*Mucosal*	54%	25%	58%	100%
*Grastrointestinal*	33%	0%	37%	100%
*Respiratory*	66%	0%	79%	100%
*Neurological*	8%	0%	5%	100%
*Cardiovascular*	8%	0%	5%	100%
Treatment				
*Epinephrine*	66%	25%	74%	100%
*H1 Receptor Antagonist*	100%	100%	100%	100%
*H2 Receptor Antagonist*	4%	0%	5%	0%
*Corticosteroids*	100%	100%	100%	100%
*Alcaloid*	4%	0%	5%	0%
*Cholinergic broncodilator*	8%	0%	11%	0%
*Glucopeptide*	4%	0%	5%	0%

F, female; SEM, standard error of the mean; IgE, IgE-mediated iDHR; COX, COX-1 inhibitors-mediated iDHR; MRGPRX2, MRGPRX2 receptor-mediated iDHR. Severity of the reactions was assigned following Browns’s classification (13).

**Figure 4:**
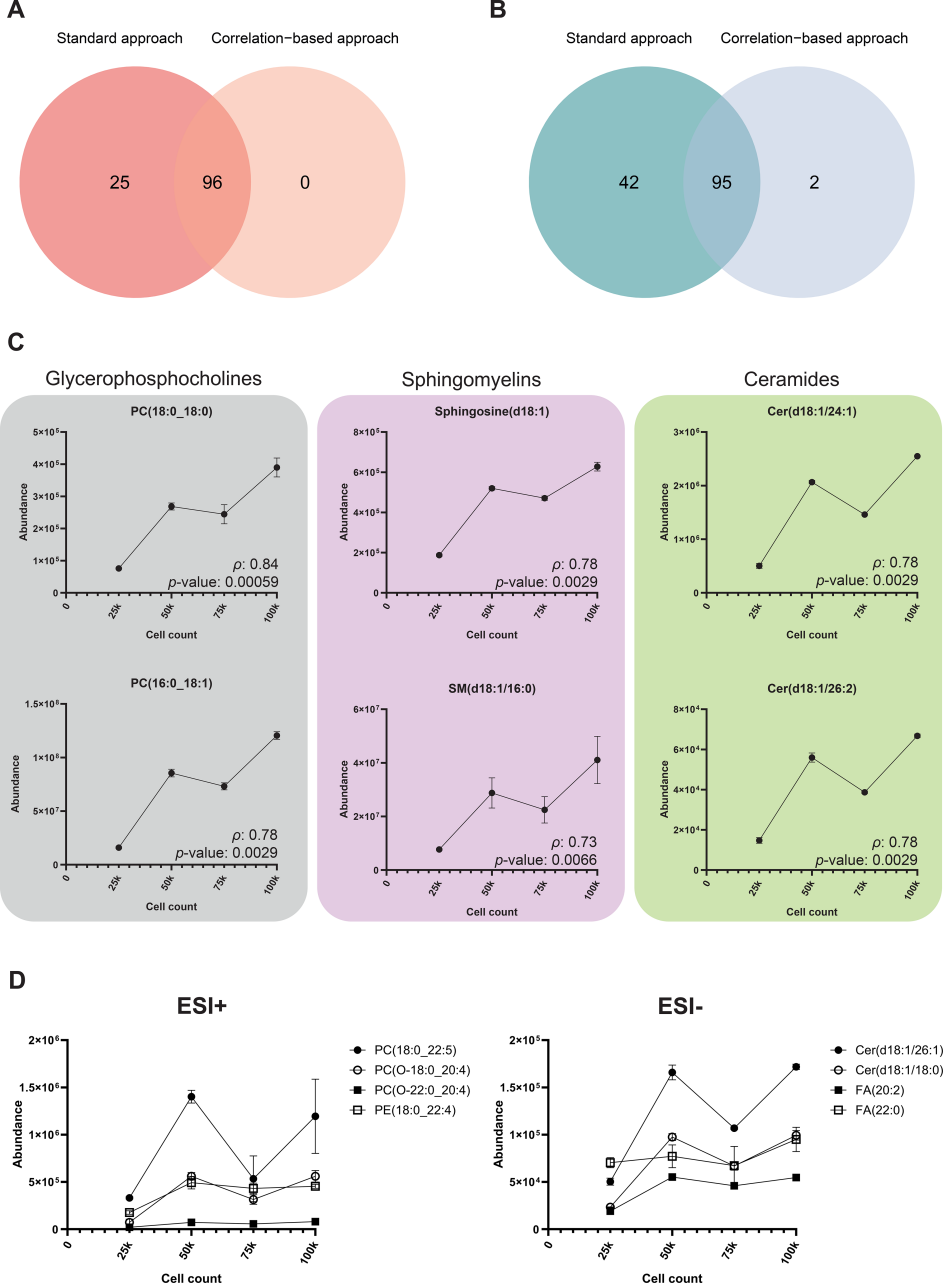
Comparison of significantly different chemical signals in HMVEC-d cohort after the exposure to patient sera in acute vs. basal stages by employing the standard and the correlation-based approaches. Venn diagrams for **(A)** ESI+ and **(B)** ESI- significant chemical signals after statistical analysis after the standard and the correlation-based approach. **(C)** Selected significant lipids from the intersection between the standard and the correlation based-approach (*p*-value < 0.05 and *p*-adj. value < 0.2) representing their abundance on the HMVEC-d cell curve. **(D)** Selected significant lipids only from the standard approach (*p*-value < 0.05 and *p*-adj. value < 0.2) representing their abundance on the HMVEC-d cell curve. Data are represented as mean (n= 3 per cell count) ± standard deviation (SD). PC, Glycerophosphocholine; SM, Sphingomyelin; Cer, Ceramide; PE, Glycerophosphoethanolamine; FA, Fatty Acid.

Statistical analysis by time (baseline vs. the acute stage), immunological mechanism (IgE, COX, and MRGPRX2) and their interaction was carried out in the two previously mentioned data sets (using the standard lipidomics approach and the correlation-based approach, **Supplementary Tables 7**, **8**, respectively). It is important to note that, for the correlation-based approach, significant differences were found only for time comparisons, and no statistical differences were found for the immunological mechanism or the interaction. However, for the standard approach, significant differences were found both for time and the interaction. The data set that followed the standard lipidomics approach (*k* = 408 and 580 for ESI+ and ESI-, respectively), showed 121 and 137 significantly different chemical signals (ESI+ and ESI-, respectively) in the HMVEC-d treated with sera from the baseline *vs.* the acute stage (*p*-value < 0.05 and *p*-adjusted value < 0.2) and 57 significantly different in ESI+ detected chemical signals due to the interaction between time and immunological mechanism. Interestingly, when chemical signals are selected using cell curve with their correlations (*k* = 157 and 276 for ESI+ and ESI-, respectively), 96 and 97 significantly different chemical signals in acute vs. basal timepoints (ESI+ and ESI-, respectively) were found (*p*-value < 0.05 and *p*-adjusted value < 0.2). Differences in these numbers of significant chemical signals were compared and represented using Venn diagrams for ESI+ and ESI- modes (**Figure 4A, B**). It is important to note that, if a cell curve is not analyzed along the experimental samples and the filtering step based on correlations counts is omitted, chemical signals not derived from cellular metabolism may be erroneously identified as significantly different in HMVEC-d following exposure to acute vs. baseline sera. As shown in **Figure 4A, B**, 25 and 42 additional chemical signals from ESI+ and ESI-, respectively, were identified as significantly different between mentioned conditions in the standard lipidomic approach; however, their origin cannot be confidently established. In both ionization modes, a substantial number of signals were shared between the two datasets (96 and 95 (79% and 68%) in ESI+ and ESI-, respectively). Importantly, the total number of chemical signals included in the statistical testing strongly influences multiple-testing correction and, consequently, adjusted *p*-values. Restricting the analysis to only chemical signals that correlate with cell counts reduces the multiple-testing burden, resulting in lower adjusted *p*-values and enabling a more sensitive detection of biologically relevant differences. Accordingly, two additional significant signals were detected exclusively in the correlation-filtered dataset in ESI- mode (**Figure 4B**), highlighting the advantage of excluding signals not associated with cellular metabolism. To know the behavior of significant signals in these two data sets (standard lipidomics approach and correlations-based approach), representative lipids were graphed on the data from cell curve (**Figure 4C**). As observed, clear linear tendencies were followed along the cell curve for the significant chemical signals after the analysis of correlations from the cell curve. This was not the case of the significant chemical signals that come only from standard lipidomics approach (**Figure 4D**) suggesting that their presence can be due to other non-related circumstances. This highlights the novelty of the proposed methodology for lipidomics cell analysis.

**Figure 5:**
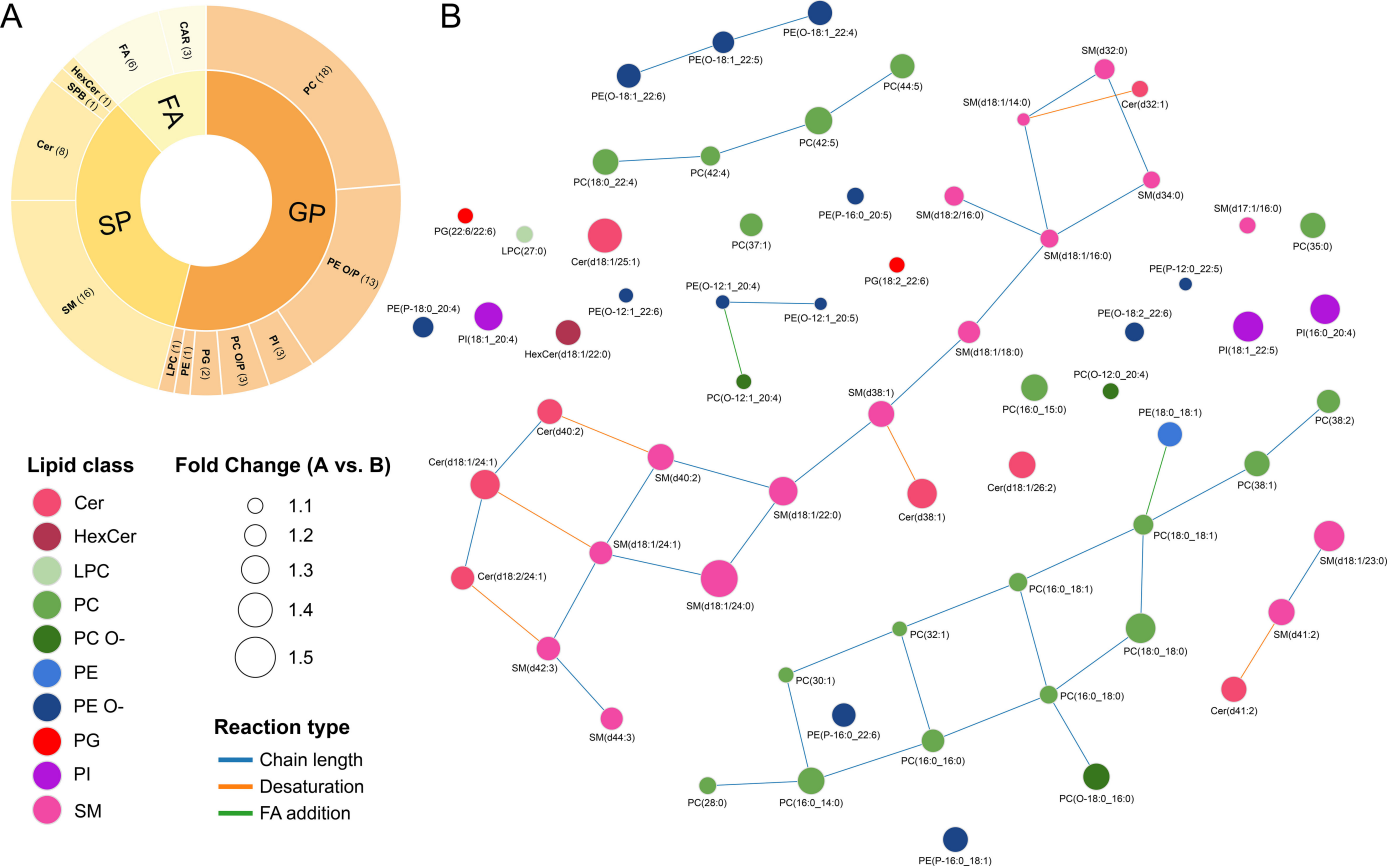
Differential lipid composition in HMVEC-d treated with serum from anaphylactic patients: acute vs. baseline stages. **(A)** Sunburst plot showing the proportion of different classes of lipids, according to LIPID MAPS (59). Classes: FA, Fatty Acyl/Alkyl/Alkenyl; SP, Sphingolipid; and GP, Glycerophospholipid. Subclasses: CAR, Acyl Carnitine; Cer, Ceramide; FA, Fatty Acid; HexCer, Hexosyl Ceramide; LPC, Lysoglycerophosphocholine; PC, Glycerophosphocholine; PC O/P, Alkyl-/Alkenyl- Glycerophosphocholine; PE, Glycerophosphoethanolamine; PE O/P, Alkyl-/Alkenyl- Glycerophosphoethanolamine; PG, Phosphoglycerol; PI, Phosphoinositol; SM, Sphingomyelin; and SPB, Sphingoid Base **(B)** Lipid-lipid network showing LINEX2 results. Nodes represent lipids, colored according to lipid classes and subclasses; node sizes represent the abundance Fold Change (Acute vs. Baseline); and edges represent reaction types connecting two nodes.

**Figure 6:**
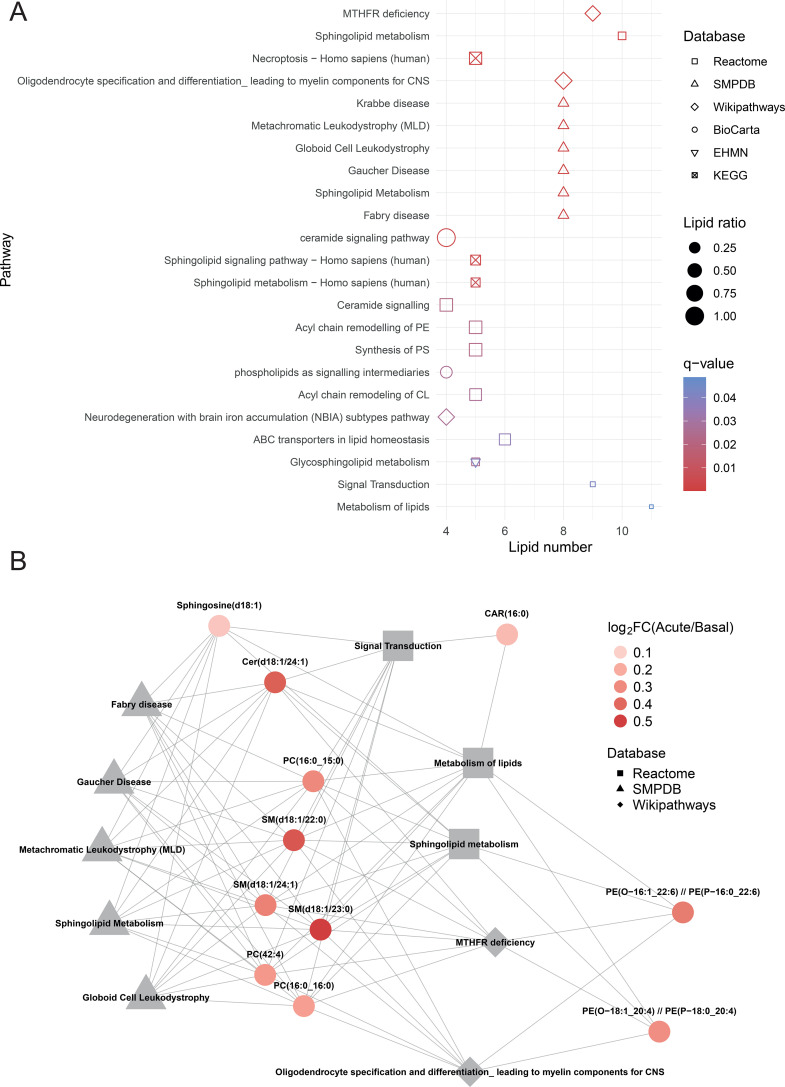
Overrepresentation pathway enrichment analysis. **(A)** Dotplot showing enriched terms with at least 4 overlapping lipids (*n* = 24), ordered by ascending *q*-value. Lipid ratio represents the number of overlapping lipids in each pathway divided by the total number of input lipids, and symbols shapes represent pathway source database. **(B)** Lipid–pathway network. Metabolite node colors represent log_2_FC (acute vs. basal), while pathway node shapes indicate the source database. Only the top 10 pathways ranked by the number of overlapping metabolites in descending order are shown.

Following with the significant chemical signals using the proposed methodology including the correlation step, out of the 193 significantly different signals between the acute and baseline stages, we were able to annotate 95, corresponding to 75 unique lipid species (**Supplementary Table 9**). The majority of these were glycerophospholipids, sphingolipids, fatty acids (FA), and acyl carnitines (Car) (**Figure 5A**), all of them increased in HMVEC-d after incubation with sera from the acute stage. LINEX^2^ analysis revealed a lipid-lipid network whose topology highlighted 2 key hubs, including ceramides (Cer) and sphingomyelins (SM) (hub 1), and glycerophosphocholines (PC) and glycerophosphoethanolamines (PE) (hub 2), which showed increased connectivity under the experimental comparison acute vs. baseline (**Figure 5B**). We subsequently conducted an enrichment analysis to identify the biological pathways altered in HMVEC-d after sera exposure (**Supplementary Table 10**). This analysis revealed alterations in sphingolipid metabolism, biosynthesis, and signaling pathways, and specifically in ceramide signaling. Notably, several of these pathways are linked to known metabolic and neurological disorders that are defined by defects in enzymes responsible for sphingolipid degradation or processing (**Figure 6A**) (60). The core group of lipids associated with the top 10 pathways (those with the highest number of overlapping lipids) included sphingosine(d18:1), Cer(d18:1/24:1), SM(d18:1/22:0), SM(d18:1/23:0), SM(d18:1/24:1), PC(16:0_16:0), PC(16:0_15:0), PC (42:4), and CAR(16:0), among others (**Figure 6B**).

In summary, these findings show that sera from anaphylactic patients collected at different stages induces distinct lipidomic changes in HMVEC-d, pointing to a potential involvement of endothelial lipid metabolism in anaphylaxis which seems not to be different between the immunological mechanisms of the reaction. Importantly, focusing on cell-derived signals reduced the multiple-testing burden, allowing these alterations to be identified with increased statistical confidence.

## Discussion

Cell metabolism is one of the key drivers of cell function and differentiation, particularly in immune cells (10, 61). This can be studied from multiple perspectives, including the analysis of real-time energetic metabolism, as performed by Seahorse Extracellular Flux analyzer (62) or, more recently, single-cell energetic metabolism by profiling translation inhibition (SCENITH) (63). However, while these techniques provide useful information about whether cells rely more on oxidative phosphorylation or glycolysis (64), they cannot determine the state of other key metabolic pathways, including those involving lipids (65–67). In this sense, untargeted cell lipidomics provides an essential tool to study the lipidome. However, it imposes a plethora of challenges, mainly in terms of discriminating lipids derived from cells from those derived from other sources. This aspect has, to our knowledge, never been considered in other studies, and it is very important as the proposed novel methodology could help determine whether the results reflect intrinsic cellular processes or are merely environmental artifacts, potentially leading to misinterpretation of the lipidomic data.

Additionally, the effect of different cell numbers analyzed by MS [as usually around 10^5^ to 10^7^ cells are reported suitable for metabolomics analyses (4)] has barely been reported (8). Other aspects such as the size and the metabolic state of the cells, should also be taken into account in lipidomics analysis.

For these reasons, we proposed a controlled experiment using up to 6 cell counts of CD3^+^ cells from a single donor ranging from 50k, 100k, 250k, 500k, 750k, and 1M. CD3^+^ cells have around 7-10 µm of diameter (68) and a highly active metabolism that is key for immune cell function and differentiation (10, 61). In this controlled experiment, we found that only after applying a correlation-based filter, we are able to select specific chemical signals that are coming from cells. This is translated in an increase in the explained variability in the first component of the PCA models from 60% to 90%. This means that with this approach we are reducing the variability coming from external sources not related specifically to the cells. Moreover, focusing on the positive correlated chemical signals, these are observed increased along the cell counts in a general view using heatmaps with HC, or in detail after selecting representative lipids such as glycerophosphocholines (PC), sphingomyelins (SM) and plasmalogens (PC(O-/P-). In this controlled setting, we also analyzed the cell media and found that it contained the same chemical signals as the cell counts. However, the corresponding signal intensities were not comparable, as the PCA revealed a markedly distinct clustering of the media samples and the cell counts, indicating that their lipid profile could not be directly compared with that of the cells. This is interesting because if chemical signals from cell media are used, they could mask the biological differences coming from the cells. Finally, with this CD3^+^ cell model, we were able to test the main normalization strategies usually applied in cell metabolomics (57) (median, cell number, and TUS). The results suggest that correlation-based analysis is needed in the three normalization strategies to increase the explained variability in the first component of the PCA models ranged from 94% to 99%. This pattern is not observed if the data is treated in a standard approach. In addition, we observed that after correlation-based analysis, the results of the three normalizations are comparable, as the choice of the normalization method will depend on the specifics of the experiment. For example, cell number can be applied only if cells can be accurately counted, whereas TUS and median can be used if they are representative, meaning that they have enough chemical signals to characterize the profile of the sample and a smaller number of unrelated signals. These ideas were also suggested by recent publications (8, 57).

Once the methodology was established, we decided to apply it in one case study (i.e., the HMVEC-d model). In a standard procedure only experimental HMVEC-d samples and QCs would be analyzed, however, to demonstrate the value of this novel methodology we included a cell curve of HMVEC-d. This was injected at the begging, middle, and end of the worklist. With this experiment we were able to confirm the results observed previously in the CD3^+^ cell model. Only after correlation-based analysis do the different cell counts cluster tightly, and the variance explained in the first component of the PCA models was around 90% for both polarities. Again, these positive correlated chemical signals in HMVEC-d were clearly observed increased along the cell counts in the heatmaps with HC. These results strengthen that the analysis of a cell curve within the experiment helps to select chemical signals that come only from the cells.

In this case, we were also able to study the ISTD added to all samples. These were used to confirm that the analytical performance was steady during the analysis and showed that their response was not affected by the increasing number of the cell counts. These findings suggest that the ISTD could be used for semi quantification of lipid species and other normalization strategies.

Interestingly, we observed that before and after applying the correlation-based approach to both cell cohorts (**Supplementary Figure 12**), HMVEC-d showed a higher homogeneity than CD3^+^ cell counts. This might be because HMVEC-d are a commercial cell line whereas CD3^+^ cells could be a pool of different subpopulation of lymphocytes (e.g., effector or regulatory T cells, among others).

Moving forward, the statistical analysis following the correlation-based approach revealed that, without this strategy, additional chemical signals (*k* = 67) would have been selected as significant following the standard approach. The origin of these chemical signals is unknown, but could come from the different treatments that were applied to each group of HMVEC-d. However, the significant chemical signals that passed the correlation-based analysis were known to come exclusively from the cells.

Regarding the application of this methodology, we know that the endothelium is considered relevant in anaphylaxis (69); thus, HMVEC-d represents a key model to study their participation in this disease as previously reported (70, 71). Therefore, we sought to explore how the lipidome of HMVEC-d is changed after exposure to serum from patients suffering an anaphylactic reaction and following a specific immunological mechanism (IgE, COX-1, and MRGPRX2). In this, our proposed methodology was critical to unsure that the differences found were due to specific lipids that were produced and/or derived from these cells, and not from the treatment.

Interestingly, we did not find significant differences in the HMVEC-d lipidome when comparing for the different mechanisms (IgE, COX-1, and MRGPRX2). These findings point out that iDHR converge on common intracellular signaling hubs despite being initiated by distinct surface receptors. Although IgE-mediated activation through FcϵRI, COX-dependent pathways, and MRGPRX2 signaling are triggered by different upstream mechanisms, they share key downstream intermediates, most notably phospholipase C (PLC) and phosphoinositide 3-kinase (PI3K). In IgE–FcϵRI signaling, receptor aggregation leads to Syk activation and recruitment of the LAT signalosome, which in turn engages PLCγ1/2 and activates PI3K through the adaptor protein GAB2 (72). In contrast, both COX-derived lipid mediators (such as prostaglandins) and MRGPRX2 ligands signal through G protein–coupled receptors, predominantly coupling to Gq/G11 proteins and activating PLCβ, with concomitant engagement of PI3K-dependent pathways (73). Despite these receptor- and isoform-specific differences in proximal signaling, all three mechanisms converge on shared second messengers and downstream lipid-modifying enzymes, thereby driving overlapping lipid remodeling programs. This convergence is expected to result in similar alterations in phospholipid composition, sphingosine-1-phosphate biosynthesis, and ceramide (Cer) metabolism, ultimately yielding comparable endothelial lipidomic profiles. Additionally, it could be possible that the chosen stimulation time may not be optimal to notice mechanism-specific differences, as distinct anaphylactic activation pathways are likely to generate lipidomic signatures with divergent early kinetics that progressively converge at later stages of the inflammatory response.

Even though we did not find differences between mechanisms, we did observe significant alterations in the lipidome over time. The functional enrichment analysis of the lipids whose abundances are significantly different between the acute and basal phases points to alterations in metabolic pathways related to sphingolipid metabolism, biosynthesis, and signaling in HMVEC-d exposed to patient sera obtained during anaphylactic reactions. Notably, Cer signaling was one of the most affected pathways, which could be particularly relevant given the critical role of Cer in controlling growth regulation, cell migration, adhesion, apoptosis, senescence, and inflammatory responses (74).

A core group of lipids, including sphingosine(d18:1), Cer(d18:1/24:1), and several SMs and PCs was found to be associated with most of the top enriched pathways. This suggests a coordinated activation of the Cer–sphingosine–sphingosine-1-phosphate axis, which is involved in cell growth, survival, inflammation, and tissue remodeling (75). These findings support the idea that HMVEC-d adjust their lipidomic profile during anaphylactic reactions, potentially reflecting a protective or stress-adaptative mechanism.

Interestingly, many of the enriched pathways seemed to also be implicated in metabolic and neurological disorders characterized by defects in sphingolipid degradation or processing (60). This finding would indicate that lipids released during anaphylaxis could activate HMVEC-d, leading to the release of sphingolipids that might either feed back into the reaction or exert a protective effect. From an allergology perspective, these findings could be particularly relevant in the search for predictive and/or monitoring biomarkers. Exposing endothelial cells to patients’ sera prior to drug administration could help identify individuals at higher risk of severe reactions. During the acute stage of the anaphylaxis, changes in sphingolipid and phospholipid pathways might serve as indicators of disease dynamics. Moreover, the consistent involvement of Cer and sphingosine-1-phosphate signaling in vascular biology suggests potential therapeutic targets that could be exploited in future interventions (76).

To summarize, the proposed methodology based on including a cell curve during cell lipidomics analysis presents several advantages. This is, to the best of our knowledge, the first approach that has been described as a way of selecting specific chemical signals coming from cells. Correlation-based analysis allowed to reduce unrelated noise, helping to improve statistical power such as multiple test correction (*p*-adjusted value) and avoiding false-positive findings.

Nonetheless, we acknowledge several limitations to this methodology. Generation of the cell curve might be unfeasible for extremely limited cell populations. Additionally, as this approach captures changes within cell-derived lipids that are already present before the stimuli, some information about newly produced metabolites might be lost. Finally, for the pathway enrichment analysis as the HMDB does not separate isomers and the pathways are still not fully described, the interpretation of these results can be overstated.

Altogether, we present a cell curve approach and a correlation-based analysis for untargeted cell lipidomics that enables the selective identification of chemical signals intrinsically derived from cellular lipidome by exploiting their association with cell count. This strategy effectively reduces confounding signals, improves statistical power after multiple-testing correction, and enhances the detection of biologically meaningful lipid changes. Applying this approach to HMVEC-d, we uncovered a specific lipidic response from serum of patients with anaphylaxis, characterized by alterations in sphingolipid and glycerophospholipid pathways, highlighting the endothelial contribution to anaphylaxis and suggesting promising avenues for further studies. Overall, this methodology provides a robust and broadly applicable framework for the analysis and functional interpretation of cell-derived lipidomes across different cellular models.

## Data Availability

The data presented in the study are deposited in the Metabolomics Workbench database, under project number PR002888 (doi: 10.21228/M8C569), which includes two separate studies: ST004583 (relating to CD3^+^ cells) and ST004584 (relating to HMVEC-d cells).

## References

[1] Loftus RM Finlay DK . Immunometabolism: cellular metabolism turns immune regulator. J Biol Chem. (2016) 291:1–10. doi: 10.1074/jbc.R115.693903, PMID: 26534957 PMC4697146

[2] Makowski L Chaib M Rathmell JC . Immunometabolism: From basic mechanisms to translation. Immunol Rev. (2020) 295:5–14. doi: 10.1111/imr.12858, PMID: 32320073 PMC8056251

[3] Chakraborty S Khamaru P Bhattacharyya A . Regulation of immune cell metabolism in health and disease: Special focus on T and B cell subsets. Cell Biol Int. (2022) 46:1729–46. doi: 10.1002/cbin.11867, PMID: 35900141

[4] Schönberger K Mitterer M Glaser K Stecher M Hobitz S Schain-Zota D . LC-MS-based targeted metabolomics for FACS-purified rare cells. Anal Chem. (2023) 95:4325–34. doi: 10.1021/acs.analchem.2c04396, PMID: 36812587 PMC9996616

[5] DeVilbiss AW Zhao Z Martin-Sandoval MS Ubellacker JM Tasdogan A Agathocleous M . Metabolomic profiling of rare cell populations isolated by flow cytometry from tissues. eLife. (2021) 10:e61980. doi: 10.7554/eLife.61980, PMID: 33470192 PMC7847306

[6] Luo X Li L . Metabolomics of small numbers of cells: metabolomic profiling of 100, 1000, and 10000 Human breast cancer cells. Anal Chem. (2017) 89:11664–71. doi: 10.1021/acs.analchem.7b03100, PMID: 29028307

[7] Xu F Song C Liu W Chen G . Protocol for intracellular and extracellular metabolite detection in human embryonic stem cells. STAR Protoc. (2021) 2:100740. doi: 10.1016/j.xpro.2021.100740, PMID: 34467226 PMC8387572

[8] Basov NV Butikova EA Sotnikova MA Razumov IA Sotnikova YS Patrushev YV . Features of sample preparation of cell culture samples for metabolomic screening by LC-MS/MS. J Pharm Biomed Anal. (2025) 267:117146. doi: 10.1016/j.jpba.2025.117146, PMID: 40972480

[9] Reinalda L van der Stelt M Van Kasteren SI . Lipid metabolism and immune function: chemical tools for insights into T-cell biology. ChemBioChem. (2025) 26:e202400980. doi: 10.1002/cbic.202400980, PMID: 40162512 PMC12135138

[10] Ma S Ming Y Wu J Cui G . Cellular metabolism regulates the differentiation and function of T-cell subsets. Cell Mol Immunol. (2024) 21:419–35. doi: 10.1038/s41423-024-01148-8, PMID: 38565887 PMC11061161

[11] Pablo-Torres C Delgado-Dolset MI Sanchez-Solares J Mera-Berriatua L Núñez Martín Buitrago L Reaño Martos M . A method based on plateletpheresis to obtain functional platelet, CD3^+^ and CD14^+^ matched populations for research immunological studies. Clin Exp Allergy. (2022) 52:1157–68. doi: 10.1111/cea.14192, PMID: 35757844 PMC9796013

[12] Sampson HA Muñoz-Furlong A Campbell RL Adkinson NF Bock SA Branum A . Second symposium on the definition and management of anaphylaxis: Summary report—Second National Institute of Allergy and Infectious Disease/Food Allergy and Anaphylaxis Network symposium. J Allergy Clin Immunol. (2006) 117:391–7. doi: 10.1016/j.jaci.2005.12.1303, PMID: 16461139

[13] Brown SGA . Clinical features and severity grading of anaphylaxis. J Allergy Clin Immunol. (2004) 114:371–6. doi: 10.1016/j.jaci.2004.04.029, PMID: 15316518

[14] Bavbek S Kepil Özdemir S Bonadonna P Atanaskovic-Markovic M Barbaud A Brockow K . Hypersensitivity reactions to proton pump inhibitors. EAACI position paper. Allergy. (2024) 79:552–64. doi: 10.1111/all.15961, PMID: 38013608

[15] Romano A Atanaskovic-Markovic M Barbaud A Bircher AJ Brockow K Caubet J-C . Towards a more precise diagnosis of hypersensitivity to beta-lactams - an EAACI position paper. Allergy. (2020) 75:1300–15. doi: 10.1111/all.14122, PMID: 31749148

[16] Doña I Pérez-Sánchez N Bogas G Moreno E Salas M Torres MJ . Medical algorithm: Diagnosis and treatment of nonsteroidal antiinflammatory drugs hypersensitivity. Allergy. (2020) 75:1003–5. doi: 10.1111/all.14119, PMID: 31742729

[17] Sabato V Ebo DG van der Poorten M-LM Toscano A Van Gasse AL Mertens C . Allergenic and mas-related G protein-coupled receptor X2-activating properties of drugs: resolving the two. J Allergy Clin Immunol Pract. (2023) 11:395–404. doi: 10.1016/j.jaip.2022.12.014, PMID: 36581077

[18] Yuste-Montalvo A Fernandez-Bravo S Oliva T Pastor-Vargas C Betancor D Goikoetxea MJ . Proteomic and biological analysis of an *in vitro* human endothelial system in response to drug anaphylaxis. Front Immunol. (2021) 12:692569. doi: 10.3389/fimmu.2021.692569, PMID: 34248989 PMC8269062

[19] Martínez S Fernández-García M Londoño-Osorio S Barbas C Gradillas A . Highly reliable LC-MS lipidomics database for efficient human plasma profiling based on NIST SRM 1950. J Lipid Res. (2024) 65:100671. doi: 10.1016/j.jlr.2024.100671, PMID: 39395790 PMC11607663

[20] Gonzalez-Riano C Gradillas A Barbas C . Exploiting the formation of adducts in mobile phases with ammonium fluoride for the enhancement of annotation in liquid chromatography-high resolution mass spectrometry based lipidomics. J Chromatogr Open. (2021) 1:100018. doi: 10.1016/j.jcoa.2021.100018

[21] Sud M Fahy E Cotter D Azam K Vadivelu I Burant C . Metabolomics Workbench: An international repository for metabolomics data and metadata, metabolite standards, protocols, tutorials and training, and analysis tools. Nucleic acids research. (2016) 44(D1):D463–D470. doi: 10.1093/nar/gkv1042, PMID: 26467476 PMC4702780

[22] Wickham H François R Henry L Müller K Vaughan D . dplyr: A grammar of data manipulation. (2023) 1.1:4. doi: 10.32614/CRAN.package.dplyr

[23] Sánchez-Illana Á Piñeiro-Ramos JD Sanjuan-Herráez JD Vento M Quintás G Kuligowski J . Evaluation of batch effect elimination using quality control replicates in LC-MS metabolite profiling. Analytica Chimica Acta. (2018) 1019:38–48. doi: 10.1016/j.aca.2018.02.053, PMID: 29625683

[24] Kowarik A Templ M . Imputation with the *R* package VIM. J Stat Soft. (2016) 74. doi: 10.18637/jss.v074.i07

[25] Alfons A Templ M . Estimation of social exclusion indicators from complex surveys: the *R* package laeken. J Stat Soft. (2013) 54. doi: 10.18637/jss.v054.i15

[26] Troyanskaya O Cantor M Sherlock G Brown P Hastie T Tibshirani R . Missing value estimation methods for DNA microarrays. Bioinformatics. (2001) 17:520–5. doi: 10.1093/bioinformatics/17.6.520, PMID: 11395428

[27] Armitage EG Godzien J Alonso-Herranz V López-Gonzálvez Á Barbas C . Missing value imputation strategies for metabolomics data. Electrophoresis. (2015) 36:3050–60. doi: 10.1002/elps.201500352, PMID: 26376450

[28] R Core Team . R: A language and environment for statistical computing (2024). Available online at: https://www.R-project.org/ (Accessed June 20, 2024).

[29] Kuligowski J Sánchez-Illana Á Sanjuán-Herráez D Vento M Quintás G . Intra-batch effect correction in liquid chromatography-mass spectrometry using quality control samples and support vector regression (QC-SVRC). Analyst. (2015) 140:7810–7. doi: 10.1039/C5AN01638J, PMID: 26462549

[30] Rodríguez-Coira J Delgado-Dolset M Obeso D Dolores-Hernández M Quintás G Angulo S . Troubleshooting in large-scale LC-toF-MS metabolomics analysis: solving complex issues in big cohorts. Metabolites. (2019) 9:247. doi: 10.3390/metabo9110247, PMID: 31652940 PMC6918290

[31] Mukaka MM . Statistics corner: A guide to appropriate use of correlation coefficient in medical research. Malawi Med J. (2012) 24:69–71., PMID: 23638278 PMC3576830

[32] Kassambara A . rstatix: pipe-friendly framework for basic statistical tests. (2023) 0.7:2. doi: 10.32614/CRAN.package.rstatix

[33] Lenth RV . emmeans: estimated marginal means, aka least-squares means. (2025) 1.11:2–8. doi: 10.32614/CRAN.package.emmeans

[34] Kay M Elkin L Higgins JJ Wobbrock JO . mjskay/ARTool: ARTool 0.11.2. (2025). doi: 10.5281/ZENODO.594511.

[35] Elkin LA Kay M Higgins JJ Wobbrock JO . An aligned rank transform procedure for multifactor contrast tests. In: The 34th annual ACM symposium on user interface software and technology. Virtual Event USA, ACM (2021). p. 754–68. doi: 10.1145/3472749.3474784

[36] Wobbrock JO Findlater L Gergle D Higgins JJ . The aligned rank transform for nonparametric factorial analyses using only anova procedures. In: Proceedings of the SIGCHI conference on human factors in computing systems. ACM, Vancouver BC Canada (2011). p. 143–6. doi: 10.1145/1978942.1978963

[37] Singmann H Bolker B Westfall J Aust F Ben-Shachar MS . afex: analysis of factorial experiments. (2024) 1:5–0. doi: 10.32614/CRAN.package.afex

[38] Vinaixa M Samino S Saez I Duran J Guinovart JJ Yanes O . A guideline to univariate statistical analysis for LC/MS-based untargeted metabolomics-derived data. Metabolites. (2012) 2:775–95. doi: 10.3390/metabo2040775, PMID: 24957762 PMC3901240

[39] Rago D Pedersen C-ET Huang M Kelly RS Gürdeniz G Brustad N . Characteristics and mechanisms of a sphingolipid-associated childhood asthma endotype. Am J Respir Crit Care Med. (2021) 203:853–63. doi: 10.1164/rccm.202008-3206OC, PMID: 33535020 PMC8017574

[40] Tsugawa H Cajka T Kind T Ma Y Higgins B Ikeda K . MS-DIAL: data-independent MS/MS deconvolution for comprehensive metabolome analysis. Nat Methods. (2015) 12:523–6. doi: 10.1038/nmeth.3393, PMID: 25938372 PMC4449330

[41] Horai H Arita M Kanaya S Nihei Y Ikeda T Suwa K . MassBank: a public repository for sharing mass spectral data for life sciences. J Mass Spectrom. (2010) 45:703–14. doi: 10.1002/jms.1777, PMID: 20623627

[42] Kim S Chen J Cheng T Gindulyte A He J He S . PubChem 2025 update. Nucleic Acids Res. (2025) 53:D1516–25. doi: 10.1093/nar/gkae1059, PMID: 39558165 PMC11701573

[43] Conroy MJ Andrews RM Andrews S Cockayne L Dennis EA Fahy E . LIPID MAPS: update to databases and tools for the lipidomics community. Nucleic Acids Res. (2024) 52:D1677–82. doi: 10.1093/nar/gkad896, PMID: 37855672 PMC10767878

[44] Wishart DS Guo A Oler E Wang F Anjum A Peters H . HMDB 5.0: the human metabolome database for 2022. Nucleic Acids Res. (2022) 50:D622–31. doi: 10.1093/nar/gkab1062, PMID: 34986597 PMC8728138

[45] Pang Z Lu Y Zhou G Hui F Xu L Viau C . MetaboAnalyst 6.0: towards a unified platform for metabolomics data processing, analysis and interpretation. Nucleic Acids Res. (2024) 52:W398–406. doi: 10.1093/nar/gkae253, PMID: 38587201 PMC11223798

[46] Kamburov A Cavill R Ebbels TMD Herwig R Keun HC . Integrated pathway-level analysis of transcriptomics and metabolomics data with IMPaLA. Bioinformatics. (2011) 27:2917–8. doi: 10.1093/bioinformatics/btr499, PMID: 21893519

[47] Gu Z . colorRamp2: generate color mapping functions. (2022) 0.1:0. doi: 10.32614/CRAN.package.colorRamp2

[48] Schloerke B Cook D Larmarange J Briatte F Marbach M Thoen E . GGally: extension to “ggplot2. ”. (2024) 2.4: doi: 10.32614/CRAN.package.GGally

[49] Fc M Davis TL . ggplot2 authors. ggpattern: “ggplot2” Pattern Geoms. (2025) 1.2:1. doi: 10.32614/CRAN.package.ggpattern

[50] Kassambara A . ggpubr: “ggplot2” Based publication ready plots. (2023) 0.6:1. doi: 10.32614/CRAN.package.ggpubr

[51] Wickham H Chang W Henry L Pedersen TL Takahashi K Wilke C . ggplot2: create elegant data visualisations using the grammar of graphics. (2025) 4.0:0. doi: 10.32614/CRAN.package.ggplot2

[52] Butts CT . network: classes for relational data. (2015) 1.19:0. doi: 10.32614/CRAN.package.network

[53] Butts CT . network : A package for managing relational data in. R J Stat Soft. (2008) 24. doi: 10.18637/jss.v024.i02

[54] Rose TD Köhler N Falk L Klischat L Lazareva OE Pauling JK . Lipid network and moiety analysis for revealing enzymatic dysregulation and mechanistic alterations from lipidomics data. Briefings Bioinf. (2023) 24:bbac572. doi: 10.1093/bib/bbac572, PMID: 36592059 PMC9851308

[55] Milacic M Beavers D Conley P Gong C Gillespie M Griss J . The reactome pathway knowledgebase 2024. Nucleic Acids Res. (2024) 52:D672–8. doi: 10.1093/nar/gkad1025, PMID: 37941124 PMC10767911

[56] Bansal P Morgat A Axelsen KB Muthukrishnan V Coudert E Aimo L . Rhea, the reaction knowledgebase in 2022. Nucleic Acids Res. (2022) 50:D693–700. doi: 10.1093/nar/gkab1016, PMID: 34755880 PMC8728268

[57] Cuevas-Delgado P Dudzik D Miguel V Lamas S Barbas C . Data-dependent normalization strategies for untargeted metabolomics—a case study. Anal Bioanal Chem. (2020) 412:6391–405. doi: 10.1007/s00216-020-02594-9, PMID: 32285184

[58] Binek A Rojo D Godzien J Rupérez FJ Nuñez V Jorge I . Flow cytometry has a significant impact on the cellular metabolome. J Proteome Res. (2019) 18:169–81. doi: 10.1021/acs.jproteome.8b00472, PMID: 30362351

[59] Liebisch G Fahy E Aoki J Dennis EA Durand T Ejsing CS . Update on LIPID MAPS classification, nomenclature, and shorthand notation for MS-derived lipid structures. J Lipid Res. (2020) 61:1539–55. doi: 10.1194/jlr.S120001025, PMID: 33037133 PMC7707175

[60] Allende ML Zhu H Kono M Hoachlander-Hobby LE Huso VL Proia RL . Genetic defects in the sphingolipid degradation pathway and their effects on microglia in neurodegenerative disease. Cell Signalling. (2021) 78:109879. doi: 10.1016/j.cellsig.2020.109879, PMID: 33296739 PMC7775721

[61] Cao D Bergmann J Zhong L Hemalatha A Dingare C Jensen T . Selective utilization of glucose metabolism guides mammalian gastrulation. Nature. (2024) 634:919–28. doi: 10.1038/s41586-024-08044-1, PMID: 39415005 PMC11499262

[62] Yoo I Ahn I Lee J Lee N . Extracellular flux assay (Seahorse assay): Diverse applications in metabolic research across biological disciplines. Molecules Cells. (2024) 47:100095. doi: 10.1016/j.mocell.2024.100095, PMID: 39032561 PMC11374971

[63] Argüello RJ Combes AJ Char R Gigan J-P Baaziz AI Bousiquot E . SCENITH: A flow cytometry-based method to functionally profile energy metabolism with single-cell resolution. Cell Metab. (2020) 32:1063–1075.e7. doi: 10.1016/j.cmet.2020.11.007, PMID: 33264598 PMC8407169

[64] Li L Zhdanov AV Papkovsky DB . Optical sensor-based systems for the analysis of cell metabolism and bioenergetics. Sensors Actuators B: Chem. (2025) 429:137303. doi: 10.1016/j.snb.2025.137303

[65] Tomé D . Amino acid metabolism and signalling pathways: potential targets in the control of infection and immunity. Nutr Diabetes. (2021) 11:20. doi: 10.1038/s41387-021-00164-1, PMID: 34168115 PMC8223530

[66] Castellano F Molinier-Frenkel V . Control of T-cell activation and signaling by amino-acid catabolizing enzymes. Front Cell Dev Biol. (2020) 8:613416. doi: 10.3389/fcell.2020.613416, PMID: 33392202 PMC7773816

[67] Lim SA Su W Chapman NM Chi H . Lipid metabolism in T cell signaling and function. Nat Chem Biol. (2022) 18:470–81. doi: 10.1038/s41589-022-01017-3, PMID: 35484263 PMC11103273

[68] Lewis DE Harriman GR Blutt SE . “Organization of the immune system.,”. In: Clinical immunology. Amsterdam, Netherlands: Elsevier (2013). p. 16–34. doi: 10.1016/B978-0-7234-3691-1.00026-X

[69] Nuñez-Borque E Fernandez-Bravo S Yuste-Montalvo A Esteban V . Pathophysiological, cellular, and molecular events of the vascular system in anaphylaxis. Front Immunol. (2022) 13:836222. doi: 10.3389/fimmu.2022.836222, PMID: 35371072 PMC8965328

[70] Pasut A Lama E Van Craenenbroeck AH Kroon J Carmeliet P . Endothelial cell metabolism in cardiovascular physiology and disease. Nat Rev Cardiol. (2025) 22:923–43. doi: 10.1038/s41569-025-01162-x, PMID: 40346347

[71] Carlsen J Henriksen HH Marin De Mas I Johansson PI . An explorative metabolomic analysis of the endothelium in pulmonary hypertension. Sci Rep. (2022) 12:13284. doi: 10.1038/s41598-022-17374-x, PMID: 35918401 PMC9345936

[72] Metcalfe DD Peavy RD Gilfillan AM . Mechanisms of mast cell signaling in anaphylaxis. J Allergy Clin Immunol. (2009) 124:639–46. doi: 10.1016/j.jaci.2009.08.035, PMID: 19815110 PMC2788154

[73] Nguyen SMT Rupprecht CP Haque A Pattanaik D Yusin J Krishnaswamy G . Mechanisms governing anaphylaxis: inflammatory cells, mediators, endothelial gap junctions and beyond. IJMS. (2021) 22:7785. doi: 10.3390/ijms22157785, PMID: 34360549 PMC8346007

[74] Hannun YA Obeid LM . Sphingolipids and their metabolism in physiology and disease. Nat Rev Mol Cell Biol. (2018) 19:175–91. doi: 10.1038/nrm.2017.107, PMID: 29165427 PMC5902181

[75] Díaz-Perales A Escribese MM Garrido-Arandia M Obeso D Izquierdo-Alvarez E Tome-Amat J . The role of sphingolipids in allergic disorders. Front Allergy. (2021) 2:675557. doi: 10.3389/falgy.2021.675557, PMID: 35386967 PMC8974723

[76] Jozefczuk E Guzik TJ Siedlinski M . Significance of sphingosine-1-phosphate in cardiovascular physiology and pathology. Pharmacol Res. (2020) 156:104793. doi: 10.1016/j.phrs.2020.104793, PMID: 32278039

