# Supplementary material for: Establishing an untargeted lipidomics workflow for cellular analysis: insights into endothelial cell function in anaphylaxis
Source: Front Immunol. 2026 Mar 4;17:1711640. doi: 10.3389/fimmu.2026.1711640 (PMC12997047; doi:10.3389/fimmu.2026.1711640)
Supplement: Supplementary file 1 [file DataSheet1.docx]

SUPPLEMENTARY INFORMATION

Establishing an Untargeted Lipidomics Workflow for Cellular Analysis: Insights into Endothelial Cell Function in Anaphylaxis

**María Isabel Delgado Dolset****^1,2^**†**, Andrea Escolar-Peña^2^**†**, Sergio Fernández-Bravo^3^, Antonio J. García-Cívico^1,2^, Lucía Pajares^1,2^, René Neuhaus^1^, Rosario González-Mendiola^4,5^, Coral Barbas^1^, Domingo Barber^2^, José Julio Laguna^4,5^, María M Escribese^2^, Vanesa Esteban^3,5*^, Alma Villaseñor^1,2*^.**

^1^Centro de Metabolómica y Bioanálisis (CEMBIO), Facultad de Farmacia, Universidad San Pablo-CEU, CEU Universities, Urbanización Montepríncipe, 28660 Boadilla del Monte, España.

^2^Departamento de Ciencias Médicas Básicas, Facultad de Medicina, Instituto de Medicina Molecular Aplicada – Nemesio Díez (IMMA-ND), Universidad San Pablo-CEU, CEU Universities, Madrid, España.

^3^Department of Allergy and Immunology, IIS-Fundación Jiménez Díaz, UAM, Madrid, Spain

^4^Allergy Unit, Allergo-Anaesthesia Unit, Hospital Central de la Cruz Roja, Madrid, Spain

^5^ Faculty of Biomedical and Health Sciences, Alfonso X El Sabio University, Madrid, Spain.

†These authors contributed equally to this work and share first authorship

*These authors contributed equally

***Correspondence:**Alma Villaseñor
alma.villasenor@ceu.es

**Table of Contents**

**Supplementary Figures**. This includes:

**Supplementary Figure 1:** Histograms of missing values reported per chemical signal and per sample in the CD3^+^ cell model.

**Supplementary Figure 2:** PCA models generated using all detected chemical signals after QA process (*k*=1087 in ESI+ and *k*=249 in ESI-) in CD3^+^ cell model.

**Supplementary Figure 3:** Correlated chemical signals allow an accurate grouping of samples by cell count in CD3^+^ cells model for ESI- mode.

**Supplementary Figure 4:** Cell count-dependent abundance of identified significant lipids resulting after applying correlation-based filtering in CD3^+^ cells.

**Supplementary Figure 5:** PCA models generated using detected chemical signals after QA process in CD3+ model analyzed by ESI+ mode.

**Supplementary Figure 6:** PCA models generated using detected chemical signals after QA process in CD3+ model analyzed by ESI- mode.

**Supplementary Figure 7:** Effect of data normalization on selected lipid subclasses.

**Supplementary Figure 8:** Histograms of missing values reported per chemical signal and per sample in the CD3^+^ cell model.

**Supplementary Figure 9:** PCA models generated using all detected chemical signals after QA process (*k*=408 in ESI+ and *k*=580 in ESI-) in HMVEC-d cells.

**Supplementary Figure 10:** Correlated chemical signals allow an accurate grouping of samples by cell count in HMVEC-d model for ESI- mode.

**Supplementary Figure 11**: Behavior of the internal standards (ISTDs) added to HMVEC-d samples, throughout the chromatographic analysis.

**Supplementary Figure 12:** Comparison of PCA models using chemical signals obtained applying the standard approach and the correlation-based approach for CD3^+^ and HMVEC-d models in ESI+ and ESI- modes.

**Supplementary Tables**. Due to the extension, these are given in separate excel files. These include:

**Supplementary Table 1:** Spearman correlations between the chemical signal abundance and the cell count at different cell count intervals (*p-*value < 0.05 and *ρ* ≥ 0.7) for CD3^+^ cells in ESI+ mode.

**Supplementary Table 2:** Spearman correlations between the chemical signal abundance and the cell count at different cell count intervals (*p-*value < 0.05 and *ρ* ≥ 0.7) for CD3^+^ cells in ESI- mode.

**Supplementary Table 3:** Spearman correlations between the chemical signal abundance and the cell count at different cell count intervals (*p-*value < 0.05 and *ρ* ≥ 0.7) for HMVEC-d in ESI+ mode.

**Supplementary Table 4:** Spearman correlations between the chemical signal abundance and the cell count at different cell count intervals (*p-*value < 0.05 and *ρ* ≥ 0.7) for HMVEC-d in ESI- mode.

**Supplementary Table 5:** Coefficient of variation (%CV) of selected non-endogenous internal standards (ISTD) in QC samples and cell curves. Each ISTD was used depending on their preferred ionization mode.

**Supplementary Table 6:** Individual clinical characteristics of the enrolled patients suffering anaphylaxis.

**Supplementary Table 7:** ANOVA/Aligned-Rank Transform (ART) ANOVA statistical analyses result for all chemical signals that passed the QA in the HMVEC-d model following the standard approach. For each chemical signal identification, *p*-value and *p*-adj. value are reported for each factor examined (mechanism and time) and the interaction between them. Applied statistical test and ESI mode are also provided. *p*-value < 0.05 and *p*-adj. value < 0.2 are highlighted in green.

**Supplementary Table 8:** ANOVA/Aligned-Rank Transform (ART) ANOVA statistical analyses result for all chemical signals that passed the QA in the HMVEC-d model after the correlation-based analysis. For each chemical signal identification, *p*-value and *p*-adj. value are reported for each factor examined (mechanism and time) and the interaction between them. Applied statistical test and ESI mode are also provided. *p*-value < 0.05 and *p*-adj. value < 0.2 are highlighted in green.

**Supplementary Table 9:** Lipid annotations, abundance fold changes, and features. Classes: FA, Fatty Acyl/Alkyl/ Alkenyl; SP, Sphingolipid; and GP, Glycerophospholipid. Subclasses: CAR, Acyl Carnitine; Cer, Ceramide; FA, Fatty Acid; HexCer, Hexosyl Ceramide; LPC, Lysoglycerophosphocholine; PC, Glycerophosphocholine; PC O/P, Alkyl-/Alkenyl- Glycerophosphocholine; PE, Glycerophosphoethanolamine; PE O/P, Alkyl-/Alkenyl- Glycerophosphoethanolamine; PG, Glycerophosphoglycerol; PI, Glycerophosphoinositol; SM, Sphingomyelin; and SPB, Sphingoid Base. NA means not available.

**Supplementary Table 10:** IMPaLA Over Representation Analysis results from identified lipids with an available HMDB ID (n=44). Pathway name, pathway source (data base), number of overlapping metabolites, overlapping metabolites, number of metabolites associated to the pathway in the database, *p*-value and q-value are reported for each enriched pathway.

**Supplementary Figure 1.**  **Histograms of missing values reported per chemical signal and per sample in the CD3+ cell model.** Histograms show the number of chemical signals with a specific percentage of missing values in **(A)** ESI + and **(B)** ESI -. Bar plots showing the missing values percentage per sample (colored according to experimental groups) in **(C)** ESI + and **(D)** ESI -.

**Supplementary Figure 2: PCA models generated using all detected chemical signals after QA process (*k*=1087 in ESI+ and *k*=249 in ESI-) in CD3^+^ cells.** The model includes the cell counts, cell media, and quality controls (QC) for ESI+ (**A**) and ESI- (**B**) modes.


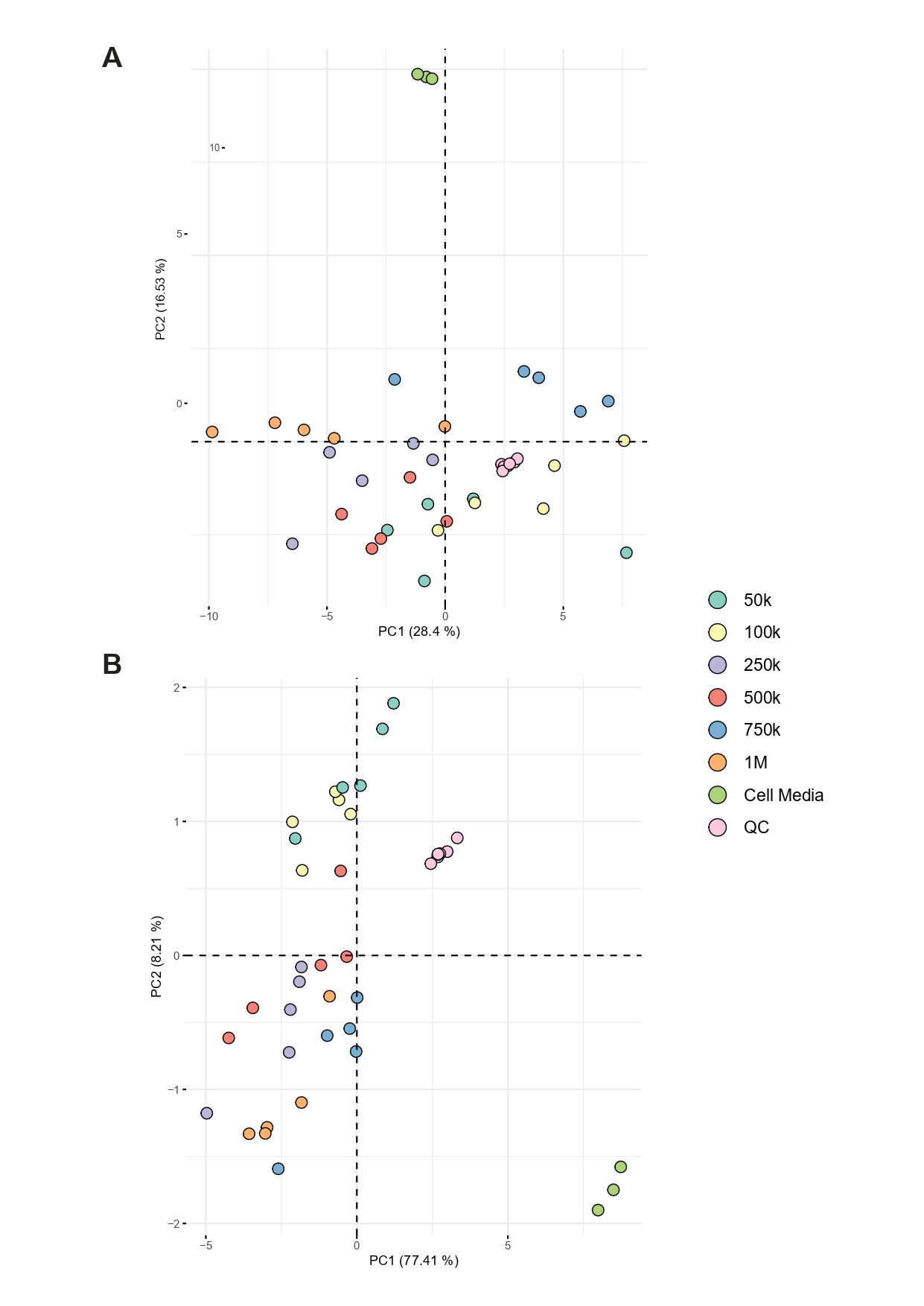


**Supplementary Figure 3:** **Correlated chemical signals allow an accurate grouping of samples by cell count in CD3+ cells model for ESI- mode. (A)** Heatmap with hierarchical clustering (HC) of all detected chemical signals (*k*=249). **(B)** Principal Component Analysis (PCA) model generated using all detected chemical signals (*k*=249). **(C)** Chord diagram showing the number of significant (*p* < 0.05) and positive (ρ > 0.7) correlations between chemical signal abundances and cell counts across defined cell count intervals. The connecting link thickness represents the number of correlated chemical signals (the greater the number, the thicker the line) in the interval defined by the 2 connected cell counts. Each cell count considered for the correlation analyses and with at least 1 significant correlation is represented. Darkest grey link highlights the widest cell count interval (50k-to-1M), which is selected for further analyses. **(D)** Heatmap with HC of chemical signals that were significantly and positively correlated with the cell count using data from the -to-interval (*k*=11). (**E**) PCA model of chemical signals that were significantly positively correlated with the cell count using data from the 50k-to-1M interval (*k*=11). For the CD3^+^ cohort, 5 biological replicates at 6 increasing cell counts were analyzed individually.


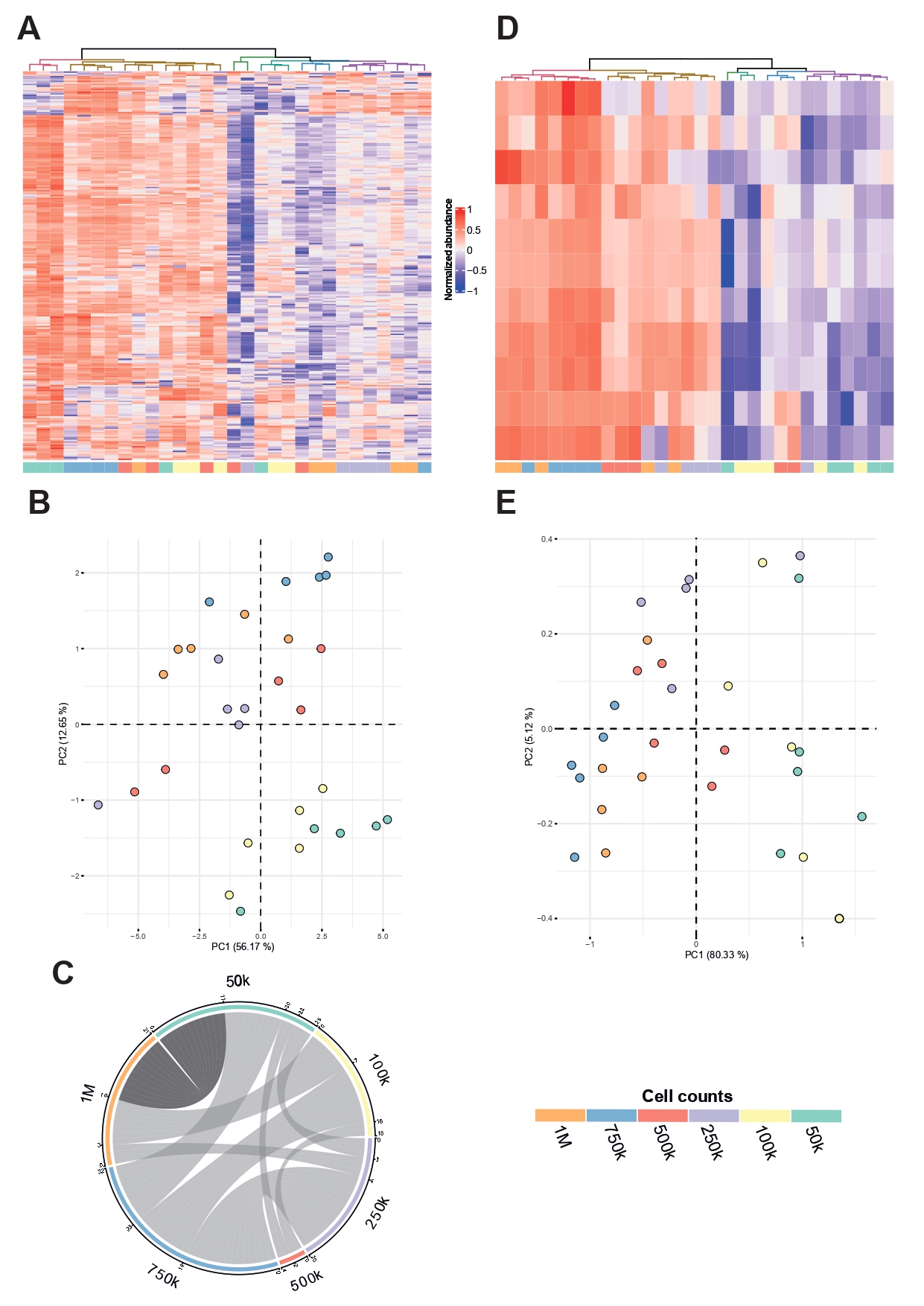


**Supplementary Figure 4: Cell count-dependent abundance of identified significant lipids resulting after applying correlation-based filtering in CD3^+^ cells.** Trajectories of the increasing abundances of representative glycerophosphocholines, sphingomyelins, and plasmalogen species as a function of the cell count are graphed. Spearman’s correlation coefficients (*ρ*) and associated *p*-values for the correlation are indicated for each lipid species (*ρ* > 0.7 and *p* < 0.05). Data are represented as mean ± SD. PC, Glycerophosphocholine; SM, Sphingomyelin; PC O/P (plasmalogens), Alkyl-/Alkenyl- Glycerophosphocholine.


**Supplementary Figure 5:** **PCA models generated using detected chemical signals after QA process in CD3+ model** **analyzed by ESI+ mode**, either with (A) the standard approach (*k*=1087) and (B) the correlation-based approach (*k*=70) after applying 3 types of normalization: median-, cell number- and TUS-based.

**Supplementary Figure 6: PCA models generated using detected chemical signals after QA process in CD3+ model analyzed by ESI- mode**, either with (A) the standard approach (*k*=249) and (B) the correlation-based approach (*k*=11) after applying 3 types of normalization: median-, cell number- and TUS-based.

**Supplementary Figure 7: Effect of data normalization on selected lipid subclasses.** The relative abundance of different lipid subclasses (glycerophosphocholines, sphingomyelins, and plasmalogens) was assessed under three normalization techniques, including median normalization, total useful signal (TUS) normalization, and cell number normalization). Data are represented as mean ± SD. PC, Glycerophosphocholine; SM, Sphingomyelin; PC O/P (plasmalogens), Alkyl-/Alkenyl- Glycerophosphocholine.


**Supplementary Figure 8: Histograms of missing values reported per chemical signal and per sample in the CD3+ cell model.** Histograms showing the number of chemical signals with a specific percentage of missing values in **(A)** ESI + and **(B)** ESI -. Bar plots showing the missing values percentage per sample (colored according to experimental groups) in **(C)** ESI + and **(D)** ESI -.

**Supplementary Figure 9: PCA models generated using all detected chemical signals after QA process (*k*=408 in ESI+ and *k*=580 in ESI-) in HMVEC-d cells** including cell media samples and quality controls (QC) for ESI+ (**A**) and ESI- (**B**) modes.

**
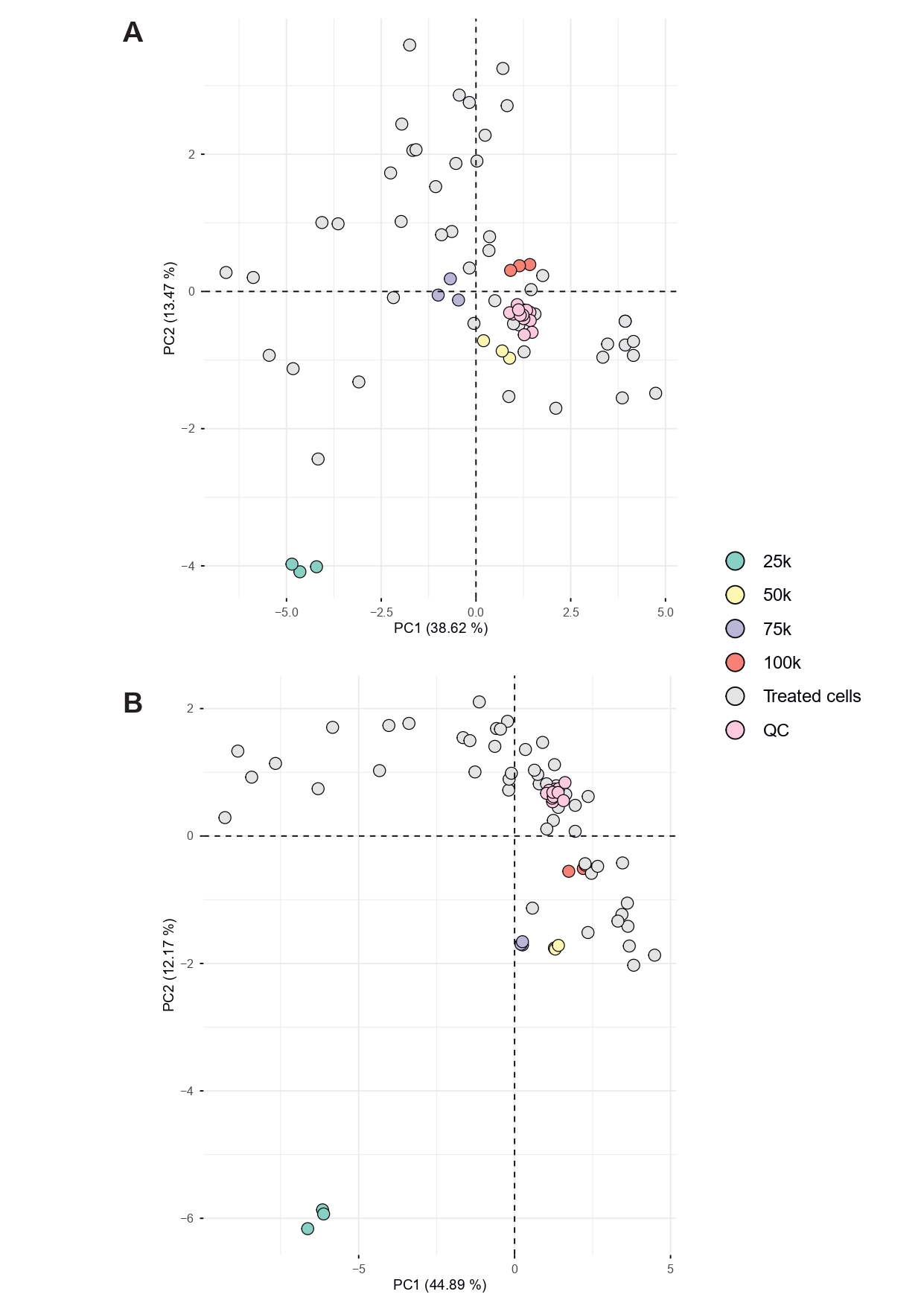
**

**Supplementary Figure 10: Correlated chemical signals allow an accurate grouping of samples by cell count in HMVEC-d model for ESI- mode. (A)** Heatmap with hierarchical clustering (HC) of all detected chemical signals (*k*=580). **(B)** Principal Component Analysis (PCA) model generated using all detected chemical signals (*k*=580). **(C)** Chord diagram showing the number of significant (*p* < 0.05) and positive (*ρ* > 0.7) correlations between chemical signal abundances and cell counts across defined cell count intervals. The connecting link thickness represents the number of correlated chemical signals (the greater the number, the thicker the line) in the interval defined by the 2 connected cell counts. Each cell count considered for the correlation analyses and with at least 1 significant correlation is represented. Darkest grey link highlights the widest cell count interval (25k-to-100k), which is selected for further analyses. **(D)** Heatmap with HC of chemical signals that were significantly and positively correlated with the cell count using data from the 25k-to-100k interval (*k*=276). (**E**) PCA model of chemical signals that were significantly positively correlated with the cell count using data from the 25k-to-100k interval (*k*=276). For the HMVEC-d cohort, the cell curve consisted of 4 increasing cell counts injected by triplicate.

**
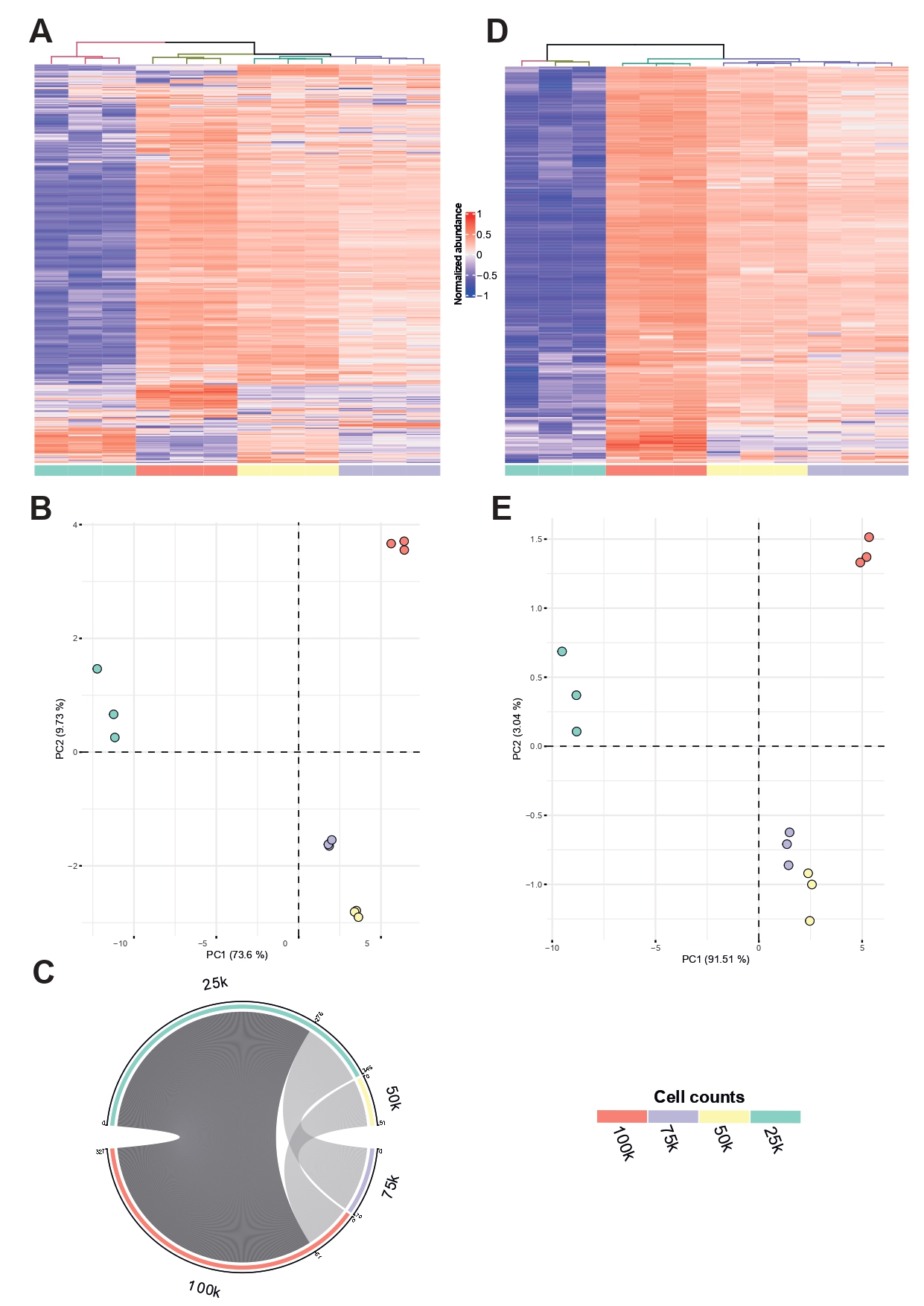
**

**Supplementary Figure 11: Behavior of the internal standards (ISTDs), added to HMVEC-d samples, throughout the chromatographic analysis.** Non-endogenous ISTD abundances were monitored in positive (A, ESI+) and negative (B, ESI-) ionization modes -depending on the preferred ionization mode of each lipid- for QC samples (orange points) and cell curves (blue points). Coefficient of variation (%CV) was represented and calculated for the QC samples (marked in orange) and for the cell curve (marked in blue). PC, Glycerophosphocholines; PE, Glycerophosphoethanolamine; DG, Diacylglycerol; PI, Glycerophosphoinositols; Cer, Ceramide.


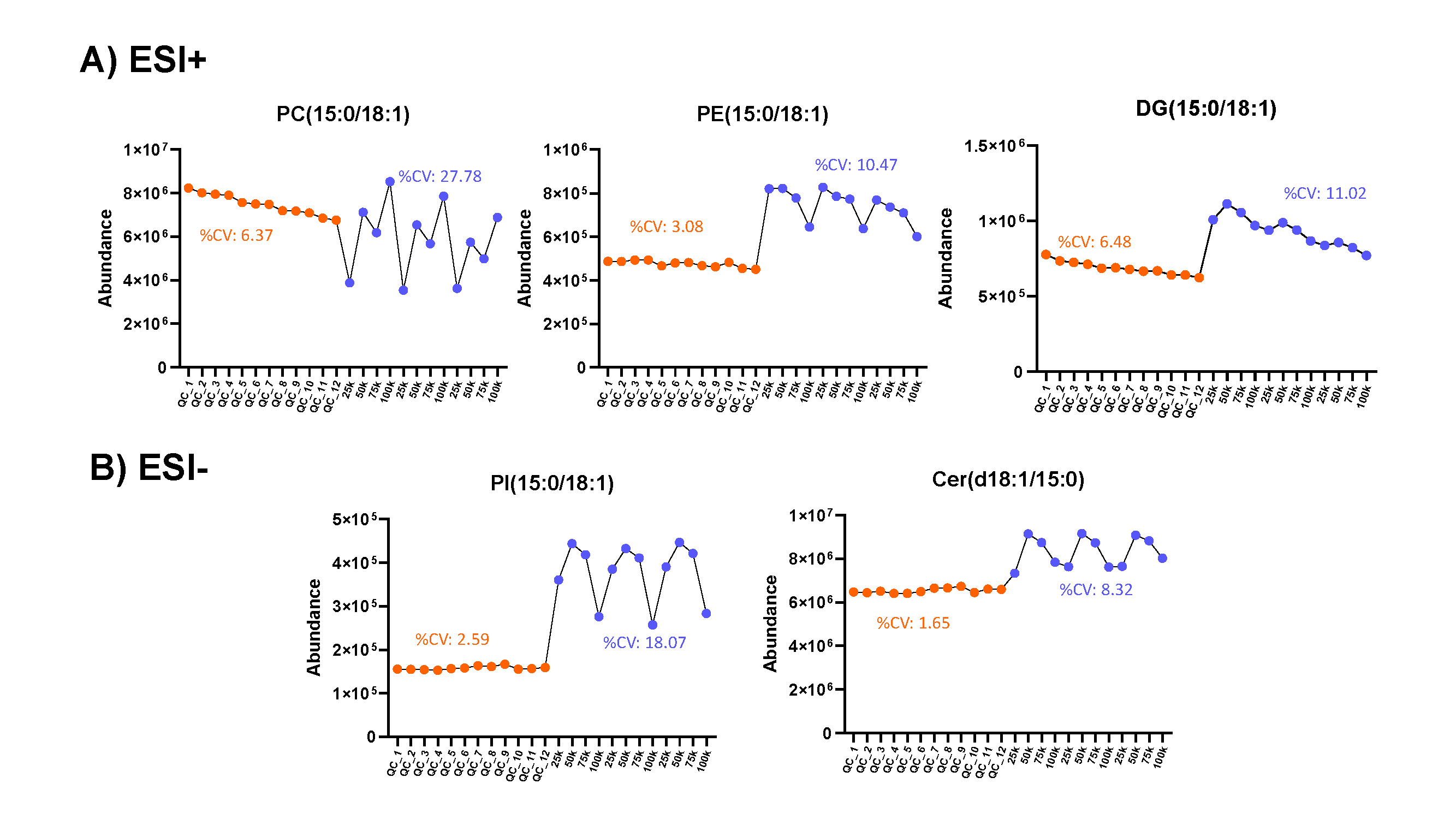


**Supplementary Figure 12: Comparison of PCA models built using chemical signals obtained applying the standard approach and the correlation-based approach for CD3^+^ and HMVEC-d models in ESI+ and ESI- modes.** Standard approach: (A) CD3^+^, ESI +, *k* = 1087; (C) CD3^+^, ESI -, *k* = 249; (E) HMVEC-d, ESI+, *k* = 408; and (G) HMVEC-d, ESI-, *k* = 580. Correlation based-approach: (B) CD3^+^, ESI +, *k* = 70; (D) CD3^+^, ESI -, *k* = 11; (F) HMVEC-d, ESI+, *k* =157; and (H) HMVEC-d, ESI-, *k* = 276.
