# Supplementary figures and images for: Establishing an untargeted lipidomics workflow for cellular analysis: insights into endothelial cell function in anaphylaxis
Source: Front Immunol. 2026 Mar 4;17:1711640. doi: 10.3389/fimmu.2026.1711640 (PMC12997047; doi:10.3389/fimmu.2026.1711640)

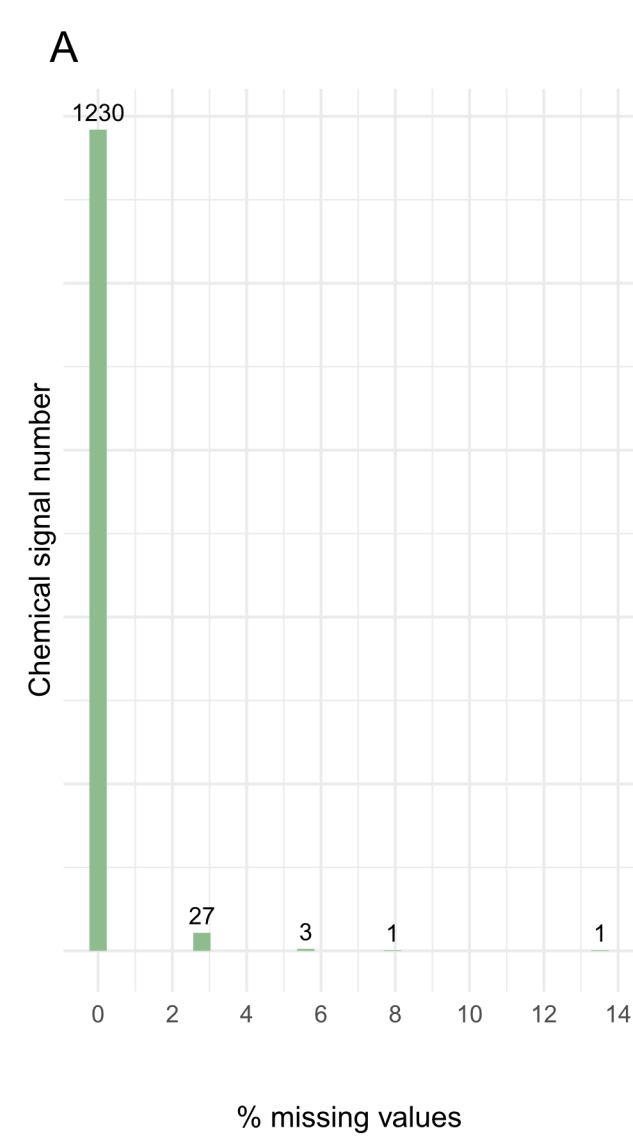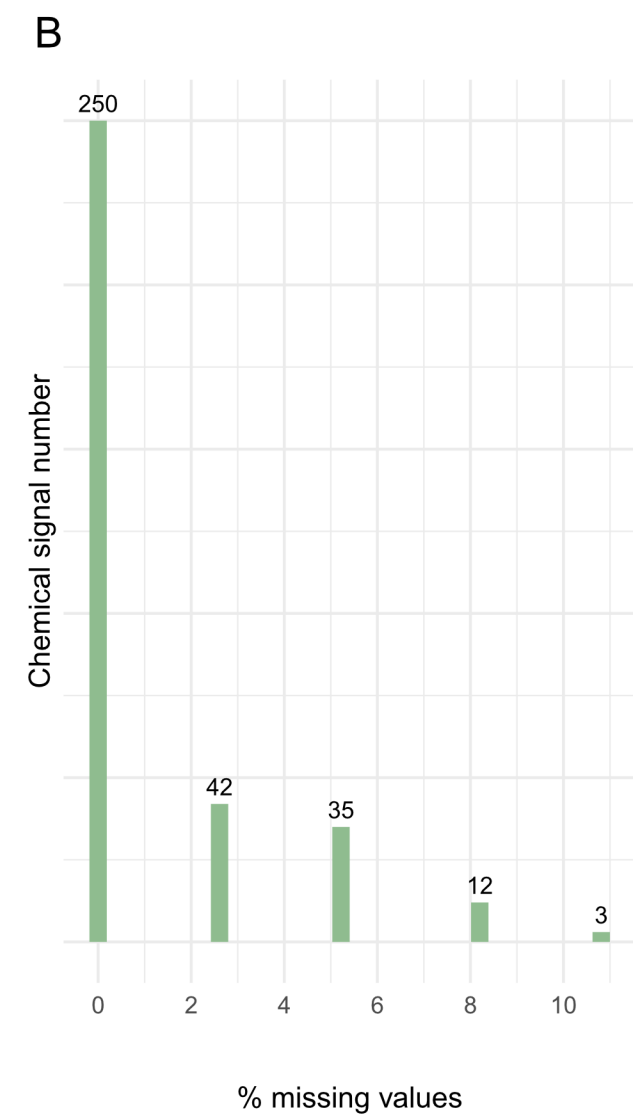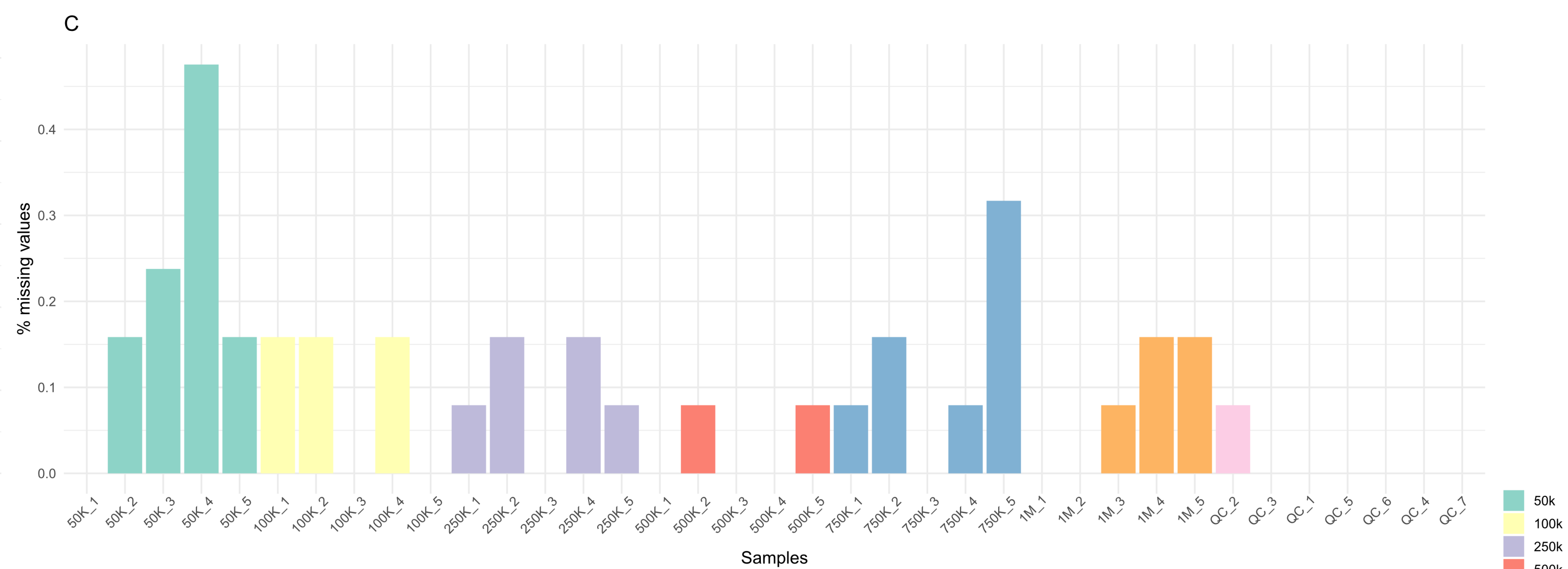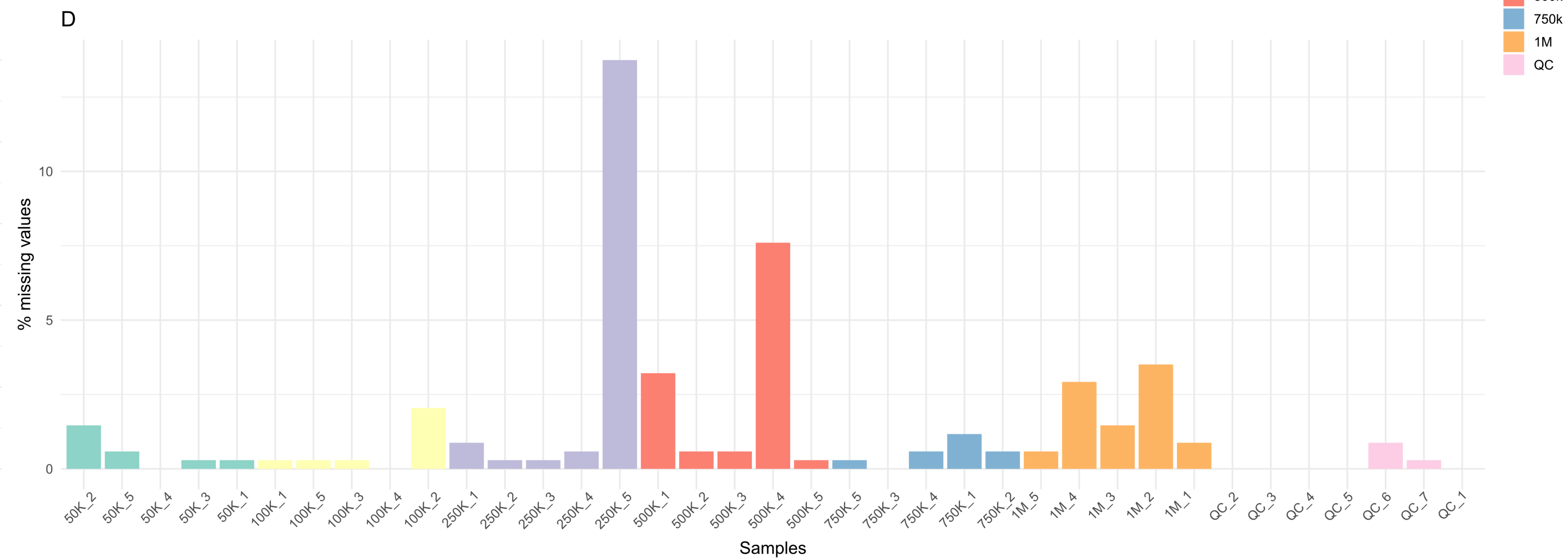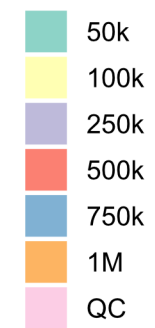

Supplement: Supplementary file 2 [file Image1.pdf]

**A**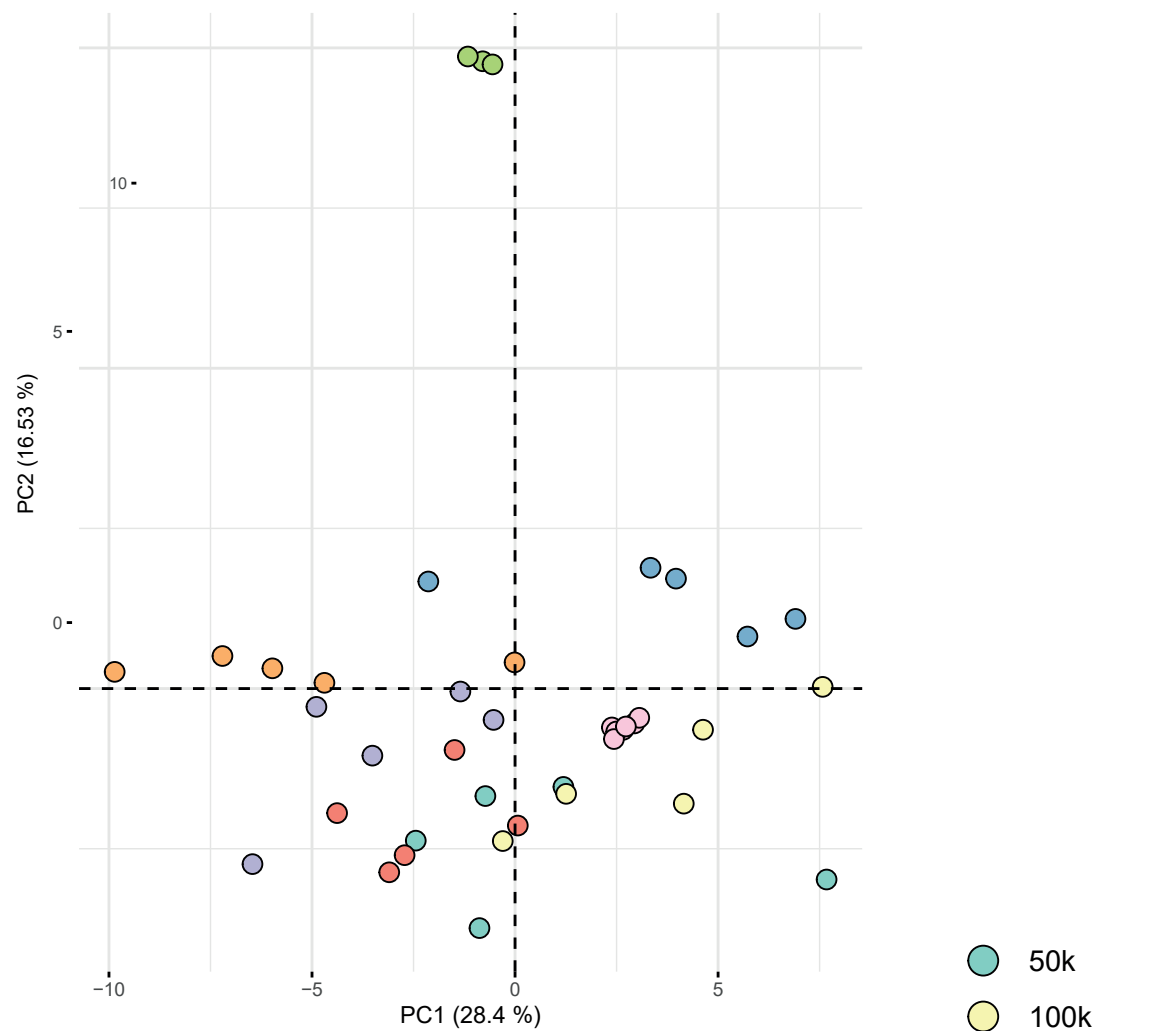**B**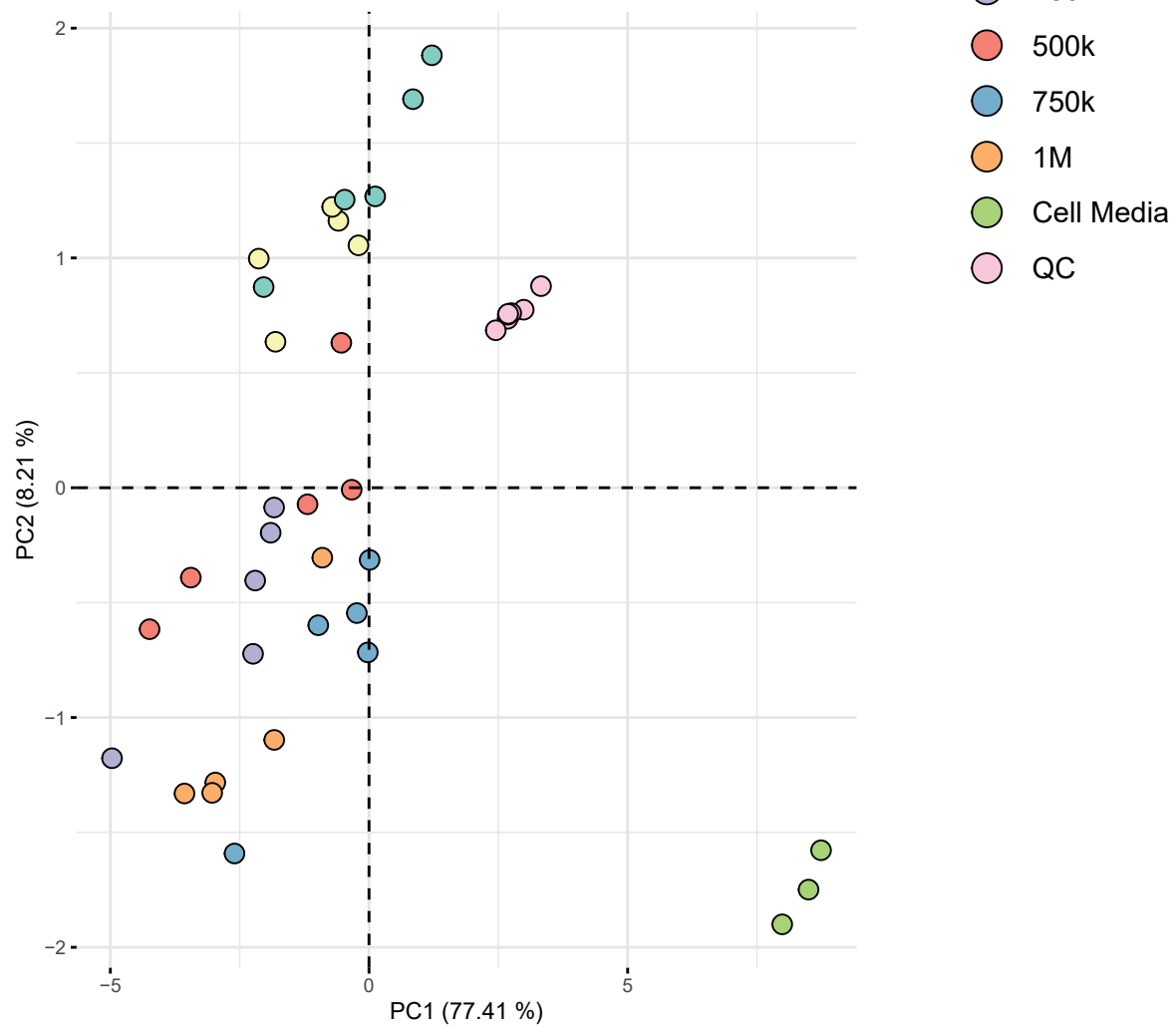

Supplement: Supplementary file 3 [file Image2.pdf]

**A**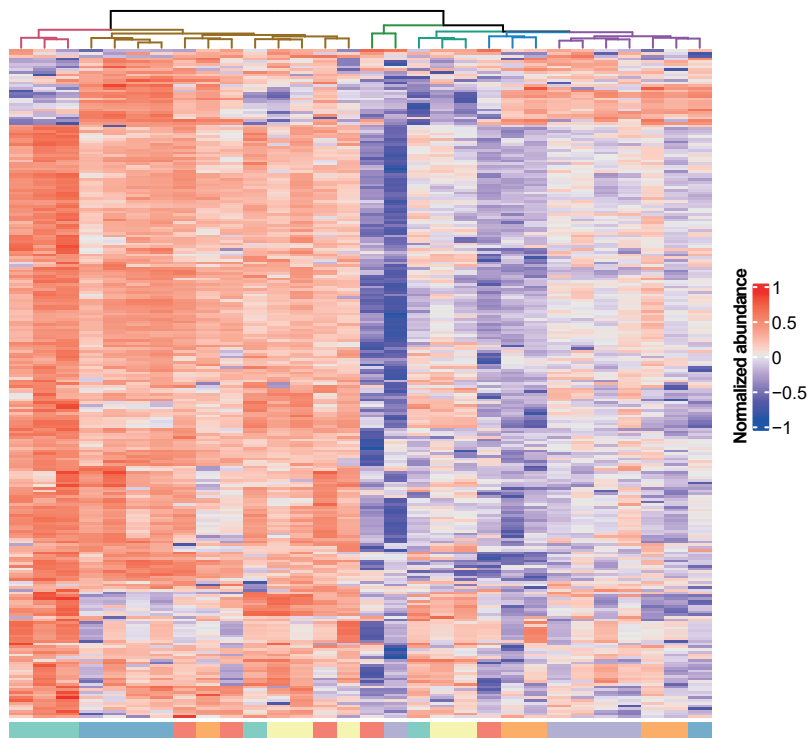**D**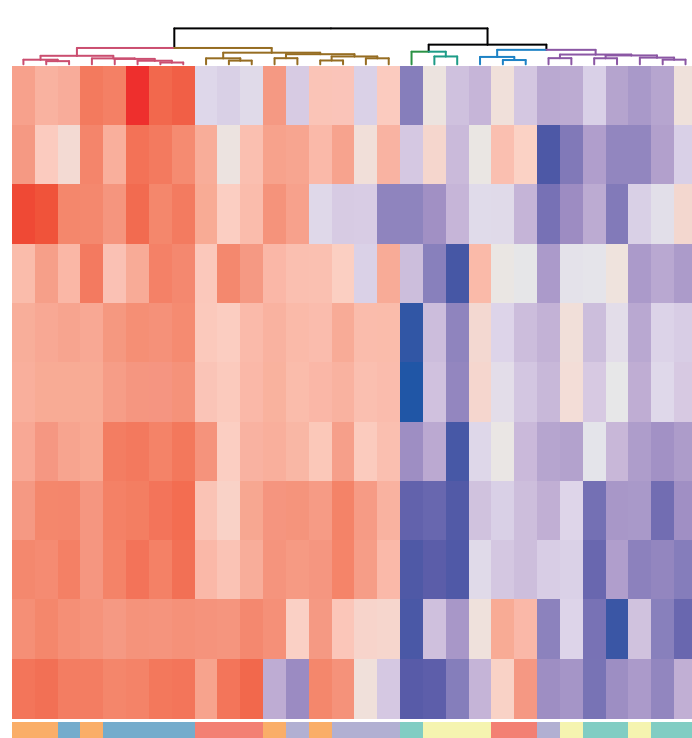**B**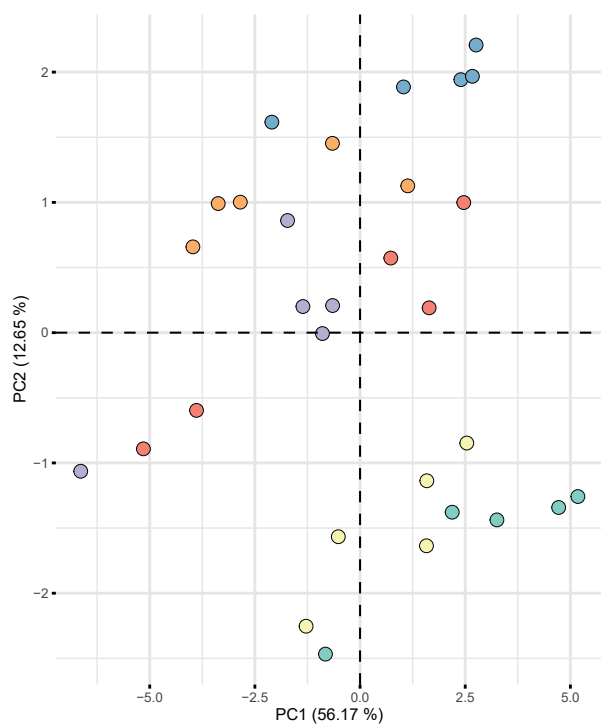**E**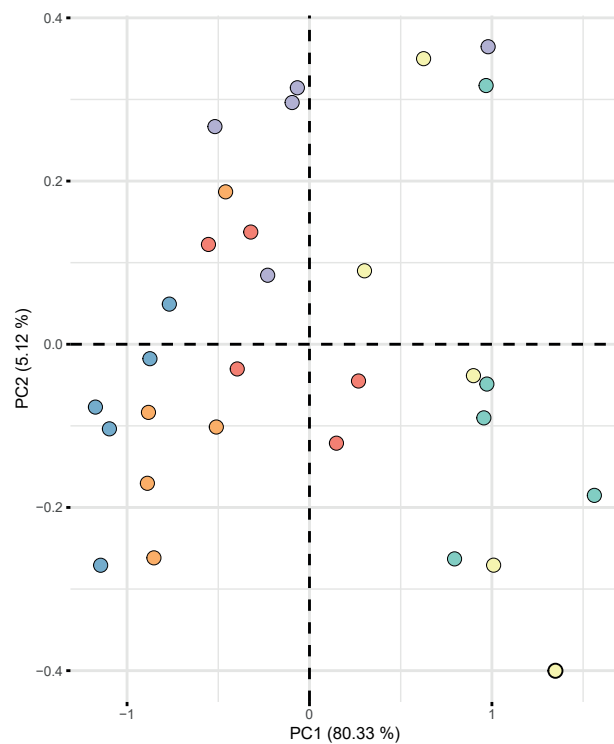**C**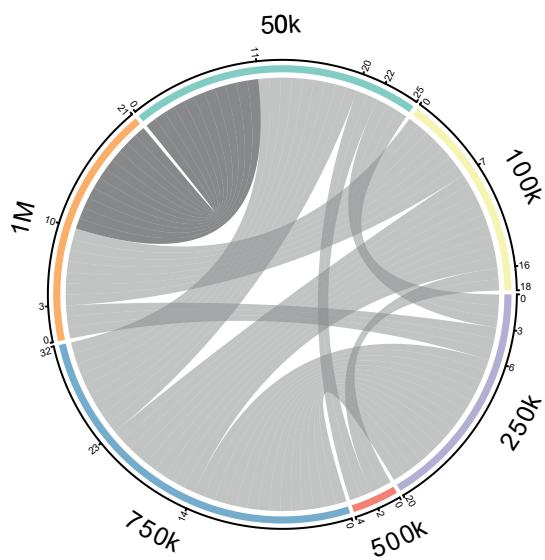**Cell counts**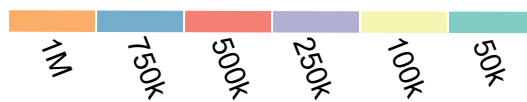

Supplement: Supplementary file 4 [file Image3.pdf]

## Glycerophosphocholines

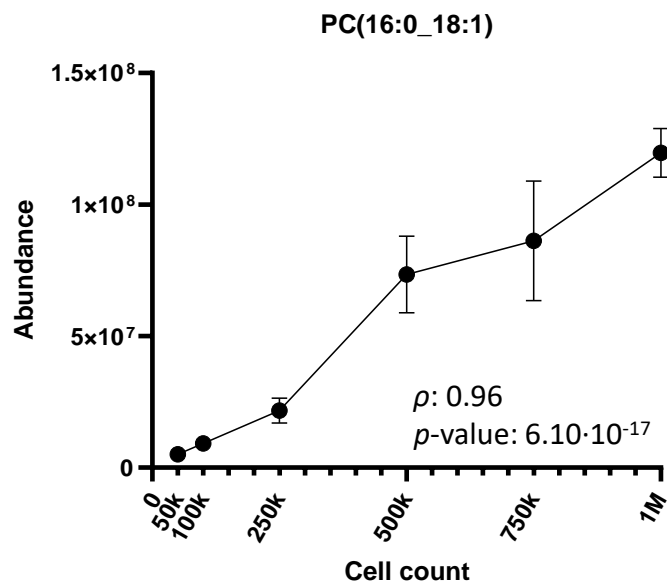

## Sphingomyelins

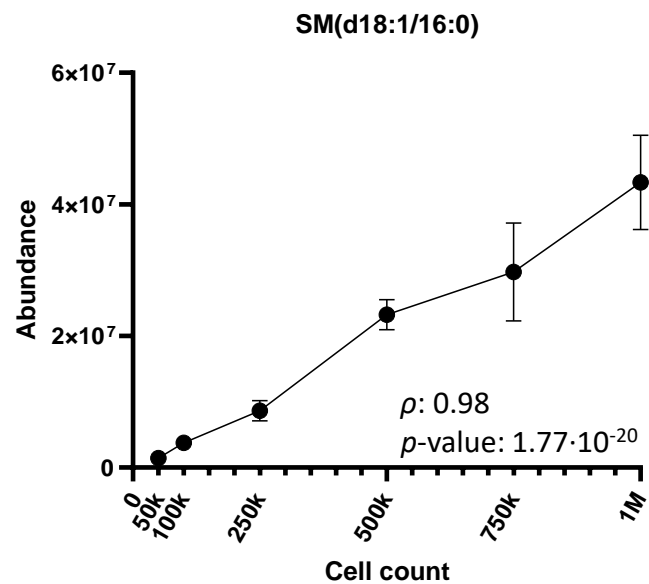

## Plasmalogens

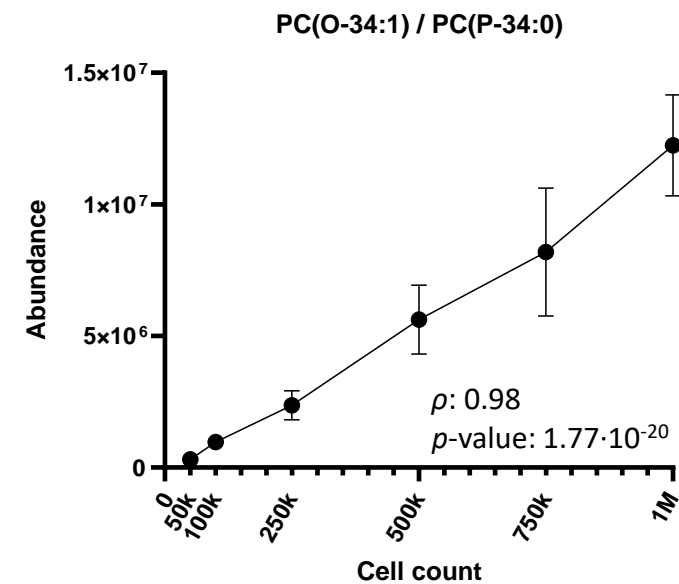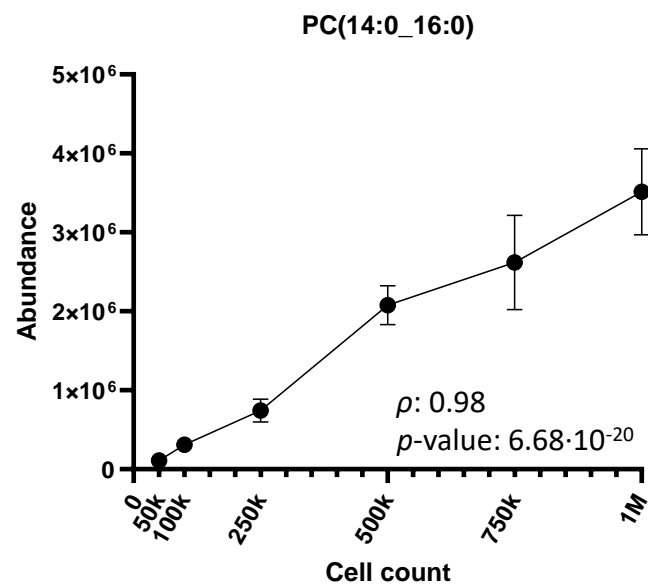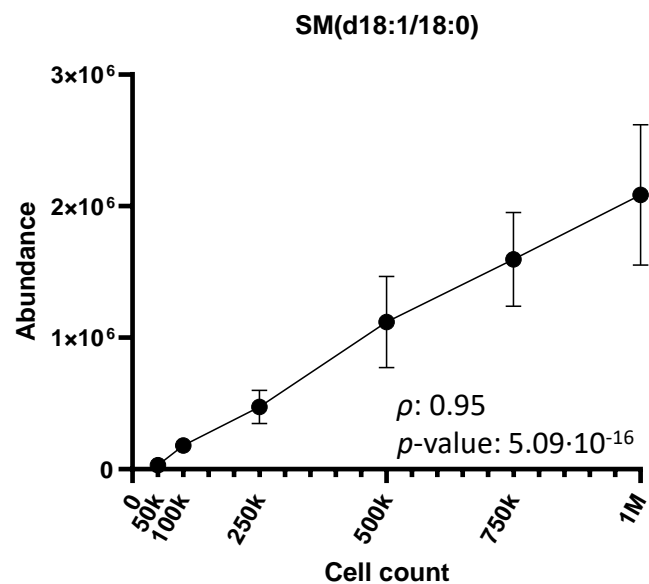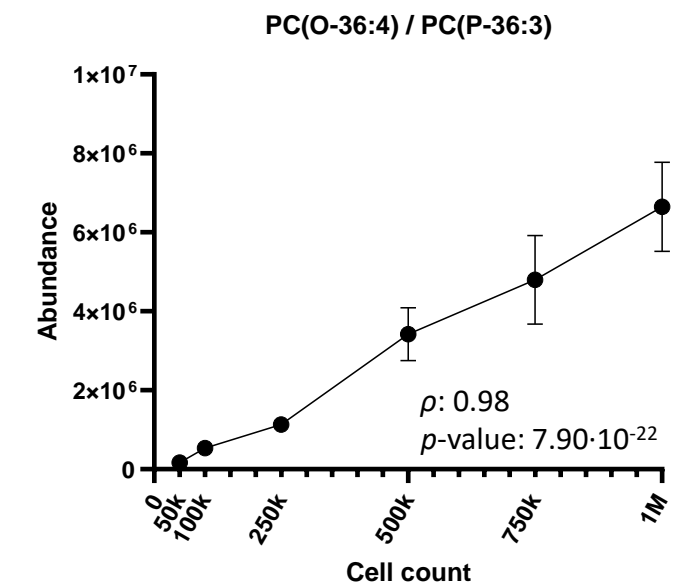

Supplement: Supplementary file 5 [file Image4.pdf]

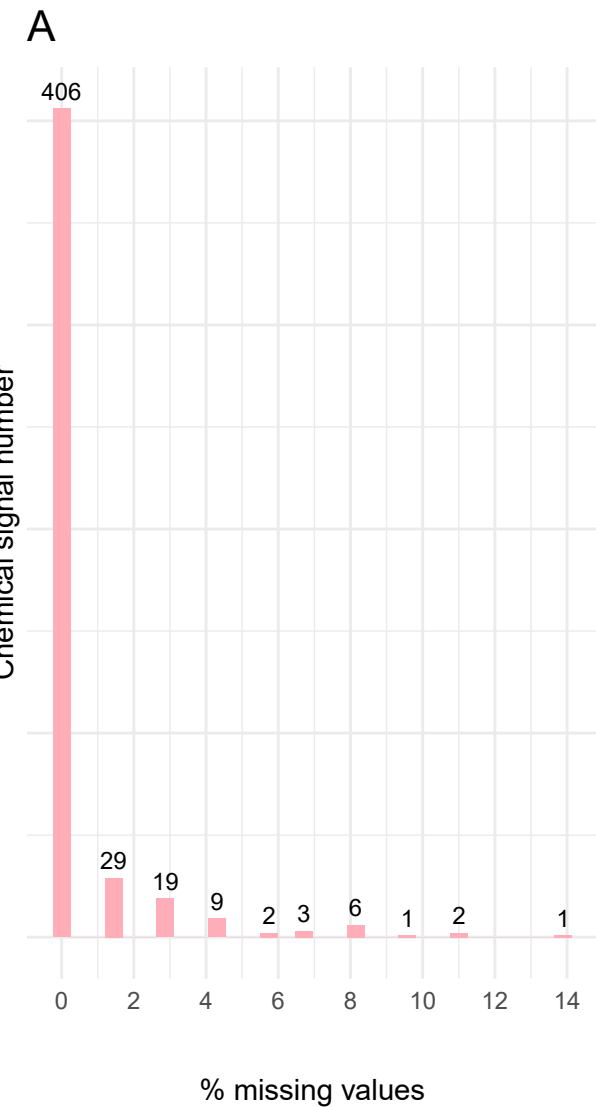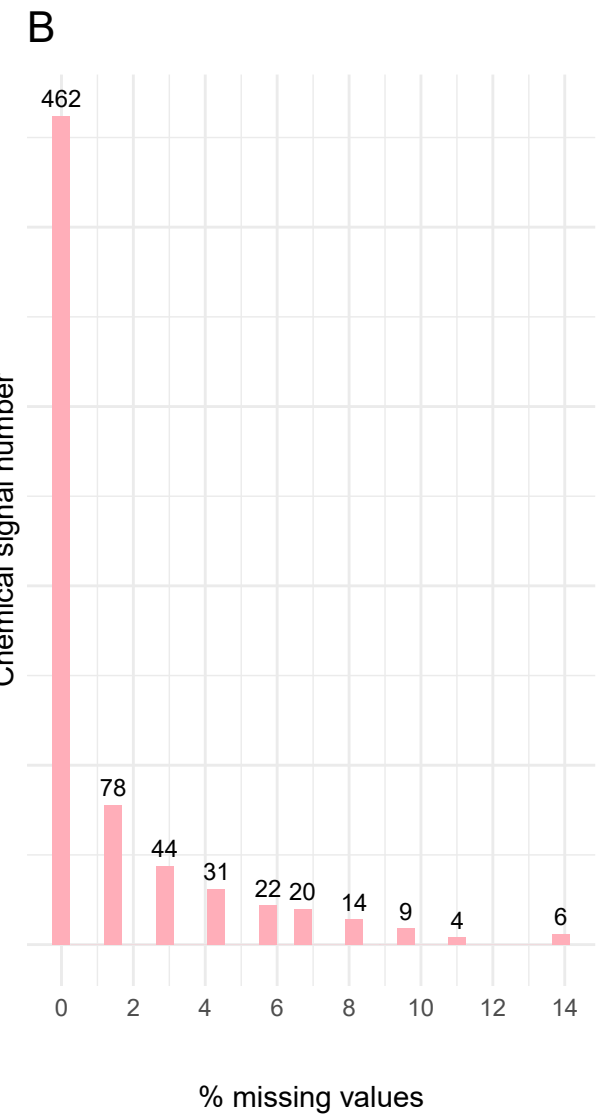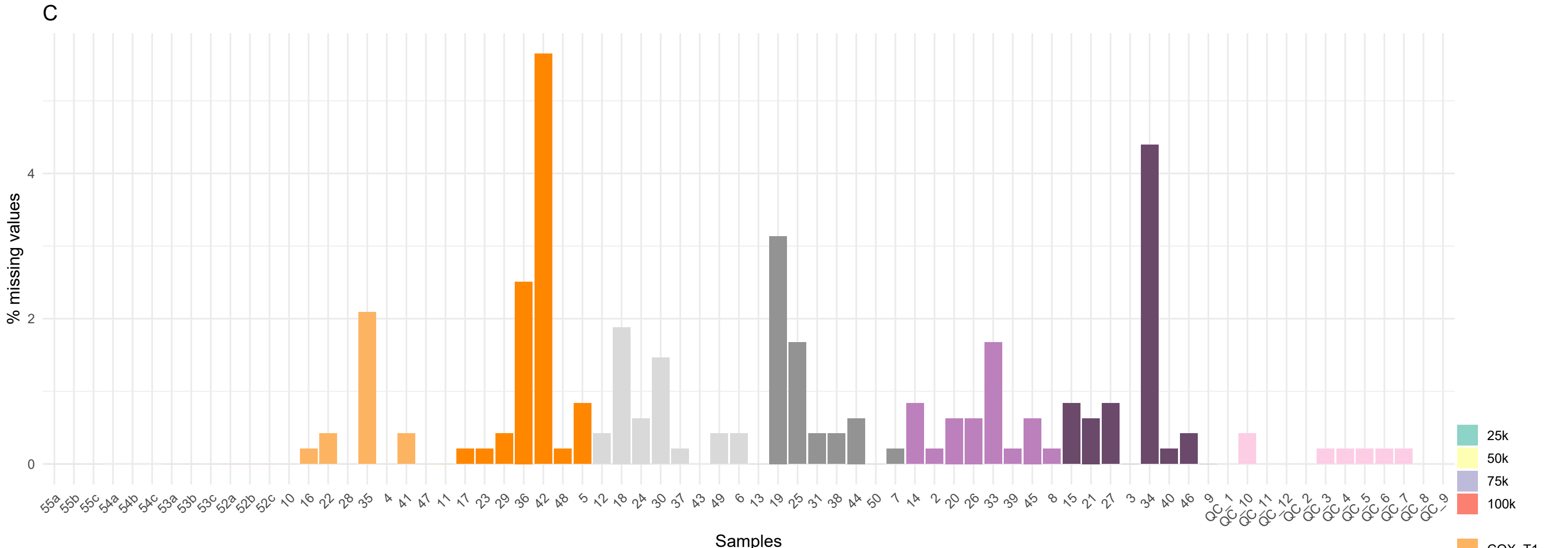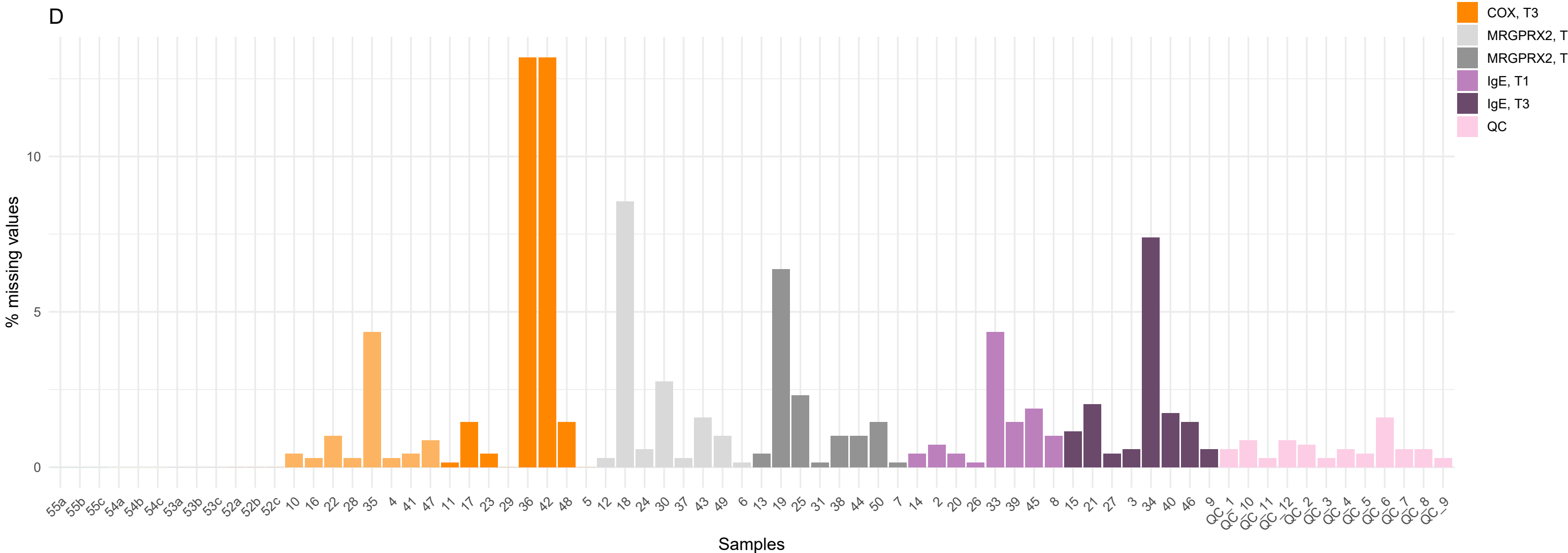

- 25k
- 50k
- 75k
- 100k
- COX, T1
- COX, T3
- MRGPRX2, T1
- MRGPRX2, T3
- IgE, T1
- IgE, T3
- QC

Supplement: Supplementary file 9 [file Image8.pdf]

**A**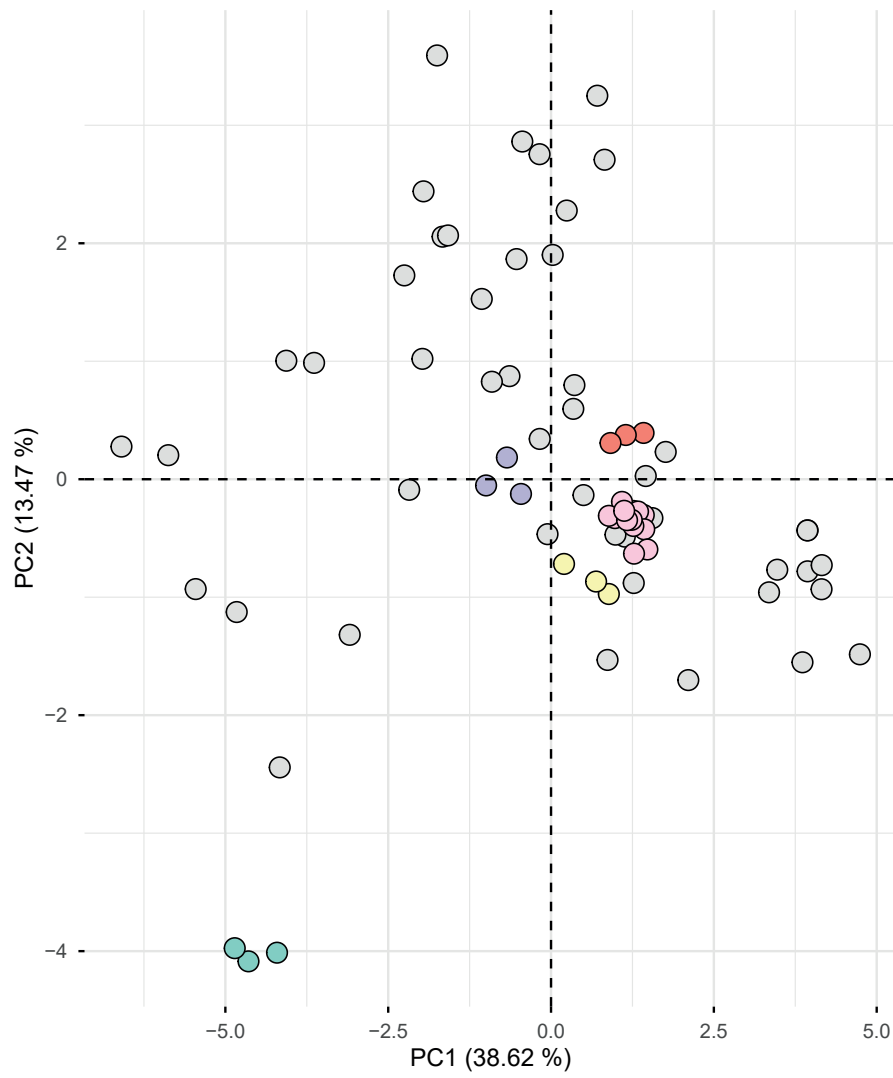**B**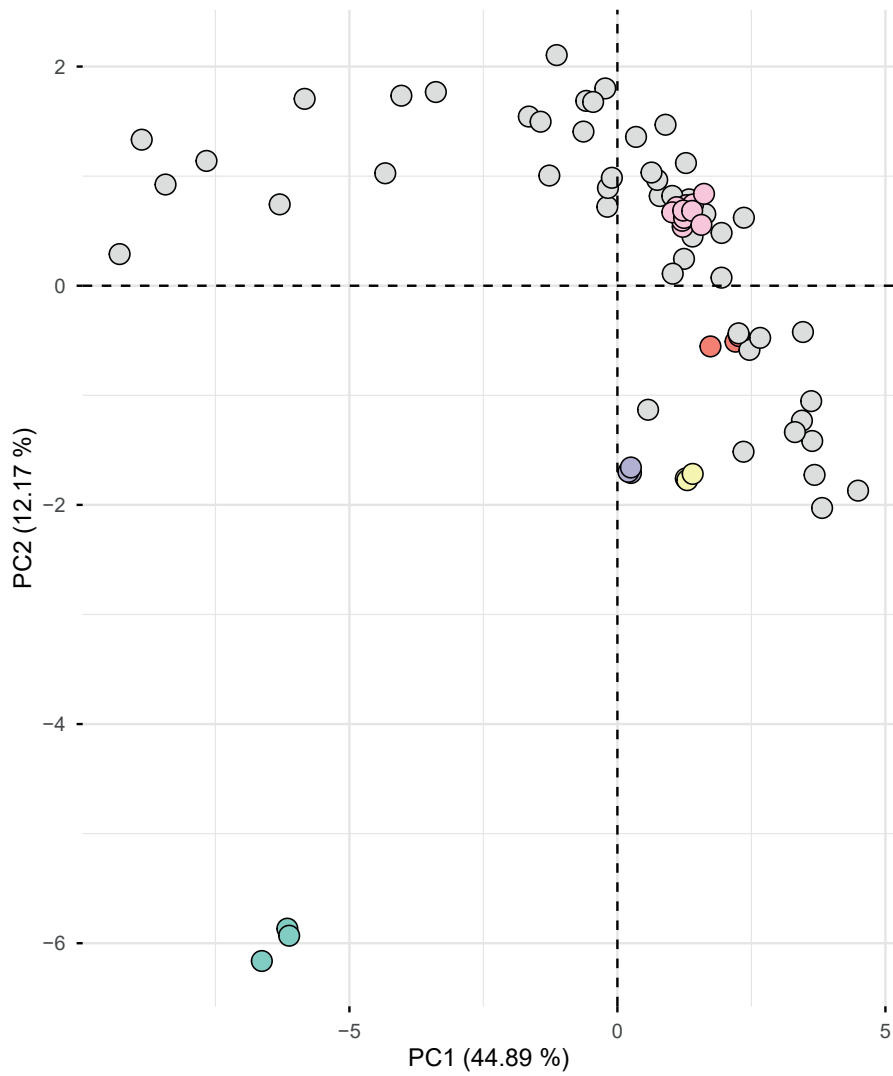

Supplement: Supplementary file 10 [file Image9.pdf]

**A**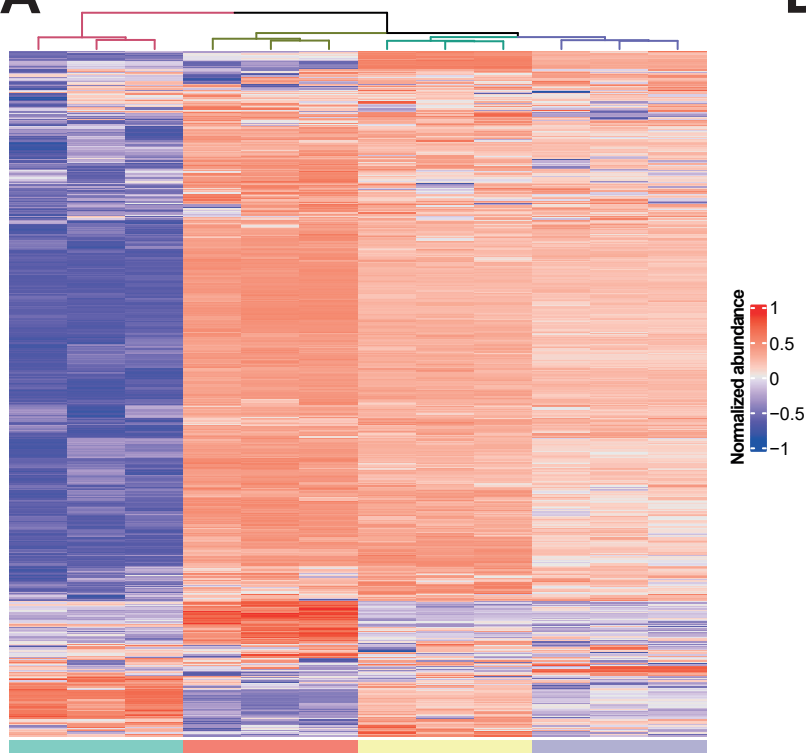**D**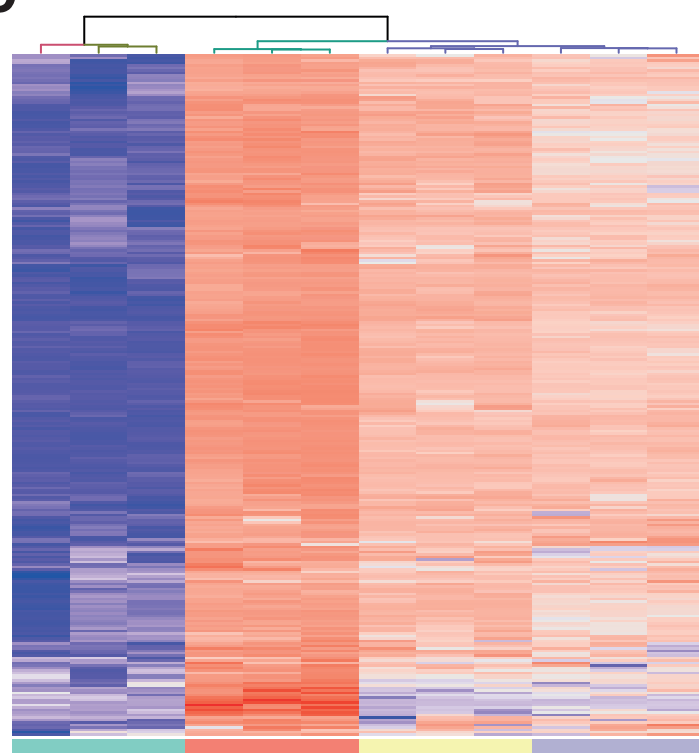**B**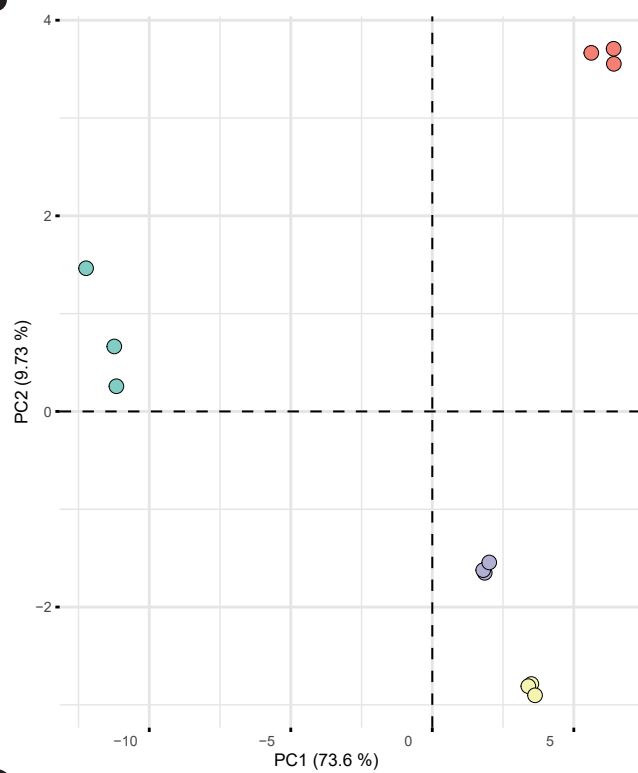**E**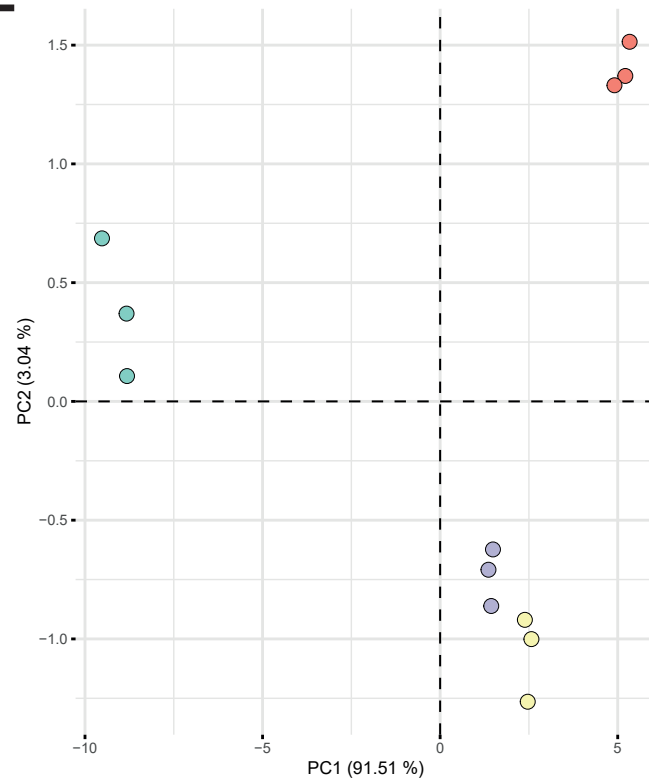**C**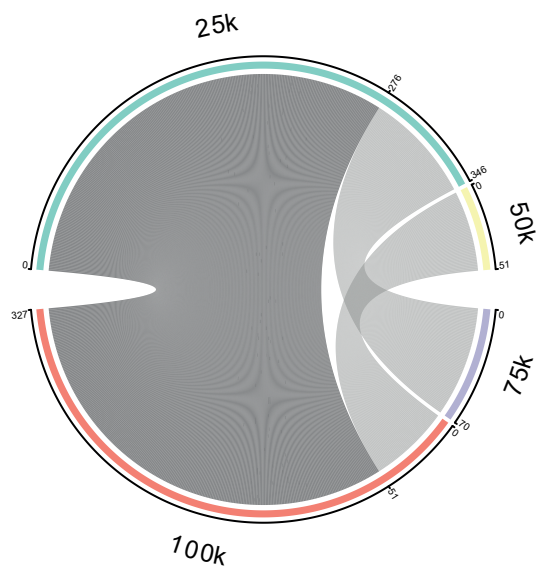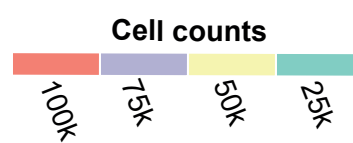

Supplement: Supplementary file 11 [file Image10.pdf]

A) ESI+

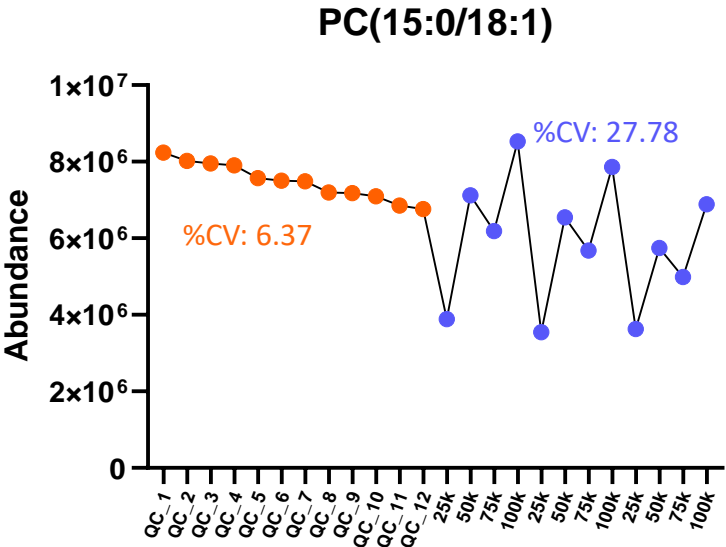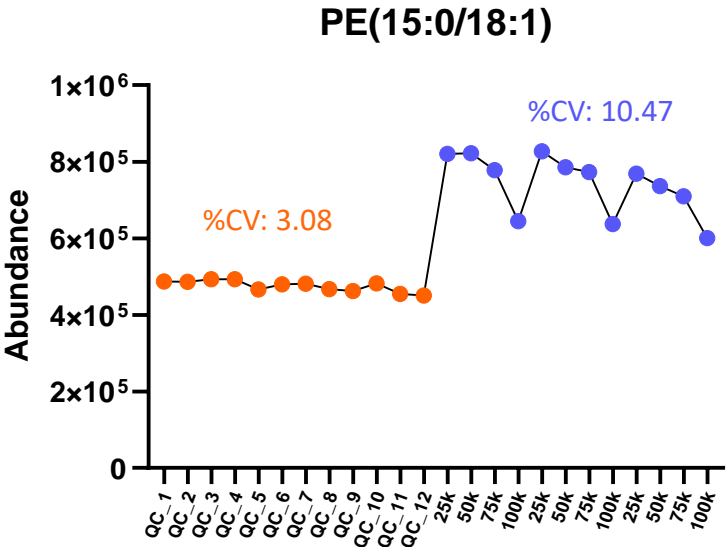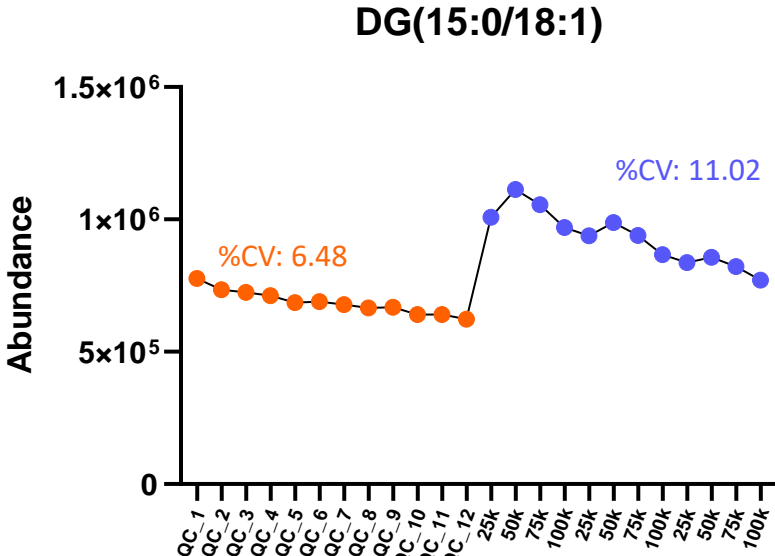

B) ESI-

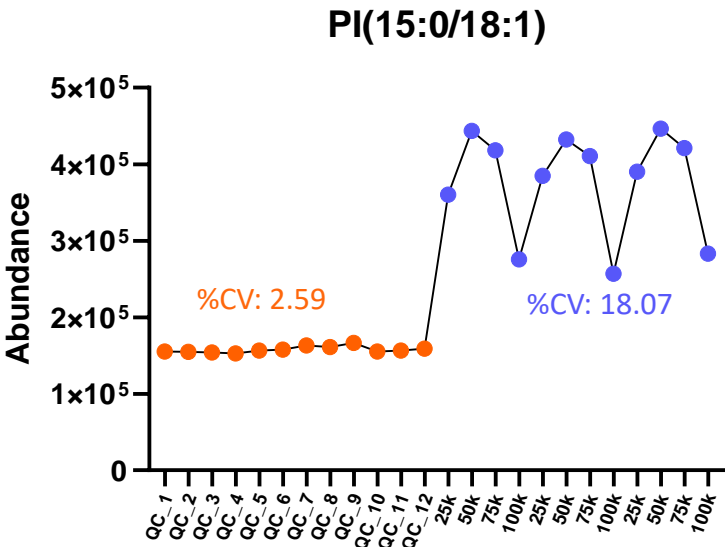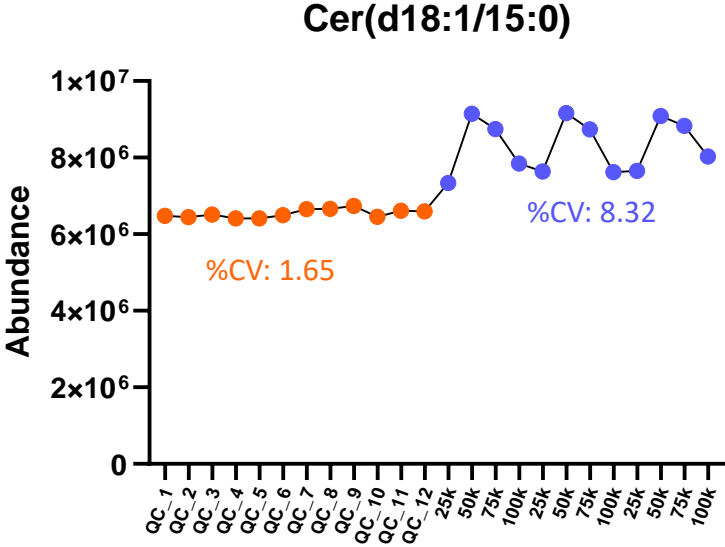

Supplement: Supplementary file 12 [file Image11.pdf]
