# Supplementary material for: Establishing an untargeted lipidomics workflow for cellular analysis: insights into endothelial cell function in anaphylaxis
Source: Front Immunol. 2026 Mar 4;17:1711640. doi: 10.3389/fimmu.2026.1711640 (PMC12997047; doi:10.3389/fimmu.2026.1711640)
Supplement: Supplementary file 6 [file Image5.pdf]

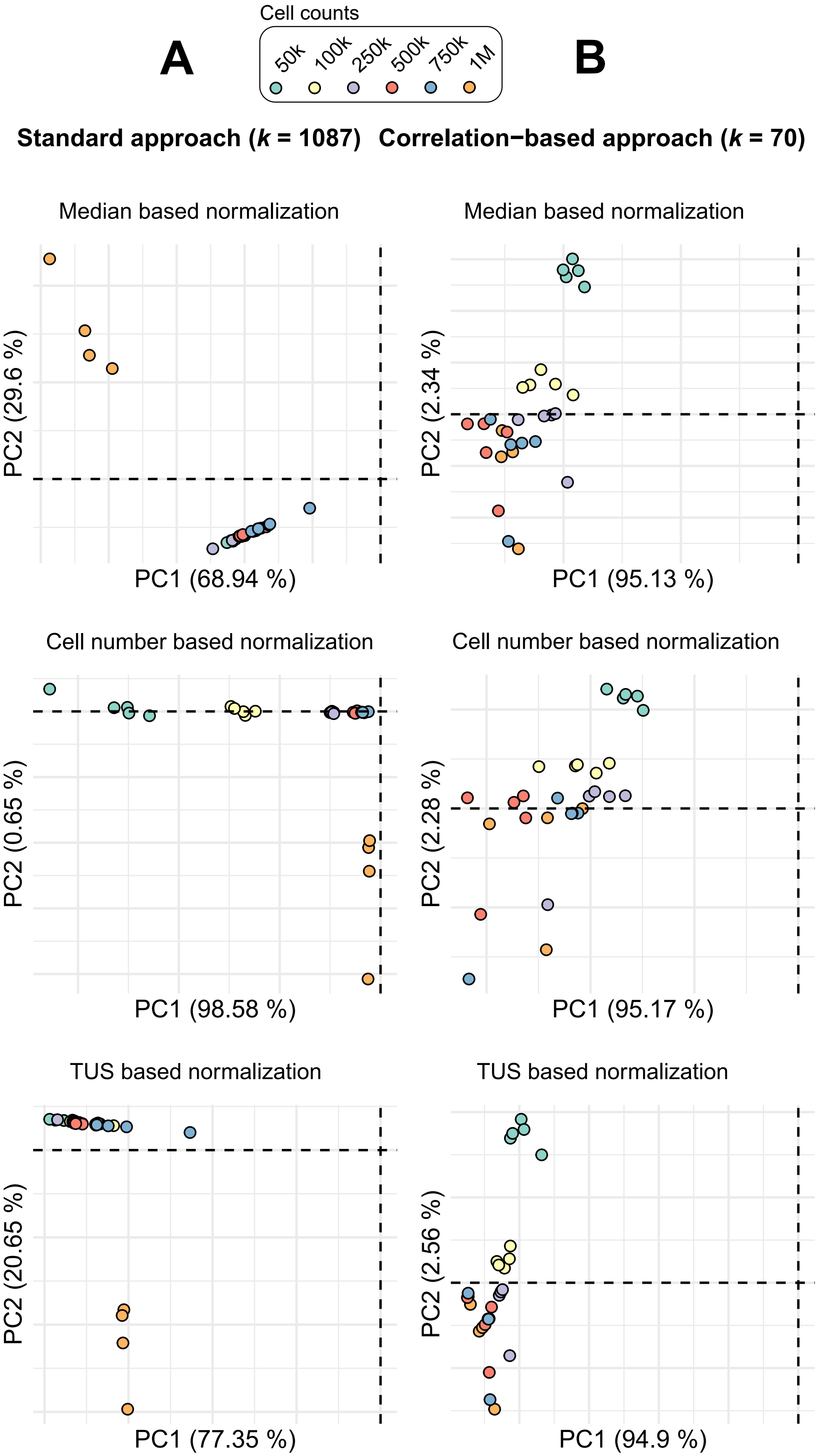
A
B
Cell counts
50k
100k
250k
500k
750k
1M
Standard approach (*k* = 1087)
Correlation−based approach (*k* = 70)
Median based normalization
PC2 (29.6 %)
PC1 (68.94 %)
Median based normalization
PC2 (2.34 %)
PC1 (95.13 %)
Cell number based normalization
PC2 (0.65 %)
PC1 (98.58 %)
Cell number based normalization
PC2 (2.28 %)
PC1 (95.17 %)
TUS based normalization
PC2 (20.65 %)
PC1 (77.35 %)
TUS based normalization
PC2 (2.56 %)
PC1 (94.9 %)
