# Supplementary material for: Establishing an untargeted lipidomics workflow for cellular analysis: insights into endothelial cell function in anaphylaxis
Source: Front Immunol. 2026 Mar 4;17:1711640. doi: 10.3389/fimmu.2026.1711640 (PMC12997047; doi:10.3389/fimmu.2026.1711640)
Supplement: Supplementary file 7 [file Image6.pdf]

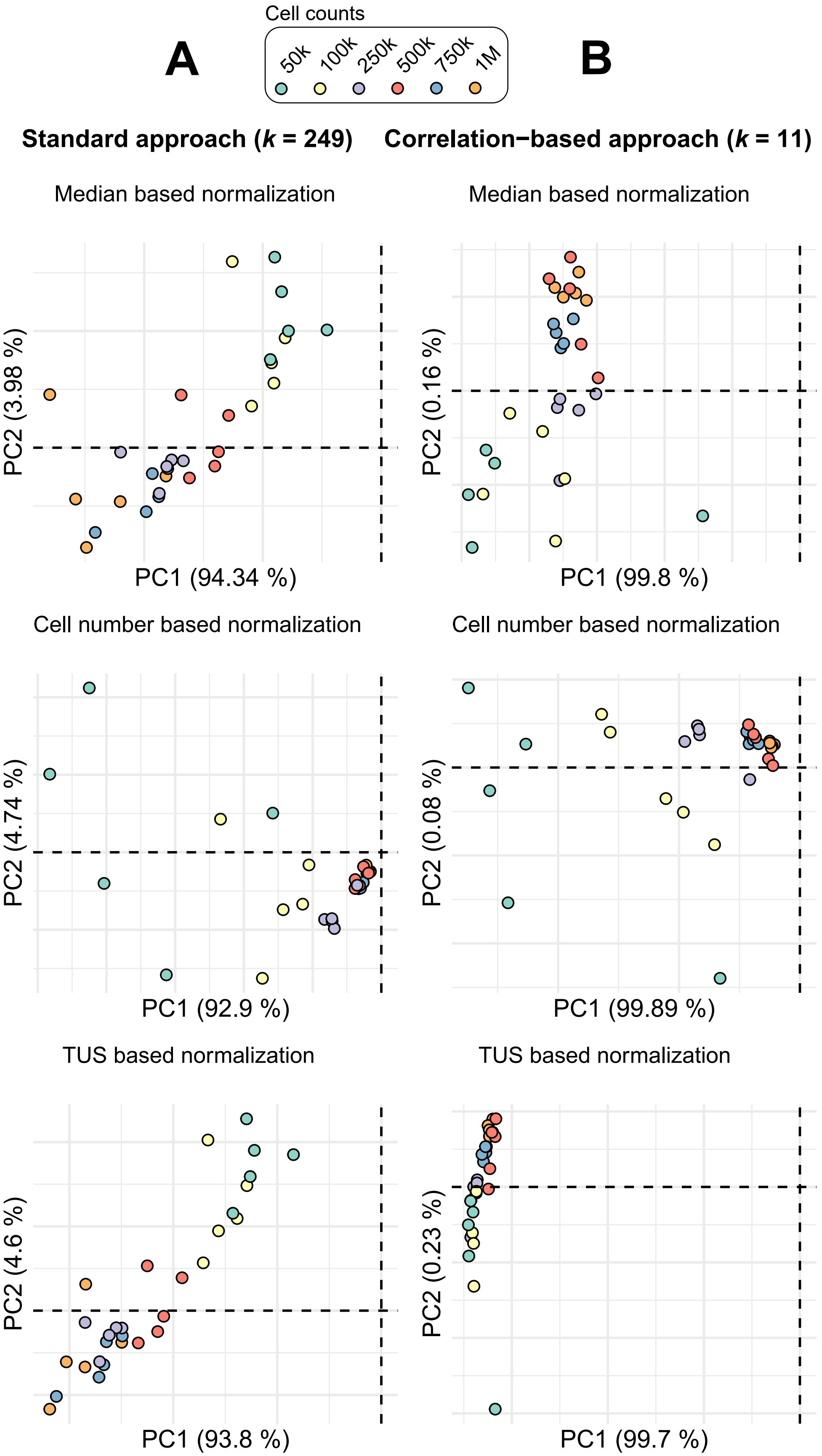
Cell counts
50k
100k
250k
500k
750k
1M
A
B
Standard approach (k = 249)
Correlation−based approach (k = 11)
Median based normalization
PC2 (3.98 %)
PC1 (94.34 %)
Median based normalization
PC2 (0.16 %)
PC1 (99.8 %)
Cell number based normalization
PC2 (4.74 %)
PC1 (92.9 %)
Cell number based normalization
PC2 (0.08 %)
PC1 (99.89 %)
TUS based normalization
PC2 (4.6 %)
PC1 (93.8 %)
TUS based normalization
PC2 (0.23 %)
PC1 (99.7 %)
