# Supplementary material for: Establishing an untargeted lipidomics workflow for cellular analysis: insights into endothelial cell function in anaphylaxis
Source: Front Immunol. 2026 Mar 4;17:1711640. doi: 10.3389/fimmu.2026.1711640 (PMC12997047; doi:10.3389/fimmu.2026.1711640)
Supplement: Supplementary file 8 [file Image7.pdf]

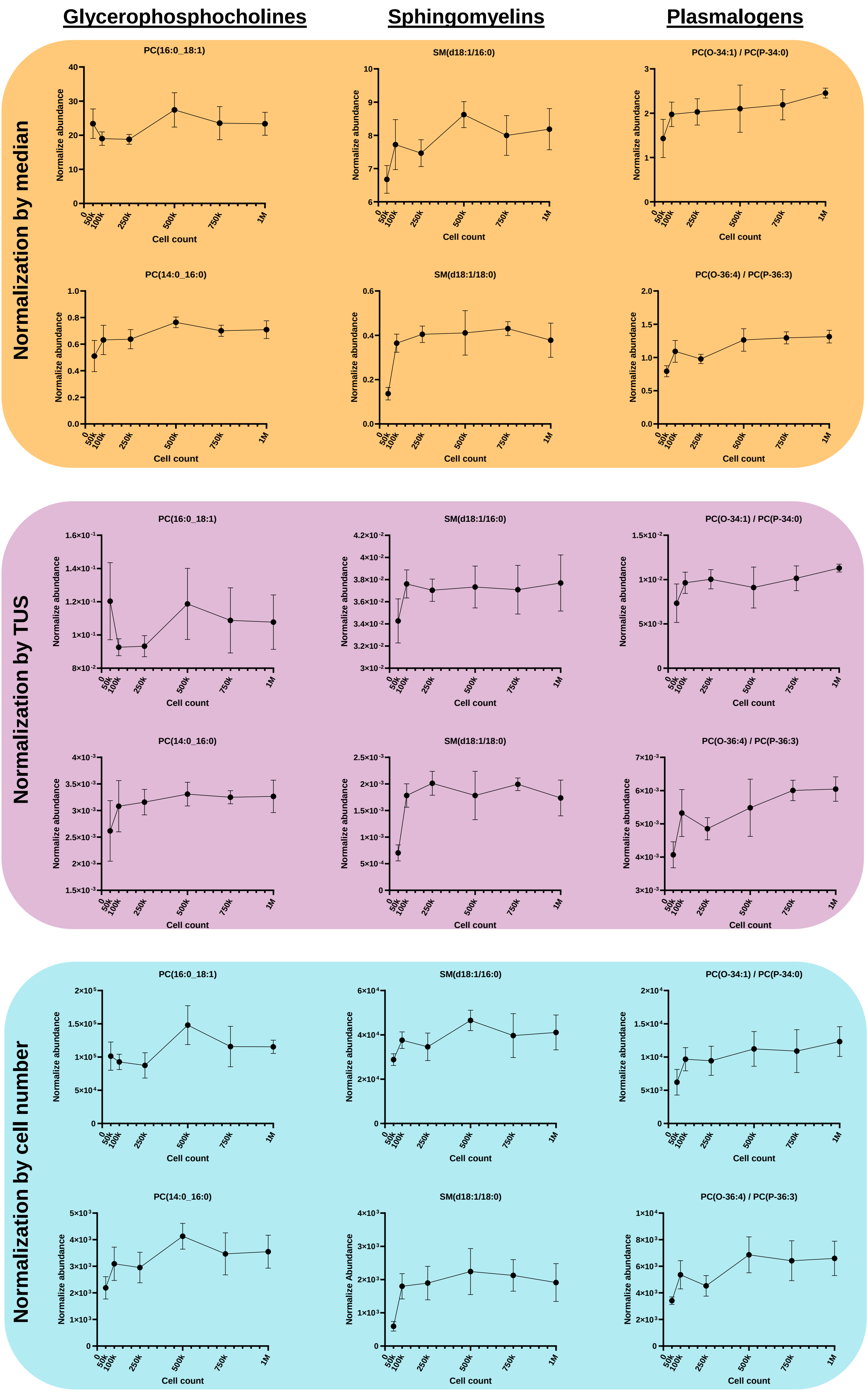

Glycerophosphocholines
Sphingomyelins
Plasmalogens
Normalization by median
Normalization by TUS
Normalization by cell number
PC(16:0_18:1)
PC(14:0_16:0)
SM(d18:1/16:0)
SM(d18:1/18:0)
PC(O-34:1) / PC(P-34:0)
PC(O-36:4) / PC(P-36:3)
Normalize abundance
Normalize Abundance
Cell count
0
50k
100k
250k
500k
750k
1M
