# Supplementary material for: Establishing an untargeted lipidomics workflow for cellular analysis: insights into endothelial cell function in anaphylaxis
Source: Front Immunol. 2026 Mar 4;17:1711640. doi: 10.3389/fimmu.2026.1711640 (PMC12997047; doi:10.3389/fimmu.2026.1711640)
Supplement: Supplementary file 13 [file Image12.pdf]

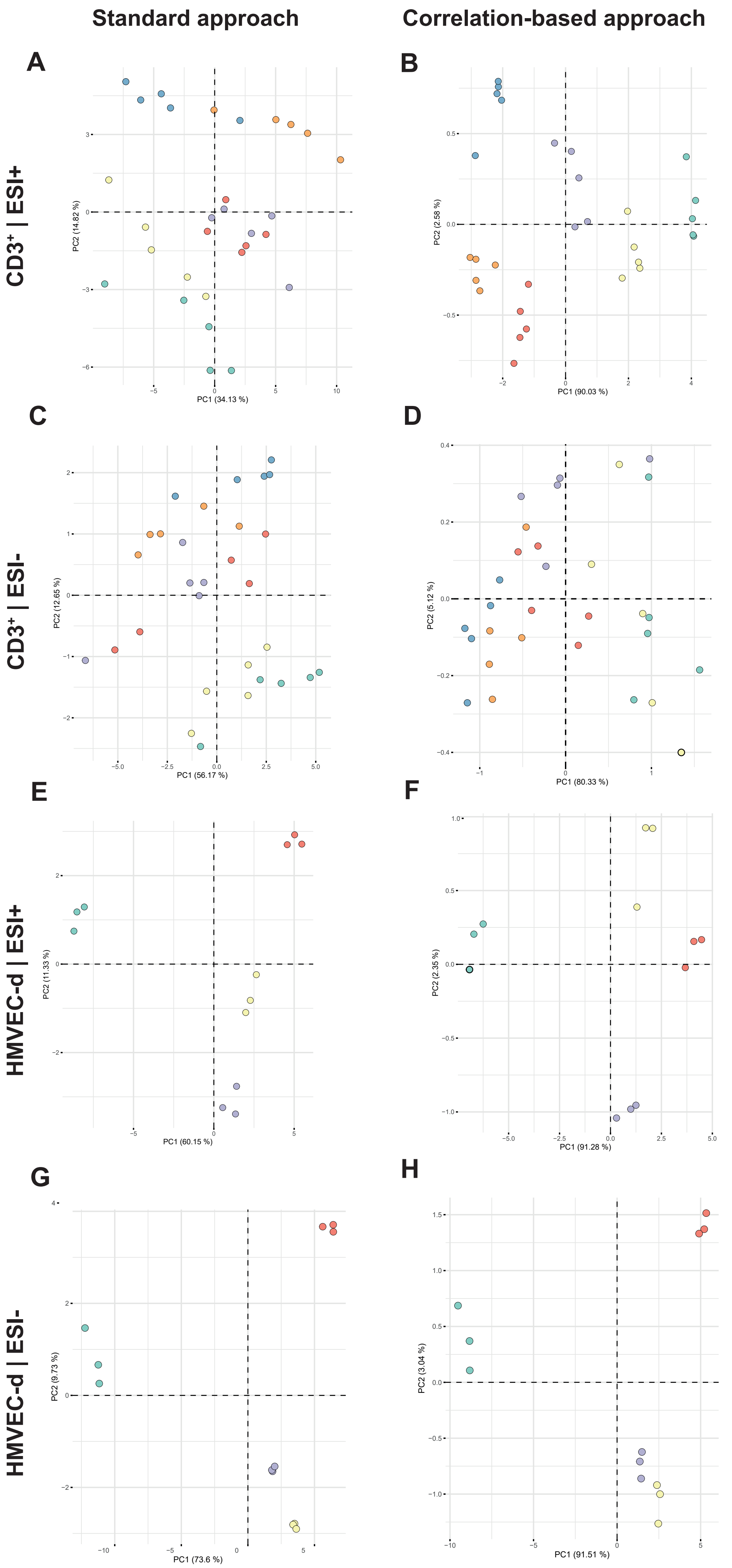

Standard approach
Correlation-based approach
A
B
C
D
E
F
G
H
CD3+ | ESI+
CD3+ | ESI-
HMVEC-d | ESI+
HMVEC-d | ESI-
PC1 (34.13 %)
PC2 (14.82 %)
PC1 (90.03 %)
PC2 (2.58 %)
PC1 (56.17 %)
PC2 (12.65 %)
PC1 (80.33 %)
PC2 (5.12 %)
PC1 (60.15 %)
PC2 (11.33 %)
PC1 (91.28 %)
PC2 (2.35 %)
PC1 (73.6 %)
PC2 (9.73 %)
PC1 (91.51 %)
PC2 (3.04 %)
